# Comparative regulomics of wood formation across dicot and conifer trees

**DOI:** 10.1038/s41467-026-75624-2

**Published:** 2026-07-22

**Authors:** Eduardo Rodriguez, Siri Birkeland, Ellen Dimmen Chapple, Samuel Fredriksson, Zulema Carracedo Lorenzo, Teitur Ahlgren Kalman, Vikash Kumar, Jamie Mccann, Jason Hill, Sivagamy Soundiramourtty, Aline Voxeur, Åsmund Kjendseth Røhr, Hannele Tuominen, Ewa J. Mellerowicz, Nathaniel R. Street, Torgeir R. Hvidsten

**Affiliations:** 1 Department of Plant Physiology, Umeå Plant Science Centre (UPSC), Umeå University, Umeå, Sweden; 2 Faculty of Chemistry, Biotechnology and Food Science, Norwegian University of Life Sciences, Ås, Norway; 3 Natural History Museum, University of Oslo, Oslo, Norway; 4 Department of Cell and Molecular Biology, National Bioinformatics Infrastructure Sweden, Science for Life Laboratory, Uppsala University, Uppsala, Sweden; 5 Université Paris-Saclay, Institut National de Recherche pour l’Agriculture, l’Alimentation et l’Environnement (INRAE), AgroParisTech, Institut Jean-Pierre Bourgin for Plant Sciences (IJPB), Versailles, France; 6 Department of Forest Genetics and Plant Physiology, Umeå Plant Science Centre (UPSC), Swedish University of Agricultural Sciences, Umeå, Sweden; 7 SciLifeLab, Umeå University, Umeå, Sweden

**Keywords:** Plant evolution, Gene regulatory networks, Comparative genomics, Plant genetics

## Abstract

Understanding the regulatory program underlying wood formation is key to improving biomass production and carbon sequestration in trees. However, how wood formation evolved and how these programs have been rewired across lineages remains unclear. Here, we present the first high-spatial-resolution evo-devo resource for wood transcriptomes spanning multiple dicots and conifers, representing the two major tree-containing lineages separated by more than 300 million years of evolution. Using orthology-aware co-expression network analysis, we identified genes with conserved and lineage-specific expression patterns. By integrating chromatin accessibility data and transcription factor motif analysis, we further inferred candidate regulatory networks for xylem differentiation and secondary cell wall formation. We demonstrate how this dataset can be used to answer long standing questions in wood biology related to differences in acetylation of cell wall polymers and master regulators of xylem specification across dicot and conifer tree species. The data offer a resource for the tree biology and evo-devo communities, and are publicly available at PlantGenIE.org.

## Abstract (web summary)

Understanding how wood formation is regulated could help improve biomass production and carbon sequestration in trees. Here the authors report the activity of genes underlying wood formation in six diverse tree species revealing shared and lineage-specific regulatory programs.

## Introduction

Trees are an essential component of terrestrial ecosystems, and wood is one of the planet’s largest sources of renewable biomass^1,2^. Beyond their ecological and economic importance, trees also play a key role in carbon sequestration, with wood serving as a long-term reservoir for storing the carbon fixed through photosynthesis^3^. In fact, forests comprise the second-largest carbon store on Earth after the oceans^4^. However, the regulatory mechanisms underlying wood formation remain incompletely characterized—especially the extent to which these mechanisms are shared or derived across diverse tree lineages. Bridging this knowledge gap is essential for uncovering fundamental principles of vascular development and for guiding efforts to enhance biomass production, long-term carbon capture and future materials.

Wood, or secondary xylem, is not only a fundamental structural and functional component of trees but also a model for studying the molecular mechanisms determining cell differentiation and tissue development. Wood formation arises from the activity of the vascular cambium, a narrow layer of secondary meristematic cells that produce secondary phloem towards the outer layers of the stem (i.e., towards the protective bark) and secondary xylem inwards^5^. Cambial cells differentiate primarily by cell enlargement followed by secondary cell wall (SCW) deposition. Although *Arabidopsis thaliana* (Arabidopsis) is an herbaceous plant, it does exhibit limited wood formation^6^ and has therefore been used as a model to study the gene regulatory networks underlying wood formation in trees^7^. In Arabidopsis, these networks consist of several NAC (NAM, ATAF1/2 and CUC2) domain transcription factors (TFs) that control specific MYB (myeloblastosis) TFs, which in turn regulate genes essential for SCW formation, such as SCW cellulose synthase (CesA) genes^8^.

While the fundamental wood cell types and processes are shared by most trees, significant differences exist between the two major tree-containing plant lineages: gymnosperms, of which conifers are the most diverse and well-studied group, and angiosperms^9^. Conifer wood, often referred to as softwood, is primarily composed of tracheids, which transport water and provide mechanical support. In contrast, angiosperm wood, or hardwood, evolved two additional types of xylem elements that serve specific functions; the vessel elements are dedicated to water transport, and the fibers provide structural support^10^. The chemical composition of cell wall polymers also differs between hard and soft wood types. For instance, lignin is generally less abundant in hardwood fibers than in softwood cells and differs in monomer composition: angiosperm lignin contains both guaiacyl (G) and syringyl (S) units (derived from coniferyl and sinapyl alcohols, respectively) with minor amounts of p-hydroxyphenyl (H) units, while conifer lignin contains predominantly G units with low amounts of H units, and typically lacks S units^9^.

In a seminal opinion piece, Groover (2005) suggested that changes in gene regulation, rather than unique tree genes, play a key role in determining differences in growth habits between herbaceous and woody plants^7^. This view is broadly supported by population and comparative genomics studies showing that phenotypic variation within and between species is largely driven by variants affecting gene regulation rather than protein coding sequences^11^. Birkeland et al. (2025) analyzed protein-coding sequence evolution and gene copy number variation in several evolutionary transitions from woody to herbaceous growth forms^12^. Consistent with the emphasis of Groover (2005) on regulatory factors, the candidate genes highlighted by Birkeland et al. included several transcription factors and chromatin-remodeling genes. However, they also showed that gene loss processes, such as relaxed selection and gene family contraction, affect genes involved in secondary growth and stress response, suggesting that factors beyond regulatory changes contribute to growth form differences.

To uncover the complex regulatory landscape of wood formation, several studies have applied high-spatial-resolution ‘omics technologies, including transcriptomics^13–16^, proteomics^17^, metabolomics^15,18^ and epigenomics^19^. More recently, single-cell transcriptomics has provided additional insight into the cellular complexity and developmental dynamics of wood-forming tissues^20,21^. However, to fully understand how wood formation evolved and how developmental programs have been rewired across lineages, evolutionary developmental biology (evo-devo) approaches are essential. Robust evo-devo studies combine high-spatial-resolution data across development with multiple species from each compared lineage. Such designs have the potential to identify how changes in gene regulation contributed to the emergence and diversification of wood formation^22^. While evo-devo has previously been used to, for example, compare gene expression between normal, tension and opposite wood^23,24^, this approach has not yet been applied using high-spatial-resolution data to study wood formation. In addition, the regulatory mechanisms underlying differences between angiosperm hardwood and gymnosperm softwood have not been systematically compared across multiple species per lineage. A comparative analysis of angiosperm and conifer species offers a powerful approach to identify key regulatory and developmental differences, as well as conserved components of the gene regulatory network underlying wood formation in trees. In this study, we leverage data on accessible chromatin and transcription factor binding with high-spatial-resolution transcriptomics data across wood-forming tissues in three dicots (the group of angiosperms that form wood via a bifacial vascular cambium) and three conifers to deepen our understanding of gene regulation during secondary growth, and to shed light on the evolutionary and functional dynamics of vascular tissue development in trees. Through this approach, we (1) reveal conserved and diverged gene expression patterns between the two lineages, (2) use these findings to answer long-standing questions in wood biology, and (3) compare candidate gene regulatory networks in representative dicot and conifer species.

## Results and discussion

### High-spatial-resolution wood transcriptomics across dicot and conifer tree species

**Fig. 1:**
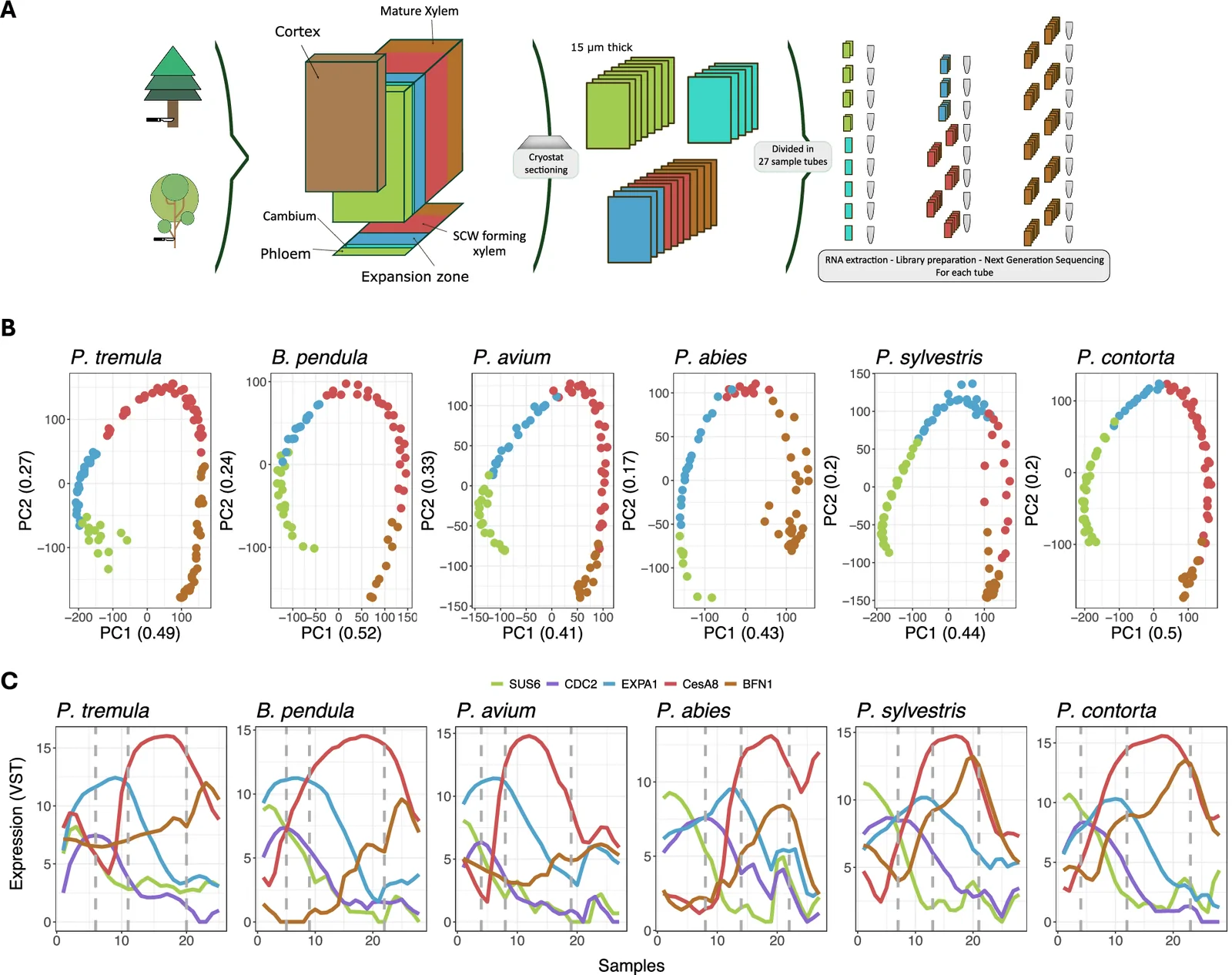
Sample collection and overview of gene expression across the different stages of wood formation. **A** Collection of wood samples and cryosectioning into 15-µm-thick layers. Sections were pooled into tubes for RNA extraction, library preparation, and sequencing. **B** Principal component analysis (PCA) of all samples across the replicate trees, colored by stages of wood formation as identified by hierarchical clustering (Supplementary Fig. 2). Percent explained variance in parentheses. **C** Expression profiles of genes marking the phloem (*SUS6*), cambial activity (*CDC2*), the expanding xylem (*EXPA1*), the SCW-forming xylem (*CesA8*), and mature xylem/cell death (*BFN1*). Dotted vertical lines mark, from left to right, the middle of the cambium, the end of the expansion zone and the end of the SCW zone.

We generated and analyzed high-spatial-resolution RNA sequencing (RNA-Seq) data spanning the secondary phloem, vascular cambium, and wood-forming tissues of three dicots (aspen/*Populus tremula* - using data from Sundell et al. (2017)^13^, birch/*Betula pendula* and cherry/*Prunus avium*) and three conifers (Norway spruce/*Picea abies*, Scots pine/*Pinus sylvestris* and lodgepole pine/*Pinus contorta*). To minimize genotypic variation, three clonal replicate trees per species (four in *P. tremula*) were sampled, with all replicates being mature trees growing in the field. First, 15-µm thick longitudinal tangential cryosections were obtained across developing phloem and wood-forming tissues (Supplementary Fig. 1). These cryosections were then pooled into 25–28 samples per replicate tree: single sections were used within the cambial meristem region, pools of three sections representing expanding and secondary cell wall (SCW)-forming xylem, and pools of nine sections capturing maturing xylem (Supplementary Data 1)^13^. Finally, gene expression datasets were created by performing RNA-seq on the pooled samples (520 samples in total, Fig. 1A).

By mapping RNA-seq reads to five reference genomes (both pine species were mapped to the Scots pine genome sequence^25^ as no reference is available for lodgepole pine), and estimating relative gene expression values for each gene, we obtained six species-specific transcriptomes with 11,000–20,000 genes expressed across 487 samples (Supplementary Data 2). Gene expression patterns were highly consistent across replicate trees within all six species (Supplementary Fig. 2), indicating that environmental variation had a limited impact on the developmental expression patterns captured in our study. Both unsupervised principal component analysis (Fig. 1B) and hierarchical clustering (Supplementary Fig. 2) largely reconstructed the original sampling order from expression data alone, confirming that the transcriptomes are structured by developmental position. Hierarchical clustering also revealed distinct stages of wood formation in all six species (Supplementary Fig. 2), largely congruent with anatomical observations of the phloem, the expanding xylem and the maturing xylem (Supplementary Data 1). Using five well-characterized marker genes, we identified the precise positions of three major transcriptome reprogramming events corresponding to (a) the dividing cambial cells located between phloem and xylem differentiation, (b) the end of cell expansion and the beginning of SCW formation, and (c) the end of SCW deposition (Fig. 1C, dotted vertical lines).

### Comparative wood transcriptomics

**Fig. 2:**
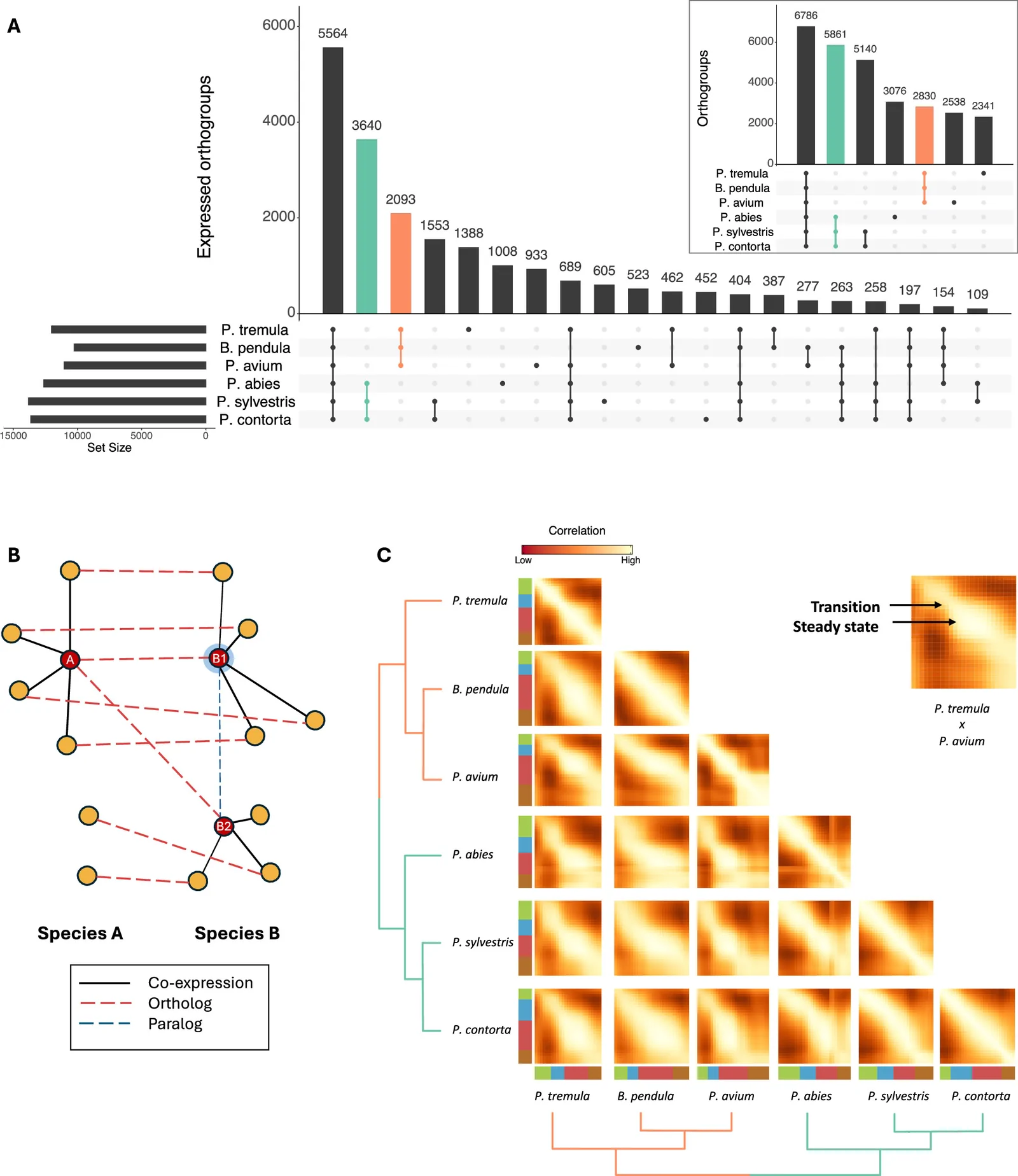
Comparisons of six wood transcriptomes. **A** Orthogroups (upper right corner): Upset plot showing the number of orthogroups with genes in each species (horizontal bars) and the number of orthogroups with genes in different subsets of species (vertical bars). Expressed orthogroups: Upset plot of the subset of orthogroups with expressed genes in wood. Bars representing lineage-specific orthogroups are colored orange (dicots) and green (conifers). **B** Method for identifying orthologs with conserved co-expression (co-expressolog). For each ortholog pair, a p-value is computed that reflects the degree to which their co-expressed genes are also orthologs. In this example, A and B1 are co-expressologs while A and B2 are not. **C** Heatmaps displaying the correlation of samples across species pairs, computed using co-expressologs. Only samples from one replicate tree are compared per species. Colors are scaled for each heatmap (each species pair). Color bars indicate the developmental stages: phloem (green), expanding xylem (blue), the SCW-forming xylem (red), and mature xylem/cell death (brown). The inset in the upper right corner shows one example: samples from *Populus tremula* on the x-axis (from phloem on the left to cell death on the right) and *Betula pendula* on the y-axis (from phloem at the top to cell death at the bottom). Squares of high correlation along the diagonal mark stages of wood development where gene expression changes relatively little (steady state), separated by transcriptome reprogramming events where massive changes occur (transitions).

To identify genes with conserved and diverged expression, we first identified ortholog groups (orthogroups) across 27 plant species based on protein sequence (see “Methods”). This resulted in 5564 orthogroups with genes expressed in wood in all six species, as well as 2,093/3,649 orthogroups with genes expressed in wood exclusively in dicots or conifers, respectively (Fig. 2A). Based on ortholog identification, we then compared gene expression across wood-forming tissues. However, because wood formation differs in anatomy and growth rate across species, samples at the same numeric order position in the series do not necessarily represent the same developmental stage, precluding approaches that rely on correlating expression values across matched samples. We therefore inferred co-expression networks independently for each species and identified co-expressologs: pairs of orthologous genes from two different species that are embedded in conserved co-expression relationships. Specifically, two genes are considered co-expressologs if they are orthologs and are co-expressed with other genes that are also orthologs of one another across species^26^ (Fig. 2B). This approach emphasizes conserved network context rather than direct correlation of expression profiles across aligned samples, which was the basis of the original expressolog definition^27^. Since co-expression typically only occurs between genes with smooth, developmentally regulated expression profiles, and these profiles need to be shared by a significant number of genes for a co-expressolog to be identified, this approach will not identify constitutively expressed genes or genes with rare expression profiles (typically genes with random spikes of expression) as co-expressologs. We identified approximately 3000–10,000 orthogroups with at least one co-expressolog per species pair (Supplementary Data 3), with a clear phylogenetic signal of stronger co-expression conservation within than between lineages (Supplementary Fig. 3A).

Using co-expressologs, we next analyzed the sample similarity among the six wood transcriptomes. This revealed that gene expression during wood formation is largely conserved across dicots and conifers, two lineages separated by more than 300 million years of evolution (Fig. 2C): samples from the same developmental stage were more similar to each other across species than samples from different stages within species (diagonals in Fig. 2C and bimodal distribution of correlations in Supplementary Fig. 3B). This analysis also showed a clear phylogenetic signal, with corresponding samples showing much higher correlation within dicots and conifers (~0.75) than between the two lineages (~0.55; Supplementary Fig. 3B). This analysis also revealed some of the same stages of wood formation, and the corresponding reprogramming events, seen in cluster analysis of genes (Supplementary Fig. 2), consistent with a suggested modular nature of secondary growth^28^.

### Genes with conserved expression across dicots and conifers during wood formation

**Fig. 3:**
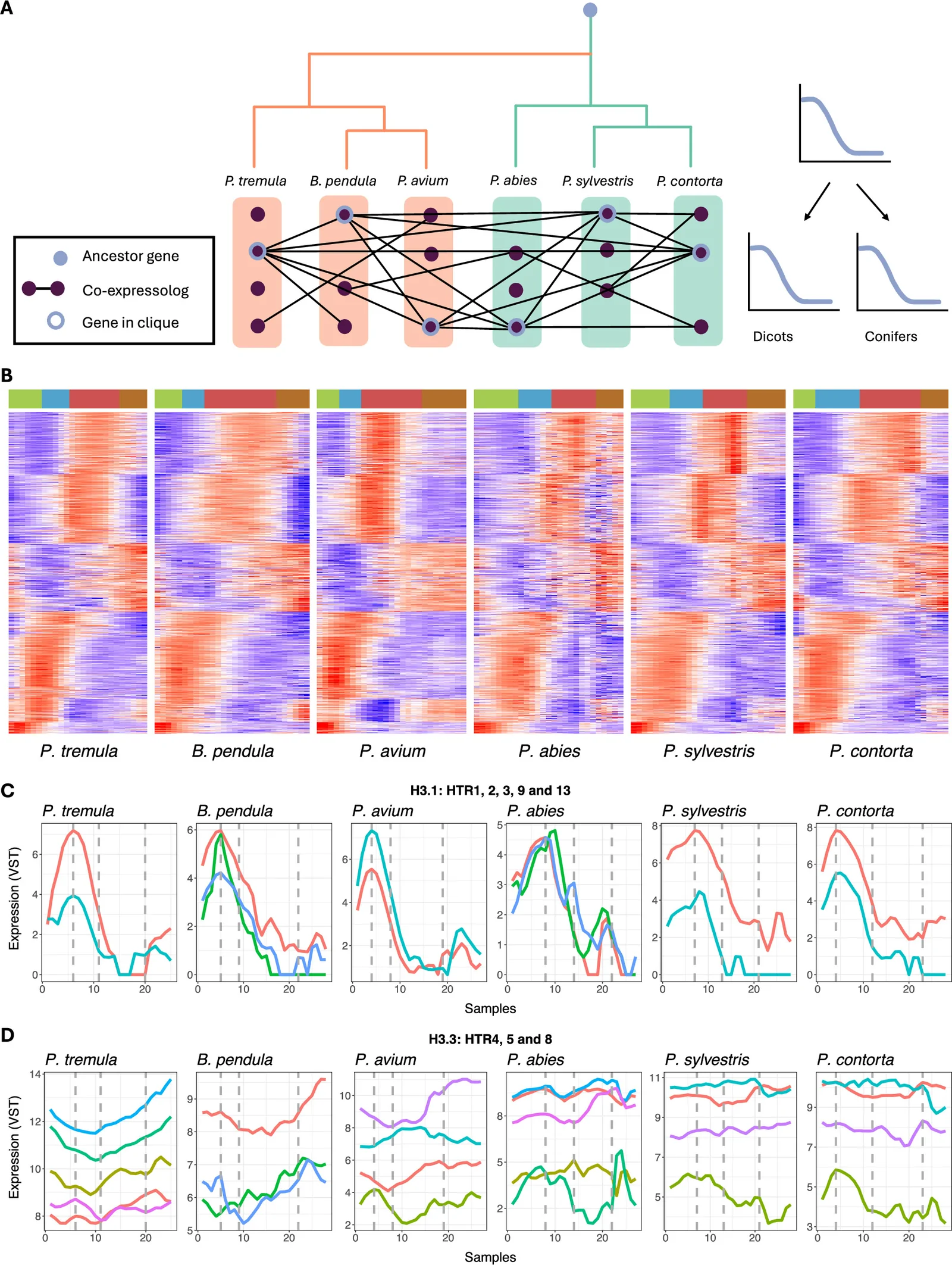
Genes with conserved co-expression across all species. **A** Conceptual figure of how we selected cliques of genes with conserved co-expression across all six species. A network was created for each orthogroup, connecting genes that are co-expressologs. Fully connected subnetworks with one gene from each species (i.e., cliques) were identified. Note that cliques from the same orthogroup can partly overlap. **B** Heatmap of unique cliques with conserved co-expression across all six species (i.e., non-overlapping cliques, selected by iteratively retaining only cliques with no genes already assigned to a previously selected clique). Each row is a clique of six genes, one from each species. Each column is a sample from one replicate tree from each species, and the top color bar indicates the wood formation stages. **C**, **D** One orthogroup (OG0000095) contained all Arabidopsis H3.1 (HTR1, 2, 3, 9 and 13) and H3.3 (HTR4, 5 and 8) genes. The gene tree is available in Supplementary Fig. 4. **C** Genes in top-cliques from the H3.1-clade of the orthogroup gene tree, showing conserved expression across all species. **D** All expressed genes in the H3.3-clade of the orthogroup gene tree. H3.1 is known to either lack introns (H3.1) or contain introns (H3.3) across the plant tree of life^80^, and genes not meeting these criteria were therefore removed. Dotted vertical lines in (**C**, **D**) mark, from left to right, the middle of the cambium, the end of the expansion zone and the end of the secondary cell wall zone. These lines correspond to the color bars in B.

To move beyond pairwise comparisons and make robust evolutionary inferences about wood formation, we next leveraged co-expressologs across all three dicot and three conifer species. To this end, we analyzed co-expressolog networks constructed within each orthogroup, where nodes represent genes and edges connect orthologous gene pairs that are co-expressologs. To identify gene sets with conserved expression across all six species, we searched for fully connected subnetworks, or cliques, containing exactly one gene from each species (Fig. 3A). This resulted in 70,458 cliques from 2098 orthogroups, of which 2145 cliques were non-overlapping (unique) (Supplementary Data 4). Hence, 38% of the expressed orthogroups (2098/5564, Fig. 2A) could be classified as having consistently conserved expression across all species, representing deeply conserved patterns of developmental gene expression regulation. Since co-expressologs are based on conserved co-expression and not expression profile similarity (Fig. 2B), these cliques could, in principle, reveal shifts in expression profiles, for example, between dicots and conifers. However, the set of 2145 unique cliques displayed strikingly similar expression profiles across the six species, where genes typically showed expression specific to the same stages of wood formation across all species (Fig. 3B). Conserved cliques covered major stages and processes of wood formation, as evident from expression profiles (Fig. 3B) and gene function enrichment analysis (Supplementary Data 5).

The species we studied have long divergence times, and the orthogroups typically contain multiple genes from each species and have gene phylogenies that are difficult to interpret, with genes often segregating into distinct dicot-specific and conifer-specific clades. Moreover, they often contain a large number of conifer genes due to extensive local duplications in these species^25^. Cliques identify orthologs with similar co-expression patterns across clades, pinpointing the most likely 1-to-1 ‘functional’ orthologs across species and thereby helping to resolve complex phylogenies. To illustrate this, we studied the orthogroup of H3.1 and H3.3 histones. H3.1 is incorporated during DNA replication and is thus linked to proliferating cells, while H3.3 is incorporated into chromatin in response to transcriptional activity, maintaining chromatin accessibility^29^. The gene tree of the histone orthogroup separated H3.1 and H3.3 histones into different clades, but it was not possible to establish 1-1 orthology between the characterized Arabidopsis genes and genes in the other species within these clades (Supplementary Fig. 4). However, we found a number of H3.1-cliques containing genes with clear expression peaks in the cambium of all six species (Fig. 3C). Hence, H3.1 marks the proliferative phase of wood formation, where cambial cells are undergoing mitotic divisions before differentiating into xylem or phloem, in agreement with the expectation that DNA replication is largely restricted to the cambium during wood formation. On the other hand, we did not identify any H3.3-cliques as these genes showed constitutive expression across the wood series, with no developmentally regulated expression profiles (Fig. 3D). This suggests ongoing chromatin remodeling in developing phloem and xylem, and indicates a role of epigenetic regulation in major transcriptome reprogramming events.

### Genes with diverged expression between dicots and conifers during wood formation

**Fig. 4:**
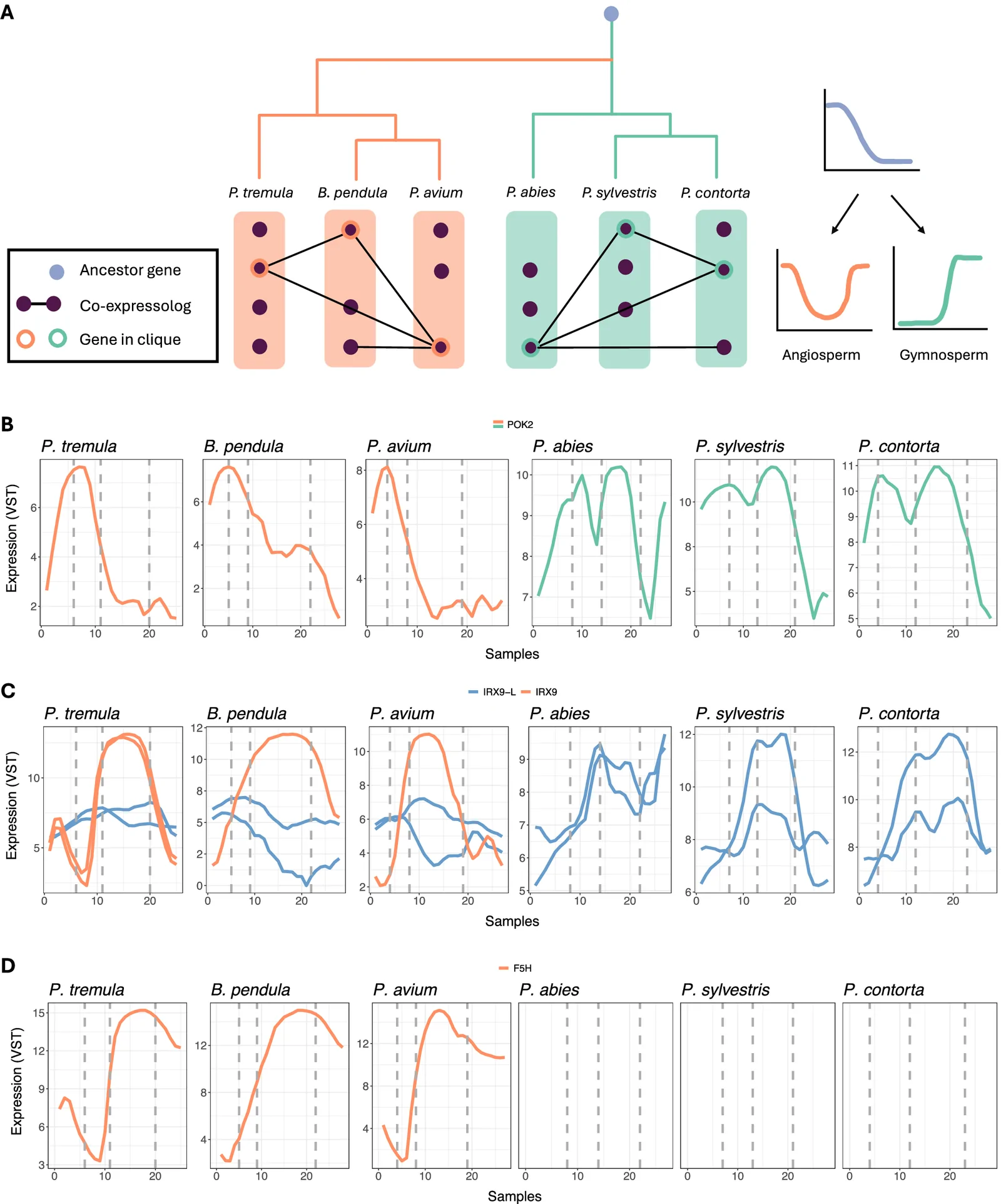
Genes with diverged expression between dicots and conifers. **A** Conceptual figure of how we selected cliques of genes with conserved co-expression (i.e., co-expressologs) within dicots and within conifers, but not between dicots and conifers. **B** Gene with diverged expression between dicots and conifers: *phragmoplast orienting kinesin 2* (*POK2*). The gene forms a dicot- and a conifer-specific clique, but does not have conserved expression across dicots and conifers. **C** Orthologs of *IRREGULAR XYLEM 9* (*IRX9*) form two overlapping dicot-specific cliques (same gene in B. pendula and P. avium). The paralog in Arabidopsis (*IRX9-L*) has two orthologs in each of the six tree species. **D** Orthologs of *FERULIC ACID 5-HYDROXYLASE* (*F5H*) form a unique dicot-specific clique. Dotted vertical lines in (**B**–**D**) mark, from left to right, the middle of the cambium, the end of the expansion zone and the end of the secondary cell wall zone.

Dicot and conifer wood have very different vascular structures and SCW chemical composition. To identify genes with diverged co-expression that could determine these differences, we first identified orthologs with conserved co-expression within, but not between, the two lineages (Fig. 4A). This approach ensures that inferred divergence reflects robust lineage-level differences observed consistently across multiple species, rather than pairwise absence of conservation that could be attributed to environmental noise. This resulted in 1810 pairs of dicot-/conifer-specific cliques from 293 orthogroups (Supplementary Data 4). These included genes enriched for pathways related to carbohydrate metabolism, nitrogen signaling, and intracellular trafficking (Supplementary Data 5), suggesting divergent strategies in resource allocation, metabolic coordination, and cellular remodeling during secondary growth. One illustrative example is the ortholog of *phragmoplast orienting kinesin 2* (*POK2*) (Fig. 4B). Phragmoplasts are specialized cytoskeletal structures that form during the late stages of plant cell division to build the cell plate—a partition between two daughter cells that precedes primary cell wall (PCW) deposition. While *POK2* showed peak expression in the cell division zone in both lineages, consistent with its known role in cell plate formation and alignment, it also displayed a second expression peak during secondary cell wall (SCW) formation in conifers only (Fig. 4B). The functional significance of this conifer-specific expression pattern remains to be determined.

We also analyzed the 2093/3640 orthogroups (Fig. 2A) that were either completely lineage-specific or that contained genes only expressed in wood within one lineage, identifying 1160 dicot-specific and 3045 conifer-specific unique cliques. Dicot-specific cliques included several genes relevant to wood formation, such as *xylem serine peptidase 1* (*XSP1*), *IRREGULAR XYLEM 8* (*IRX8*) and *IRX9*, *VND-interacting 2* (*VNI2*) and *FERULIC ACID 5-HYDROXYLASE* (*F5H*). *IRX8* and *IRX9* are key xylan end sequence and backbone-biosynthetic genes, respectively. *IRX9* and its paralog *IRX9L* have been proposed to form secondary and primary cell wall xylan synthase complexes, respectively^30^. Our data support that *IRX9* genes have a specialized role in SCW biosynthesis in dicots, with a clear peak during SCW formation, while *IRX9L* genes exhibited a more general expression pattern during xylogenesis, similar to that of pectin and xyloglucan biosynthetic genes (Fig. 4C). Syringyl lignin (S-lignin) is a hallmark of dicot wood, contributing to a more flexible and less condensed lignin polymer compared to the guaiacyl (G) lignin-dominated structure in conifer wood, with *ferulate 5-hydroxylase* (*F5H*) catalyzing the rate-limiting step in S-lignin biosynthesis^31^. The dicot-specific expression of *F5H*, with a profile resembling that of SCW cellulose synthase genes (Fig. 4D), is congruent with the absence of S-lignin in conifers. Conifer-specific cliques, on the other hand, included genes involved in pathogen defense (TMV resistance protein) and pentatricopeptide repeat (PPR)-containing proteins, which are responsible for RNA editing and that have experienced multiple lineage-specific duplications in conifers^32^.

### Acetylation of cell wall polysaccharides across dicots and conifers

Acetylation plays a crucial role in regulating cell wall properties by modifying the hydration status, enzymatic accessibility, and intermolecular interactions of cell wall polymers, which influence wall porosity, mechanical strength, and resistance to degradation. Trichome Birefringence-Like (TBL) proteins include key O-acetyltransferases responsible for acetylating specific cell wall polysaccharides during their biosynthesis in the Golgi, as well as recently identified acetyl esterases that deacetylate specific polysaccharides in cell walls^33^. Given the difference in hemicellulose acetylation and composition between angiosperm and conifer wood, differences in TBL family structure and activity are expected. In angiosperms, glucuronoxylan is the dominant hemicellulose, whereas (galacto)glucomannan is less abundant, and both of these hemicelluloses are acetylated^34^. In contrast, acetylated (galacto)glucomannan is the main hemicellulose in conifers, whereas arabinoglucuronoxylan is less abundant and non-acetylated^35^. Both lineages also contain low amounts of xyloglucan, which is known to be acetylated in angiosperms^36^, whereas its acetylation in conifers has not, to our knowledge, been analyzed.

**Fig. 5:**
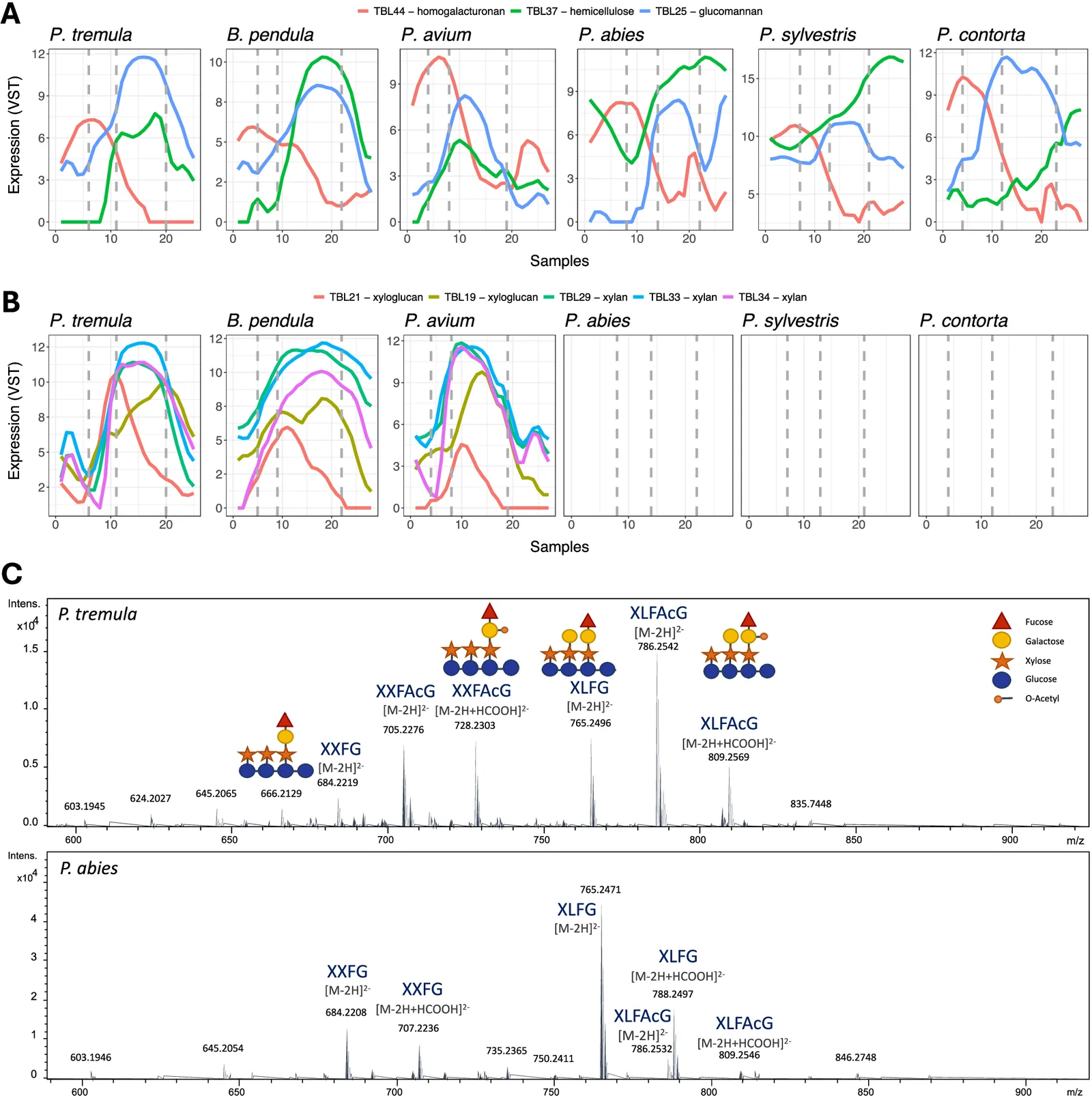
Trichome Birefringence-Like (TBL) gene expression. **A** TBLs with conserved expression across dicots and conifers. **B** Dicot-specific TBLs. Dotted vertical lines mark, from left to right, the middle of the cambium, the end of the expansion zone and the end of the SCW zone. **C** Spectra of oligosaccharides resulting from enzymatic fingerprinting of xyloglucans in the primary cell walls of *P. tremula* and *P. abies*. Intens: signal intensity; m/z: mass to charge ratio. Xyloglucan oligosaccharides follow the nomenclature described in Fry et al. (1993)^81^.

Analyzing the phylogeny of different orthogroups of the *TBL* family (Supplementary Fig. 5), we discovered that *TBLs* responsible for xylan (*TBL 3* + *28–35*) and xyloglucan (*TBL 19–22* + *27*) acetylation lack orthologs in conifers. We identified nine unique *TBL*-cliques containing genes with conserved co-expression across all six tree species (conserved) and five unique cliques specific to dicots (dicot-specific). Among the conserved *TBLs* (Fig. 5A), *TBL12* and *TBL44/PMR5*, characterized as both rhamnogalacturonan I (RGI)- and homogalacturonan (HG)-specific^37^ and only HG-specific^38^, respectively, showed expression peaks in the expansion zone, consistent with their role in pectin modification during primary cell wall formation (PCW). *TBL25*, which is specific to glucomannan^39^, peaked during SCW deposition and declined as lignification began, consistent with its role in glucomannan acetylation. Interestingly, *TBL37*, which is a jasmonate-induced *TBL* member responsible for unspecified hemicellulose acetylation^40^, showed a distinct pattern between the lineages: in dicots, it peaked during the SCW zone, whereas in conifers, its expression peaked later in the maturation zone, potentially indicating differences in jasmonate signaling between dicots and conifers.

Dicot-specific *TBL*s included those targeting the xyloglucan backbone (*TBL19*, *TBL21*^41^) and xylan (*TBL29*, *TBL33*, *TBL34*^42–46^) (Fig. 5B). As expected, *TBL21* showed increasing expression during cell expansion, consistent with a role in xyloglucan in PCW. In contrast, *TBL19* peaked much later, near the end of SCW formation, suggesting a possible role in xyloglucan biosynthesis in the protective layer that is formed at later stages of cell wall differentiation after SCW deposition^35^. The xylan-associated *TBL*s showed clear expression peaks during SCW deposition, supporting their role in xylan acetylation.

These findings suggest that core TBL functions in pectin and glucomannan acetylation are conserved across dicots and conifers, whereas xylan and xyloglucan acetylation are specific to dicots. To experimentally test the prediction that xyloglucan acetylation is absent in conifers, we performed enzymatic fingerprinting of xyloglucans^47^ through liquid chromatography–mass spectrometry (LCMS) analysis of xyloglucan oligosaccharides released by xyloglucanase digestion from *P. tremula* and *P. abies* samples containing primary cell wall material. Acetylated xyloglucan fragments (XXFAcG, XLFAcG) were abundant in *P. tremula*, whereas *P. abies* contained almost exclusively non-acetylated versions of these oligosaccharides, with only a trace of XLFAcG (Fig. 5C). This confirms a major difference in xyloglucan acetylation between the two species, though the trace of acetylation detected in *P. abies* suggests that low-level acetylation may occur through a TBL-independent mechanism. The presence of pectin-related TBLs in conifers further highlights the need for additional analysis of cell wall acetylation in these species.

### The regulatory network underlying wood formation

Our spatially resolved transcriptomes provide a valuable resource for investigating the regulatory logic controlling wood formation in trees. In Arabidopsis, the regulatory network controlling SCW formation is organized into three hierarchical layers of transcription factors with overlapping functions: a top layer of NAC master regulators *VASCULAR-RELATED NAC-DOMAIN* (*VND1–7*) and *NAC SECONDARY WALL THICKENING PROMOTING FACTOR* (*NST1–3*); a middle layer consisting of R2R3-MYB transcription factors MYB46 and MYB83; and a bottom layer that includes additional MYBs and *SECONDARY WALL-ASSOCIATED NAC DOMAIN* (*SND2 and 3*)^48,49^.

**Fig. 6:**
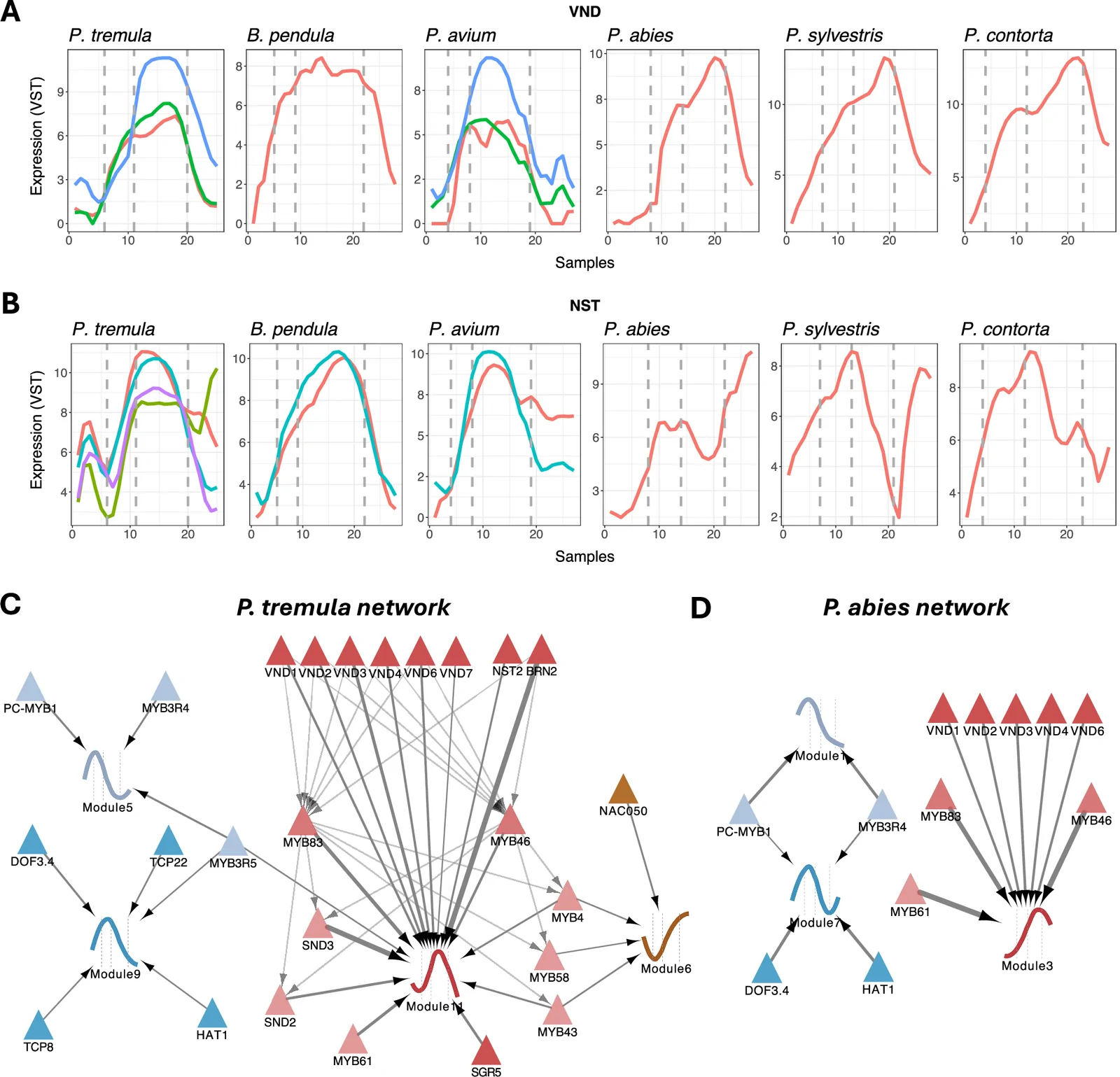
The Gene Regulatory Network (GRN) of *Populus tremula* and *Picea abies.* **A** Conserved clique of VND master regulators. **B** Expression profiles of NST master regulators. Dotted vertical lines in (**A**, **B**) mark, from left to right, the middle of the cambium, the end of the expansion zone and the end of the secondary cell wall zone. **C**, **D** The *P. tremula*/*P. abies* regulatory network restricted to the modules of the five marker genes (displayed using the expression profile of the central gene in the module) and a selected set of top-scoring characterized regulators (triangles). Neither network contained regulators of the phloem module, while the secondary cell wall (SCW) and cell death modules were merged in *P. abies*. The width of the links indicates the strength of the inferred regulatory interaction, combining the strength of the enrichment of the TF motif in the module and the strength of the expression similarity between the TF and the module. Regulators are colored according to the module with the strongest inferred regulatory interaction. SCW regulators are organized into a hierarchy of three layers (indicated by transparency), and links between layer 1 and 2, and layer 2 and 3 are based on the occurrence of individual motifs. The orthogroup containing Arabidopsis *NST 1-3* also included *BRN 1-2* (*BEARSKIN 1-2*) and *SMB* (*SOMBRERO*) (Supplementary Fig. 6). While the closest orthologs of BRNs and SMBs in our six species were not expressed in wood, the *BRN2*- and *NST2*-binding motifs were highly similar and, in our network analysis, linked to the same NST gene in *P. tremula* (*Potra2n2c4785*, purple gene in panel B) and *P. abies* (*PA_chr11_G001721*, single gene in panel B). We therefore included *BRN2* in the network as a potential binding site for NST.

In Arabidopsis, VNDs control the formation of the SCW in vessels, while NSTs control SCW formation in fibers^48^. We found that orthologs of VNDs (Supplementary Fig. 6) exhibited conserved expression patterns across all six tree species, with a distinct expression peak during SCW formation (Fig. 6A). In dicots, orthologs of NSTs (Supplementary Fig. 6) displayed a similar expression peak during SCW formation, while in conifers the single NST-copy had a pronounced dip in expression at this stage (Fig. 6B). Consistent with what was previously observed in a pairwise comparison of *P. tremula* and *P. abies*^14^, our data thus provide strong evidence that NSTs have diverged in expression between dicots and conifers.

To gain further insight into the regulatory role of these SCW regulators, we generated Assay for Transposase-Accessible Chromatin using Sequencing (ATAC-seq) data from *P. tremula* and *P. abies* (Supplementary Fig. 7). Unlike the spatially resolved RNA-seq data, the ATAC-seq was performed on bulk xylem scrapings representing predominantly developing xylem depositing secondary cell walls. While the ATAC-seq identifies regions that are accessible at some point during wood formation, the spatially resolved expression data provide the temporal dimension. We combined these two data types to build regulatory networks by mapping characterized DNA sequence patterns recognized and bound by plant TFs (i.e., TF DNA binding motifs) to open chromatin regions in the two tree genomes. Each putative binding site was linked to the closest gene (target) and the motif was associated with a co-expression network module (i.e., a set of co-expressed genes, Supplementary Fig. 8) if its target genes were statistically significantly enriched in the module. Finally, we used orthogroups and co-expression to assign a specific TF gene to the motif (see “Methods”). The resulting candidate TF-module networks linked 175/165 TFs to 13/8 modules containing 8228/5489 genes in *P. tremula* and *P. abies*, respectively (Supplementary Data 6). As expected, the candidate regulatory networks did not contain phloem-specific regulation as phloem cells were not well-represented in the ATAC-seq samples.

In *P. tremula*, the co-expression module containing the SCW *CesA* genes (module 11) was strongly preferentially associated with TFs known from the Arabidopsis SCW regulatory network, including the top layer master regulators VND and NST, the middle layer MYB46 and MYB83 and several bottom layer MYBs as well as SND2 and SND3 (Fig. 6C). Three bottom layer MYBs were also predicted to regulate the mature xylem module containing the cell death marker gene (module 6), indicating a previously unknown mechanism coordinating SCW formation and cell death/lignification (Fig. 6C). Several of the core SCW master regulators including VNDs, MYB46 and MYB83 were also associated with the corresponding SCW module in *P. abies* (module 3, Fig. 6D), thus suggesting conserved gene targets and a common origin of the regulatory network of wood formation. However, we did not find evidence of VNDs regulating the middle layer in *P. abies*: ATAC-seq profiles showed that the MYB46/83 regulatory regions are accessible in both species, but VND/NST binding motifs are absent in *P. abies* (Supplementary Fig. 9), suggesting that this regulatory link may have been lost at the sequence level in *P. abies*.

Next, we specifically investigated whether the top candidate regulators of the SCW *cesA* genes differ between dicots and conifers. In *P. tremula*, *SND3*, *VND3*, and *NST2* emerged as the strongest candidates, showing both high expression correlation with the SCW *cesAs* (*R* > 0.85) and predicted binding sites associated with all five SCW *cesAs* (Supplementary Data 6). In *P. abies*, *MYB83* was the strongest candidate, with high correlation (*R* = 0.88) and predicted binding sites associated with all three SCW *cesA* genes, followed by *MYB46* and several *VNDs* (Supplementary Data 6). In agreement, AlphaFold3-predicted TF-DNA complexes supported NST binding to SCW CesA regulatory regions in *P. tremula* but not in *P. abies*, while MYB83 binding was confirmed in *P. abies* (Supplementary Fig. 10C).

Evidence from animals suggests that regulatory complexity often arises through diversification of TF interactions^50^. To explore whether dicot and conifer species contrast in the complexity of TF interactions, we used AlphaFold3^51^ to predict TF-TF complexes, finding that master regulators in Arabidopsis and *P. tremula* were predicted to frequently interact—including across regulatory layers—whereas such interactions were much less common in *P. abies* (Supplementary Fig. 10B, Supplementary Data 6).

Taken together, the structure of the candidate *P. tremula* regulatory network mirrors that of Arabidopsis, adopting a layered architecture with feed-forward loops linking VNDs/NSTs, MYBs/SNDs, and biosynthetic genes. In contrast, the *P. abies* network appears notably flatter: VNDs and MYBs act more in parallel, with MYBs showing the strongest predicted regulatory interactions with biosynthetic targets, while NSTs appear unlikely to serve as primary regulators of SCW *cesAs* in this lineage. These observations are supported by predicted protein-DNA interactions. Overall, our analysis is consistent with a more streamlined network in *P. abies* compared to the more complex, layered network in *P. tremula* and Arabidopsis - a difference in complexity that is independently mirrored by predicted protein-protein interactions among the master regulators. Whether this reflects a general distinction between tracheid-only conifers and vessel-bearing dicots remains to be tested. These findings provide new insight into the regulation of SCW formation in conifers and highlight specific *P. abies* MYBs as promising targets for biotechnology and breeding efforts to tailor wood properties.

### Limitations

The tree species analysed in this study were sampled across different field sites and years, which could introduce additional environmental variation between species compared to a common-garden design. Our evolutionary inferences, however, rest only on positive findings—co-expression conserved across all species, or consistently conserved within a lineage—where the co-expression itself is structured primarily by the wood formation gradient. Environmental variation is therefore far more likely to obscure true conservation than to generate spurious concordance. Within each species, replicate trees are clonal ramets of a single genotype, minimizing genotypic variation while confirming the reproducibility of developmental expression programs. Population-level variation was not sampled, so our data do not capture eQTL-driven co-expression present in some genotypes but absent in others.

The candidate regulatory networks represent a two-species comparison (*P. tremula* vs. *P. abies*) rather than a replicated dicot-versus-conifer design. These should therefore be understood as species-specific candidate networks rather than as established lineage-level generalizations, supported by multiple converging lines of evidence: open chromatin accessibility from bulk xylem scrapings, motif enrichment in modules of co-expressed candidate target genes, co-expression between regulator and target, and predicted protein–DNA interactions.

### A resource for wood biology

Our datasets provide an unprecedented resource for wood biology, giving the community the means to address long-standing questions and generate insights beyond the scope of this study. Our comparative analyses demonstrate how the resources can pinpoint biochemical and gene-family differences (e.g., acetylation-related TBL paralogs), thus generating testable hypotheses for targeted experiments. All expression data are publicly available through the PlantGenIE web portal, providing an accessible platform for evo-devo studies of wood formation and plant evolution. Beyond basic research, this resource offers a foundation for practical applications, from breeding and engineering strategies for biofuels and wood-derived materials to long-term carbon storage.

## Methods

### Sampling

Wood samples from *Betula pendula*, *Pinus sylvestris*, and *Picea abies* were obtained at a clonal field trial in Skogforsk, Sävar (63.895648°N, 20.549005°E). *Betula pendula* samples from genotype 18 were taken on July 8th, 2020, between 10:30–13:30. *Pinus sylvestris* samples from genotype 29 were taken on July 8th, 2020, between 13:30–14:30. *Picea abies* samples from genotype K27-2125 were taken on July 10th, between 10:30–12:00. Wood samples from *Pinus contorta* from genotype 86 were obtained at the clonal field trial in Fagerlund (plot 7114, 64.083393°N, 19.793286°E), on July 9th, 2020, between 10:30–12:00. Wood samples from *Prunus avium* from genotype S21-K946 6006 were obtained from Skogforsk, Ekebo (55.950860°N, 13.112653°E), on June 28th, 2021, between 10:00–12:00. No obvious abiotic or biotic stresses were observed at the time of sampling.

Rectangular wood blocks were collected from each tree, immediately placed in dry ice boxes and stored at −70 °C. Prism-shaped pieces, containing the main layers of wood from cortex to mature xylem, were cut from the wood blocks using an electric saw. These pieces were placed into a cryo-microtome, and multiple longitudinal sections 15 µm thick were cut from each layer^52^. Only pieces with clearly discernible developmental layers were used. During the cutting process, cross sections were frequently prepared and studied under a light microscope to characterize the different tissue each section originated from (annotation in Supplementary Data 1). Between 120 and 150 longitudinal sections were prepared from each prism-shaped piece, covering the entire current year’s growth and the main developmental layers: phloem, cambium, expanding xylem, SCW-forming xylem and mature xylem. Each section was stored in a separate 1.5 ml Eppendorf tube and stored at −80 °C. This was repeated three times for a total of three replicates per tree.

### RNA extraction

For each replicate tree, sections were pooled together as shown in Tab S1 and thawed in QIAzol lysis reagent (Qiagen). Total RNA was extracted separately from each section pool using a protocol adapted from Chang et al. (1993)^53^ and the Qiagen RNeasy Kit (Qiagen, Hilden, Germany), with modifications following Schrader et al. (2004)^54^. Extraction buffer containing 2% CTAB, 100 mM Tris-HCl (pH 8.0), 25 mM EDTA, 2.0 M NaCl, and 0.5 g/L spermidine was prepared and pre-warmed. The Eppendorf tubes with section tissue samples were taken from −80 °C storage onto dry ice. At the start of the extraction protocol, the extraction buffer was added to the Eppendorf tubes and the tissue samples were dissolved. Chloroform:isoamylalcohol was added, and phases were separated by centrifugation at 10,000 rpm for 10 min. The supernatant was transferred to a new tube without disturbing the interphase, and the extraction step was repeated. RNA precipitation was carried out by adding 2.0 volumes of ethanol and incubating for 1 h at 4 °C, followed by centrifugation at 14,000 rpm for 20 min at 4 °C, resulting in a pellet.

The extraction continued with the Qiagen RNeasy Mini Kit. The RNA pellet was dissolved in 350 µL of RLT buffer from the Qiagen kit, added 1 volume of 70% ethanol, transferred to the column tube and spun at 10000 rpm for 15 s. The column was then washed with 350 µL of buffer RWT and spun the same way.

A DNase treatment was performed by mixing 10 µL of DNase I stock solution with 70 µL of buffer RDD, which was loaded onto the column and incubated at room temperature for 30 min. The column was washed and spun once with 350 µL of buffer RWT and twice with 500 µL of buffer RPE. The column was dried by centrifugation at 12,000 rpm for 5 min. The final RNA was eluted by adding 35 µL of RNase-free water and spinning at 10,000 rpm for 1 min. Total RNA from each section pool was quantified using the RNA High sensitivity program in the Qubit 2.0 fluorometer (Life Technologies, Carlsbad, California, USA).

### RNA library preparation and sequencing

The Universal Plus mRNA-Seq with NuQuant protocol (Tecan, Männedorf, Switzerland) was used to prepare cDNA libraries from the extracted RNA of each of the section pools according to the provided protocol. Following adapter ligation, the cDNA libraries were amplified by PCR and purified using Agencourt AMPure XP beads (Beckman Colter, Brea, California, USA).

Final concentration was measured using Qubit RNA High sensitivity, and quality was studied with the Agilent Bioanalyzer 2100 (Agilent Technologies, Santa Clara, California, USA) using Pico chips.

The libraries were sequenced on NovaSeq PE150 to a depth of 20 million pair-end reads.

### Read mapping and expression quantification

The resulting reads were quality checked and filtered using a publicly available preprocessing pipeline for RNA-seq data implemented at the Umeå Plant Science Center (v0.4.0, https://github.com/UPSCb). First, individual quality reports were generated for each sample using FastQC v0.11.9^55^ and summarized for each species with MultiQC v1.11^56^. We filtered out ribosomal RNA with SortMeRNA v4.3.4^57^, and used Trimmomatic v0.39^58^ to remove adapters, and trim reads with a 5 bp sliding window and a quality threshold of 30. For each species, filtered and trimmed reads were mapped to the reference with the alignment-free quantification tool Salmon v1.6.0^59^. We enabled options for modeling position-specific fragment start distribution, sequence-specific bias correction, and GC-content bias correction. For the dicot species, we additionally used a decoy-aware index to improve mapping accuracy; this was not feasible for the conifer species due to the large genome sizes. The following CDS and genomes versions were used in the analyses: *Populus tremula* v2.2^60^, *Betula pendula* v1.2 (earlier genome version used after recommendation from the authors^61^), *Prunus avium* v2.0^62^, *Picea abies* v2.0^25^, and *Pinus sylvestris* v1.0 (used for both *P. sylvestris* and *P. contorta*)^25^.

For each tree species, read counts were subjected to a moving average smoothing (within each replicate tree) before normalization using the Variance Stabilizing Transformation (VST) as implemented in DESeq2 v1.48.1^63^. Samples were visually inspected using hierarchical clustering and principal component analysis (PCA), and outliers breaking the expected smooth developmental gradient were removed iteratively (i.e., normalization was redone with samples removed, and reinspected). Removed samples are marked in Supplementary Data 1: 18 samples in *B. pendula* and 15 samples in *P. abies*. Genes with VST normalized expression values > 3 in two samples in three replicate trees (two replicate trees in *B. pendula* and *P. abies* due to the large number of samples removed from one tree) were defined as expressed. Hierarchical clustering was conducted using the R-function *hclust* with parameter *method* = *“ward.D2”* and Pearson correlation. PCA was done using the R-function *prcomp*.

### Orthogroups

Orthogroups were inferred using OrthoFinder v2.5.2^64^, based on the longest protein-coding sequence per gene from annotated genomes of 27 plant species, and incorporating a rooted species tree obtained from TimeTree. The 27 species were selected to broadly represent the major plant lineages with available genomes and included *Amborella trichopoda*, *Arabidopsis thaliana*, *Beta vulgaris*, *Betula pendula*, *Camellia sinensis*, *Cinnamomum kanehirae*, *Cucumis sativus*, *Eucalyptus grandis*, *Ginkgo biloba*, *Malania oleifera*, *Malus domestica*, *Medicago truncatula*, *Mimulus guttatus*, *Nicotiana tabacum*, *Physcomitrella patens*, *Picea abies*, *Pinus sylvestris*, *Pinus tabuliformis*, *Populus tremula*, *Prunus avium*, *Salix purpurea*, *Sequoiadendron giganteum*, *Taxus chinensis*, *Torreya grandis*, *Vitis vinifera*, *Welwitschia mirabilis*, and *Zostera marina*. We used both standard orthogroups (OGs) and high-resolution Phylogenetic Hierarchical Orthogroups (HOGs: N1 with *Physcomitrella patens* as the outgroup). Statistics reported in the paper are for HOGs. The gene content of the orthogroups was visualized using the *UpSetR* package^65^.

### Gene co-expression analysis

#### Identifying co-expressologs using ComPlEx

Co-expression networks were generated for each species using Pearson’s correlation and Mutual Rank (MR) normalization, as implemented in the popular PlaNet method^66^. The networks were constrained to include only the top 3% most strongly co-expression links. This threshold was chosen based on a saturation analysis showing that most co-expressologs are gained between 1% and 3% (Supplementary Fig. 11), and on visual inspection confirming that well-characterized SCW gene families were detected as co-expressologs at this threshold.

For each of the 15 pairs of species, co-expression neighborhoods of orthologs were compared using the ComPlEx method^26^. Briefly, given an ortholog pair A-B, the co-expression neighbors of ortholog A were mapped to their ortholog(s) in the network of ortholog B, and the overlap between the mapped neighbors of A and the neighbors of B was assessed using the hypergeometric test. Similarly, the neighbors of B were mapped to the network of A. This resulted in two *p*-values, one using the network of ortholog A as the background and one using the network of ortholog B. We required both False Discovery Rate-corrected p-values to be below 0.1 to classify the pair as a *co-expressolog*.

#### Sample comparison

The expression similarity of samples coming from different species were visualized using sample correlation heatmaps. For each species pair, the expression similarity of each sample pair was quantified by computing Pearson’s correlation of the expression values of all co-expressologs. Since a gene could occur in multiple co-expressolog-pairs, duplicates were removed, retaining the co-expressolog pair with the lowest FDR-value. Heatmaps were generated using the ComplexHeatmap package.

#### Identifying cliques

For each orthogroup, a network was created where orthologs were connected by links with weights set to the largest co-expressolog FDR-value. Cliques were then identified using the Igraph package v2.1.2 and the function *max_cliques*. First, genes conserved across all six species were identified as six-member cliques (i.e., including one gene from each of the six species). We included both “complete conserved cliques” requiring all 15 ortholog-pairs to be co-expressologs (FDR < 0.1) and “partial conserved cliques” requiring at least 11 ortholog-pairs to be co-expressologs (with all gene pairs required to have FDR < 0.9). To account for missing gene annotations in individual species, we also classified five-member cliques as “partial conserved cliques” if all ortholog-pairs were co-expressologs. Differentiated cliques were identified as six-membered cliques where all three lineage-specific ortholog-pairs were co-expressologs and no more than six inter-lineage pairs were co-expressologs. Lineage-specific cliques were identified from orthogroups containing only dicot or conifer expressed genes, and were required to be complete, three-membered cliques.

Conserved cliques (complete and partially significant) were visualized using the ComplexHeatmaps package using correlation as a distance metric and clustering using the ward.D2 method. Scaling and centering of gene expression were performed individually for each species prior to clustering.

#### Enrichment analysis

Gene ontology enrichment analysis was performed for the conserved and differentiated gene sets as well as for clusters of conserved genes using the GSEABase and GOstas packages. The analysis was based on annotated *Populus tremula* genes downloaded from plantgenie.org. A Fisher exact test was performed using the *GSEAGOHyperGParams* function, setting ontology to biological process (BP) and using a p-value cutoff of 0.05. The significant GO terms were summarized using Revigo^67^ and visualized as treemaps. Segment sizes were based on the *values* column containing -log10-transformed enrichment *p*-values.

### Regulatory networks analysis

#### Transcriptional module detection

To infer robust regulatory networks, we based our transcription factor (TF) motif analysis on modules of co-expressed genes rather than individual genes. Modules were detected using a coverage algorithm previously applied to high-spatial-resolution wood transcriptome data^13^. This approach identifies non-overlapping modules centered on the most highly connected genes in the network, and was shown to capture the continuum of active processes across the wood-forming tissues^13^. Briefly, we iteratively selected the most highly connected genes in the co-expression network (centroids) with neighbors (top 1%) not overlapping previously selected neighborhoods. Genes were then assigned to their closest centroid if the link was in the top 3%.

#### Open chromatin regions: Nuclei for ATAC-sequencing

The scrapings of developing xylem from *P. tremula* and *P. abies* trees were powdered by grinding in liquid nitrogen. The nuclei were purified using a Percoll gradient method^68^. In brief, 2 g each of grounded tissues in were resuspended in 20 ml of 1X nuclei isolation buffer (NIB) [10 mM MES-KOH (pH 5.4), 10 mM NaCl, 10 mM KCl, 2.5 mM EDTA, 250 mM sucrose, 0.1 mM spermine, 0.5 mM spermidine, 1 mM DTT] supplemented with 2% (w/v) Polyvinylpyrrolidone-40 (PVP-40, Sigma-Aldrich) and 0.1% Protease inhibitor cocktail (Roche) just before extraction. The homogenates were gradually filtered through two layers of pre-wetted cheesecloth and one layer of Miracloth. In filtrate, Triton-X100 was added in a dropwise manner to make the concentration 1% (v/v) and incubated for 20 min at 4 °C under gentle rotation. The homogenates were centrifuged 2000 x *g* for 10 min at 4 °C and pellets were resuspended in 5 ml 1X NIB with the help of paint brush. The crude preparation of nuclei suspension was carefully loaded on top of the pre-assembled density gradient (3 ml 60% Percoll in 1x NIB and 3 ml of 2.5 M Sucrose) by slowly releasing the solution onto the side of the tube above the 60% Percoll layer with the help of a Pasteur pipette. The assembled gradient is subject to centrifugation in a swinging bucket rotor at 1200 × *g* for 30 min at 4 °C without breaks. The rest of the nuclei purification steps were kept as described for *Rosaceae* in^68^. The DAPI (0.5 μg/ml) stained nuclei were counted using a Neubauer chamber (Sigma-Aldrich) under an Axioplan 2 (Ziess) microscope with a DAPI filter, and for *P. tremula* 40,000 and for *P. abies* 30,000 nuclei were used for ATAC-seq library preparation.

The ATAC-seq libraries were prepared after optimizing the amount of Tn5, number of nuclei and time of incubation for each species, as explained in Picelli et al. (2014)^69^ for in-house produced Tn5. Further 40,000 and 30,000 nuclei from *P. tremula* and *P. abies* were tagmented for 30 min at 37 °C with 3 µl of adapter-loaded Tn5, tubes were flipped after each 10 min of incubation. The tagmented DNA was purified using MinElute PCR Purification Kit (Qiagen Cat. No. / ID: 28004) and eluted in 12 µl nuclease-free water. The eluted DNA was used for sequencing library preparation using indexing primers, Illumina Nextera Library Prep Kits and the size-selected libraries were sequenced on NovaSeq 6000 in pair-end mode.

#### *P. tremula* and *P. abies* ATAC-seq pre-processing

Trimmomatic v0.39^58^ was used to perform quality trimming on raw paired-end reads with the setting: ‘ILLUMINACLIP:$TRIMMOMATIC_HOME/adapters/NexteraPE-PE.fa:2:30:10:1:TRUE SLIDINGWINDOW:5:20 MINLEN:38’. Trimmed reads were aligned to the P. abies and P. tremula reference genomes for their respective datasets using Bowtie2 v2.4.5^70^ with the setting ‘--very-sensitive --dovetail --maxins 1000’. Alignments were filtered using Samtools v1.16^71^ with ‘-f 3 -F 12 -q 20’ to retain high-quality, properly paired reads (MAPQ ≥ 20), followed by PCR duplicate removal with Samtools markdup. Peaks were called using MACS3 v3.0.0b1^72^ with the setting ‘--format BAMPE’ and genome sizes of ‘--gsize 17.7e9’ for P. abies and ‘--gsize 3.8e8’ for P. tremula. The ‘--keep-dup all’ option was used, as PCR duplicates had already been removed prior to peak calling. TSS enrichment scores and fragment length distributions were assessed using the ATACseqQC R package (version 1.32.0; default parameters)^73^. Coverage tracks over genes of interest were generated from quality-filtered and deduplicated BAM files using the Gviz R package (version 1.52.0)^74^. ATAC-seq signal was visualized using deepTools v3.5.6. bamCoverage was run with --normalizeUsing CPM --effectiveGenomeSize 17683410177 / 381973357 (P. abies / P. tremula) --binSize 100 --centerReads to generate a normalized BigWig file. computeMatrix was run in reference-point mode with --beforeRegionStartLength 2000 --afterRegionStartLength 2000 using gene TSS positions in *P. abies* / *P. tremula*, respectively. The resulting matrix was visualized with plotHeatmap.

#### Inference of Gene Regulatory Network (GRN)

We compiled 808 TF motifs from the JASPER^75^ and PlantTFDB databases^76^, and linked an Arabidopsis TF to each motif using a combination of BLASTp and the name of the motif. The motifs were mapped to open chromatin regions in *P. tremula* and *P. abies* using the Find Individual Motif Occurrences (FIMO) tool in the MEME suite, version 5.5.5^77^. Motifs were assigned to the closest gene within 10 kbps (target genes), and a motif was assigned to a network module if the target genes of that motif were enriched in the module (Fisher's exact test, FDR-corrected *p*-value < 0.05). Given that a motif was enriched in a module, we searched the orthogroup of the associated Arabidopsis TF for the *P. tremula*/*P. abies* TF with the highest expression correlation to the average expression profile of the module. The *P. tremula*/*P. abies* TF were assigned to the motif/module if the Pearson expression correlation > 0.7 and the corresponding FDR-corrected *p*-value < 0.05. Hence, each TF-module association in the final GRN was based on (1) significant enrichment of the TF’s motif in the module and (2) significant expression correlation between the TF and the module. All significant associations were listed in Supplementary Data 6 and sorted by the score: *-log10(Motif enrichment p-value) × Expression correlation*. TF-TF links in the GRN were inferred based on individual motif matches. GRNs were visualized using Cytoscape, v3.10.3^78^.

#### AlphaFold

Protein sequences of 43 *P. tremula* and 22 *P. abies* transcription factors (master regulators from the regulatory network in Fig. 6) were modeled individually using AlphaFold3 (DeepMind/Isomorphic Labs; database release 2025-04-15)^51^. This was used as a control to confirm that models of comparable quality could be obtained for both species, excluding e.g., low multiple sequence alignment quality as a possible confounding factor for downstream protein–protein interaction prediction (Supplementary Fig. 10A). We then used AlphaFold3 to predict models for all heterodimeric transcription factor pairs within species (excluding homodimers) for *P. tremula* and *P. abies*, including Arabidopsis as a control. Model quality was assessed using the *Ranking score* reported directly by AlphaFold3, which combines predicted interface accuracy (ipTM), overall structural accuracy (pTM), disorder fraction, and clash penalties. We found that more complexes had scores indicative of protein-protein interactions in Arabidopsis and *P. tremula* than in *P. abies* (Supplementary Fig. 10B, Supplementary Data 6).

The regulatory networks (Fig. 6) predicted NST as one of the top regulators of SCW Cellulose synthase A genes (CesAs) in *P. tremula*, but not in *P. abies*. Instead, MYB83 was predicted as the top regulator of SCW CesAs in *P. abies*. To test these predictions, AlphaFold3 was used to predict TF-DNA complexes between NST and its predicted DNA binding sites near SCW CesAs. As a positive control in *P. abies*, we also predicted complexes between MYB83 and its binding sites. Specifically, AlphaFold3 was used to model seven double-stranded DNA motifs (5′–3′: ATCCACGCAATGT, ATATACGCAAGCC, GTACAAGCAAAGT, GTACACGCTTTCT, ACTCACGCAATGT, CTATACGCATTCA, CTACAAGCAATAG) in complex with Potra2n2c4785 (NST in *P. tremula*) and PA_chr11_G001721 (NST in *P. abies*). In addition, four motifs (5′–3′: CACCAACA, CACCAACT, GTTTGGTA, GTTAGGTA) were modeled in complex with PA_chr09_G004772 (MYB83 in *P. abies*). The ranking score was used to assess TF–DNA interaction quality, confirming the predicted regulatory differences between species (Supplementary Fig. 10C, Supplementary Data 6).

### Xyloglucan enzymatic fingerprinting

Xyloglucan fingerprinting was performed as described previously^79^. Alcohol insoluble residues prepared from the in vitro-grown whole plant *P. tremula* and from the embryonic *P. abies* tissues were digested with 5 U/mg dry weight of xyloglucanase (Megazyme) in 50 mM ammonium acetate buffer (pH 5) at 37 °C for 18 h. Supernatants were collected after centrifugation (13,000 rpm, 10 min), and 10 μL was injected for LC–MS analysis using an UltiMate 3000 RS HPLC system (Thermo Fisher Scientific) coupled to an Impact II UHR-QqTOF mass spectrometer (Bruker Daltonics) equipped with an electrospray ionization (ESI) source in negative mode (capillary voltage 4000 V; nebulizer 40 psi; 556 dry gas 8 L/min; 180 °C). Chromatographic separation was performed on an ACQUITY UPLC Protein BEH SEC column (125 Å, 1.7 μm, 4.6 × 300 mm; Waters) with a guard column, using 50 mM ammonium formate and 0.1% formic acid at 0.4 mL/min and 40 °C. Data were processed in Compass 1.8 (Bruker Daltonics).

### Reporting summary

Further information on research design is available in the Nature Portfolio Reporting Summary linked to this article.

## Supplementary information

Supplementary Information

Description of Additional Supplementary Files

Supplementary Data 1

Supplementary Data 2

Supplementary Data 3

Supplementary Data 4

Supplementary Data 5

Supplementary Data 6

Reporting Summary

Transparent Peer Review file

## Data Availability

The RNA-seq data generated in this study have been deposited in the European Nucleotide Archive under accession codes ERP016242 (*Populus tremula*, https://www.ebi.ac.uk/ena/browser/view/ERP016242), ERP160851 (*Betula pendula*, https://www.ebi.ac.uk/ena/browser/view/ERP160851), ERP161000 (*Prunus avium*, https://www.ebi.ac.uk/ena/browser/view/ERP161000), ERP161001 (*Picea abies*, https://www.ebi.ac.uk/ena/browser/view/ERP161001), ERP161017 (*Pinus sylvestris*, https://www.ebi.ac.uk/ena/browser/view/ERP161017) and ERP161018 (*Pinus contorta*, https://www.ebi.ac.uk/ena/browser/view/ERP161018). The ATAC-seq data generated in this study have been deposited in the European Nucleotide Archive under accession code ERP187628 (https://www.ebi.ac.uk/ena/browser/view/ERP187628).

## Code availability

All code generating the results and figures in this article is available at https://github.com/ellendim/EVOTREE and archived, together with the input files required to run it, at Zenodo (https://doi.org/10.5281/zenodo.21025760).
