# Supplementary material for: Comparative regulomics of wood formation across dicot and conifer trees
Source: Nat Commun. 2026 Jul 22;17:8916. doi: 10.1038/s41467-026-75624-2 (PMC13503837; doi:10.1038/s41467-026-75624-2)
Supplement: Supplementary file 1 [file 41467_2026_75624_MOESM10_ESM.pdf]

nature portfolio

Peer Review File

Comparative regulomics of wood formation across dicot and conifer trees

Corresponding Author: Professor Torgeir Hvidsten

This file contains all reviewer reports in order by version, followed by all author rebuttals in order by version.

Version 0:

Reviewer comments:

Reviewer #1

(Remarks to the Author)
This study, submitted by Rodriguez and colleagues, provides a comprehensive cross-species resource to investigate the evolution and regulation of wood formation. By integrating high-spatial-resolution transcriptomic data from an impressive set of six tree species, the authors follow a rather unique approach in comparative wood biology. I believe that the orthology-aware co-expression network analysis is a particular strength, allowing, as the authors also state, the identification of both conserved and lineage-specific gene expression programs to be investigated in subsequent studies. The addition of chromatin accessibility and transcription factor motif analyses adds analytical depth, allowing, for example, the establishment of regulatory networks underlying xylem differentiation and secondary cell wall formation. Overall, this work therefore represents a valuable and technically impressive contribution to the fields of tree biology and evo-devo research. On the downside, experimental validation of exemplary predictions made in this study is not provided leaving the relevance of the analyses somewhat unclear. For example, and although this may be tedious work, deduced expression patterns are not confirmed for a few cases in the different species. Also, can predictions on differences in cell wall composition be confirmed by analysing these compositions in the gymno- and angiosperm lineages? Likewise, expression networks (Fig. 6) are established based on transcriptome analyses but not validated by modulating expression activities of some network members. Yes, this is hardly possible when using tree species but maybe the authors find ways to validate these predictions indirectly by using e.g. Arabidopsis? Beyond this major gap, explanations of analytical details and data description could be improved to allow better access to the study by the average reader.
My specific comments are the following:
1. Page 2, last paragraph: Groover et al. is not a comparative genomics study. Please revise.
2. Results, first paragraph: Please move these two sentences to the introduction.
3. Fig. S1, Tables S1 and 2 (but others as well): I had quite some difficulties to follow the experimental setup due to inconsistencies in the nomenclature and missing explanations. Why are there 5 sections shown in Fig. S1 but six species analysed? Sometimes the common name of species is used in figures, tables and texts, sometime the botanical name. This makes comparison and general access to the study very tedious. Please use the same names and abbreviations throughout the manuscript. Then I do not always understand how do the different Tables (e.g. S1+S2) correspond to each other? Where is for example Aspen in S1? How do the letters in Table S2 correspond to samples indicated in Table S1? The authors should go through their manuscript and look at their data presentation with the eye of a non-familiar reader.
4. The central point of the study is cross-species comparison of transcriptomes in different wood-associated tissues. It is, however, not clear to me how the different sample types from different trees can be faithfully aligned (and categorized as in Table S1). Although major tissues can certainly be identified, how to do that for transition stages? This is also because it is stated later that “sample series were not directly comparable among species due to anatomical and growth differences”. As this will probably strongly affect comparisons between species, the classification procedure should be better explained and, if possible, supported by related images/data.
5. Page 4, line 3: six transcriptomes -> six species-specific transcriptomes
6. Fig. S2: What do the coloured bars on the left indicate? The numbers at the bottom of the graphs are far too small.
7. Page 4, line 12: There are no anatomical analyses shown in Table S1. Please add those (see also my comment toward sample alignment above).
8. Page 5, line 7: Please add a sentence describing the criteria for defining orthologs here.
9. Page 5, line 8: Please justify the selection of those 27 species and indicate which species those were.
10. Page 5: “… in contrast to the original definition of an expressolog” Could you indicate what the original definition of an expressolog really is?

11. Page 5: Is the higher numbers of within- than between-lineage co-expressologs not only or partly because there are more orthologs within- than between-lineages in the first place? I guess this depends on which orthogroups you use for the analysis (see Fig. 2A). Thus, I think it is important to mention which orthogroups were included. I suggest adding a comment to this point.
12. Fig. 2C: I think I cannot follow the statement "i.e. yellow squares of steady state expression in Fig. 2C, of which the SCW stage was the most apparent, separated by transitions/reprogramming events". Please, explain better and also explain what the axes in each square of Fig. 2C indicate.
13. Fig. 3 C, D: What do the different lines in Fig. 3C and D indicate?

(Remarks on code availability)

Reviewer #2

(Remarks to the Author)
This manuscript provides a broadly useful multi-species resource on wood formation, but it overstates its novelty and draws evolutionary and functional conclusions that are not fully supported by the sampling design, analytical methods, or lack of experimental validation.

Major：
1. Overstatement of novelty and insufficient distinction from existing resources
The manuscript repeatedly claims to deliver the "first high-spatial-resolution evo-devo resource for wood" (e.g., Abstract). However, previously published datasets such as AspWood and NorWood already provide high-resolution developmental transcriptomes for Populus and Picea (AspWood: High-Spatial-Resolution Transcriptome Profiles Reveal Uncharacterized Modularity of Wood Formation in Populus tremula ，doi.org/10.1105/tpc.17.00153；NorWood: a gene expression resource for evo-devo studies of conifer wood development，doi.org/10.1111/nph.14458. The present study extends species breadth but does not constitute a conceptual breakthrough.

2. Sampling design does not support evolutionary inference
Sampling occurred in different years, locations, and environments, and each species uses a single genotype with limited biological replication (Page 17, Paragraph 1). Despite this, the Results frequently interpret expression patterns as evidence of "250 Myr of evolutionary conservation or divergence" (e.g., Page 7, Paragraph 2).

3. Methodological descriptions lack clarity; key thresholds lack justification
1) Co-expressolog definition is insufficiently justified and explained
The manuscript introduces "co-expressologs" as conserved co-expression relationships across species (Page 5, Paragraph 3). However, it is unclear why this metric is biologically superior to comparing direct expression similarity or orthogroup-level clustering.
Methods provide minimal details (Page 19, Last paragraph), and no benchmarking is shown.

2) Network and TF–motif–module thresholds lack justification
The gene network and module inference rely on arbitrary cutoffs without statistical or biological justification. The manuscript also lacks sensitivity analysis showing whether results are robust to different thresholds.

3) AlphaFold3 predictions used without controls
TF–TF and TF–DNA binding predictions (introduced Page 15, Paragraph 1) are used to interpret cross-species interaction gains and losses. There is no negative/positive control, no scoring criteria for confidence, and no validation.

4. Multiple biological conclusions rely solely on correlation and are not experimentally validated
1） The interpretation of H3.1 and H3.3 spatial separation (Page 7, 2nd paragraph) as reflecting replication-dependent vs. reprogramming function exceeds what expression data alone can support.
2） Functional implications of the POK2 "second peak" in conifers (Page 9, 2nd paragraph) are speculative.

5. ATAC-seq performed in only two species but interpreted for all six
ATAC-seq is conducted only for Populus tremula and Picea abies (Page 14, Paragraph 3). Nevertheless, regulatory conclusions are generalized to all six species.

Minor Comments
1. Language overly absolute or subjective
Statements such as "poorly understood" and "first high-resolution evo-devo resource" appear in the introduction, overstating novelty.

2. Figures and textual descriptions mismatched; key results insufficiently visualized
1） Fig. 2C lacks clear developmental-region labels.
2） Fig. 3B's "unique cliques" appear highly curated, but selection criteria are not described.
3） TBL gene expression (e.g., TBL29, TBL37; Page 12, Paragraphs 2–4) does not fully align with claims regarding conserved acetylation functions.

3. Key QC and preprocessing steps not fully documented
Criteria for removing outliers (mentioned broadly Page 19, 2nd paragraph) lack explicit thresholds.
ATAC-seq QC metrics, replicate numbers, and peak counts are missing (Methods around Page 14).

4. The consistency between sample replicates is not demonstrated.
Across the different data types (RNA-seq, ATAC-seq, etc.), the number of biological replicates per species and per condition is not clearly summarized, and the reproducibility between replicates is not shown. The manuscript would benefit from an explicit description of replicate numbers in the main text or a summary table, and from quantitative assessments of replicate consistency (e.g. correlation matrices, PCA clustering by replicate, basic QC metrics), so that the robustness of the downstream network and comparative analyses can be properly evaluated.

5. Data and code availability insufficiently documented
PlantGenIE usage is not described.

(Remarks on code availability)

Reviewer #3

(Remarks to the Author)
This manuscript presents an ambitious and valuable comparative evo-devo study of wood formation, combining spatially resolved transcriptomics across six tree species (three dicots and three conifers) with chromatin accessibility and transcription factor motif analyses. The dataset spans over 250 million years of evolutionary divergence and represents an important community resource. The work has clear potential impact for plant biology, but several methodological and interpretational aspects require clarification and improvement before publication.

Major Comments

1. Figure labeling and interpretability: Several figures (Fig. 1C, Fig. 4B-D, Fig. 5A-B, Fig. 6A-B) would benefit from adding labeling of x-axes to indicate tissue types to improving readability for a general audience, for example, by including color bars similar to Fig. 3B.

2. Phylogenetic signal of expression conservation: The statement on Page 5 "corresponding samples within dicots and conifers showing much higher correlation (~0.75) than samples compared between the two lineages (~0.55; Fig. S3B)" is not supported by the current figure Fig. S3B. The bimodal distribution is apparent, but the figure lacks annotation to distinguish "corresponding samples". Please provide evidence or additional visualization (e.g., sample grouping, labeling, or correlation heatmap) to substantiate this claim.

3. Clarification of Fig. 2C and related conclusions: It is unclear what Fig. 2C is plotting: it is not explained whether rows/columns represent genes or samples, nor how "transition" or "steady-state" phases were defined. And it is not clear how Fig. 2C leads to this conclusion "our analyses also revealed the same major stages of wood formation, and the corresponding reprogramming events, seen in cluster analysis of genes (Fig. S2) (i.e. yellow squares of steady state expression in Fig. 2C, of which the SCW stage was the most apparent, separated by transitions/reprogramming events)."

4. Integration of ATAC-seq and transcriptome data: I am not an expert of wood biology, so it will help if the authors can specify which tissue(s) the ATAC-seq data were derived from relative to the spatially resolved transcriptomes. Because biological meaning of the network in Fig. 6C depends critically on this correspondence, the rationale for integrating motif enrichment in accessible chromatin from these ATAC-seq samples with transcriptome-based co-expression modules needs to be explicitly justified.

5. Claim of purifying selection (Page 14, last paragraph): The statement that "strong purifying selection" is indicated by association of TFs (VNDs, MYB46/83) with SCW modules is not supported. Motif conservation and inferred regulatory association do not constitute evidence of purifying selection. Moreover, motif presence in accessible regions does not necessarily indicate TF binding, and binding sites may have shifted (i.e., a TF can bind to promoters of orthologous genes in two species but the actual binding site might have moved). A more accurate statement would be that these genes "share conserved network targets".
6. AlphaFold analysis and interpretation: It is unclear whether AlphaFold predictions were run multiple times with different random seeds because the predicted structures could be different from different random seeds. The y-axis label ("model quality") in Fig. S7 should be defined, and the connection between predicted interaction quality and the conclusion that "master regulators are predicted to frequently interact" needs clarification. Furthermore, were these predicted complexes linked to co-expression modules? Currently, this analysis does not convincingly support the conclusion about regulatory complexity.

7. Regulatory network architecture (Page 15): The claim that the P. tremula network "adopts a layered architecture with feed-forward loops" should be directly supported with a figure or network motif analysis. Similarly, the statement that the P. abies network is "flatter" requires supporting evidence, e.g., is the lack of VND to MYB edges due to lack of accessible regions in the MYB promoters or the lack of VND motifs? Displaying the ATAC-seq profiles and motif presence for these two species

side-by-side could help support these claims.

8. Module detection and network inference methods: For transcriptional module, why was a custom clustering method used instead of many established approaches such as WGCNA? It is fine to use a custom method, but he manuscript should show that the genes in the resulting modules are indeed co-expressed, and the average profiles for the module accurately represent the expression patterns of genes in the module. This is essential because module-level expression patterns were used for GRN inference and TF assignment, directly influencing downstream conclusions about the interspecies network differences.

9. ATAC-seq data quality and reporting: Quality control metrics are missing for the ATAC-seq datasets. Please include information such as read counts, unique mapping rate, fragment length distribution, and TSS enrichment scores see Yan et al Genome Biol 2020 https://doi.org/10.1186/s13059-020-1929-3, Ou et al BMC Genomics 2018 https://doi.org/10.1186/s12864-018-4559-3). The number of called peaks and representative genome browser tracks for key loci would greatly strengthen confidence in the data integration and conclusions.

10. It seems the public website that the author cited (plantgenie.org) only provides visualization of the data. It is unclear from the manuscript whether the raw or processed transcriptome and ATAC-seq data have been deposited in a public repository.

(Remarks on code availability)

Version 1:

Reviewer comments:

Reviewer #1

(Remarks to the Author)
The authors have convincingly addressed all my concerns. As I mentioned before, this work certainly represents a valuable and technically impressive contribution to the fields of tree biology and evo-devo research and I appreciate the author's achievements.

(Remarks on code availability)

Reviewer #2

(Remarks to the Author)
I appreciate the authors' revisions and the added LCMS validation, but I remain concerned that the main conclusions are still stronger than the evidence supports.

1. The manuscript makes broad claims about dicot–conifer regulatory divergence over ~250 Myr, but the actual design is much narrower: six species total, only three per lineage, one genotype per species, and only 3–4 clonal replicate trees per genotype. These replicates address within-genotype technical/biological reproducibility, but they do not test genotype-level, population-level, species-level, or environmental variation. Moreover, the species were sampled across different sites and years. Thus, the study effectively compares six sampled genotypes under non-equivalent conditions, yet interprets the results as lineage-level evolutionary conservation or rewiring.
2. The regulatory-network conclusions are even more underpowered. ATAC-seq was performed in only 2 of the 6 species — P. tremula and P. abies, i.e. one species per lineage — and from bulk xylem scrapings rather than spatially matched samples. Therefore, the key GRN conclusion is based on a 1-vs-1 comparison, not a replicated dicot-versus-conifer design. A two-species, bulk-accessibility dataset cannot establish a general lineage-level difference in regulatory architecture.
3. The analytical conclusions also depend on several user-defined thresholds: the top 3% of co-expression links, FDR < 0.05 motif enrichment, Pearson correlation > 0.7 for TF–module assignment, and custom module definitions. These choices are central to the reported cliques and GRN differences, yet the manuscript does not show that the main biological conclusions remain stable across alternative reasonable thresholds or standard methods.
4. Finally, the manuscript contains very limited functional validation. The added LCMS experiment validates one cell-wall prediction in two species, but the main claims about NST/VND/MYB rewiring, H3 variant function, POK2 expression, and GRN architecture remain based on co-expression, motif enrichment, bulk chromatin accessibility, and AlphaFold predictions. There is no direct perturbation, no TF binding validation, and no functional test of the inferred regulatory hierarchy.

The work may be useful as a descriptive transcriptomic resource, but it is not convincing as evidence for lineage-level regulatory rewiring. The claims should be substantially narrowed.

(Remarks on code availability)

Reviewer #3

(Remarks to the Author)
I thank the authors for their careful revision. Most of my previous comments have been adequately addressed, and I have only two remaining minor comments.

Round 1 review comment #6: AlphaFold analysis

The authors' response to my previous comment is satisfactory. I only ask that they explicitly specify the source of the reported "ranking score" in the Methods or Fig. S10 legend. I assume this refers to the ranking score output directly from AlphaFold3, but this should be stated clearly.

Round 1 review comment #9: ATAC-seq quality metrics and interpretation

I appreciate that the authors have added ATAC-seq QC metrics, including fragment size distributions, TSS enrichment scores, peak counts, and genome browser tracks. These additions are helpful and improve the reporting of the dataset.

However, the newly provided QC metrics suggest some limitations in ATAC-seq data quality. Specifically, the fragment length distributions appear to lack a clear mono-nucleosome-bound fragment peak around ~200 bp, which is typically expected in ATAC-seq data (Fig 1B in Yan et al., Genome Biology 2020, https://doi.org/10.1186/s13059-020-1929-3; Troubleshooting 3 in Marand et al., 2021 https://doi.org/10.1016/j.xpro.2021.100737). In addition, the reported TSS enrichment scores appear relatively low. Although mammalian ENCODE standards (https://www.encodeproject.org/atac-seq/#standards) may not translate directly to plant tissues, TSS enrichment scores above ~5–6 are generally expected for high-quality ATAC-seq data.

To help readers assess the data quality more fully, I recommend that the authors provide full TSS enrichment profiles, rather than only summary scores. A plot similar to Yan et al. 2020, Fig. 1C, would be useful for showing whether the expected enrichment around transcription start sites is present.

I do not view this issue as a barrier to publication, given the overall strengths of the manuscript and the importance of the spatial transcriptomic data. However, because ATAC-seq in these plant samples is understandably challenging, I recommend that the authors modestly temper the language around the ATAC-seq-based GRN conclusions and present these results as supportive rather than definitive evidence. For example, I suggest replacing definitive language such as "The resulting robust TF-module networks", "the regulatory network," "top layer master regulators," "the structure of the P. tremula regulatory network", "our analysis paints a picture of a more streamlined network" with more cautious phasing such as "candidate TF-module networks, "candidate regulatory network", "proposed top layer master regulators, "the structure of the candidate P. tremula regulatory network" , and "our analysis is consistent with a more streamlined network", etc.

(Remarks on code availability)

Open Access This Peer Review File is licensed under a Creative Commons Attribution 4.0 International License, which permits use, sharing, adaptation, distribution and reproduction in any medium or format, as long as you give appropriate credit to the original author(s) and the source, provide a link to the Creative Commons license, and indicate if changes were made.
In cases where reviewers are anonymous, credit should be given to 'Anonymous Referee' and the source.
The images or other third party material in this Peer Review File are included in the article's Creative Commons license, unless indicated otherwise in a credit line to the material. If material is not included in the article's Creative Commons license and your intended use is not permitted by statutory regulation or exceeds the permitted use, you will need to obtain permission directly from the copyright holder.
To view a copy of this license, visit https://creativecommons.org/licenses/by/4.0/

Reviewer #1

This study, submitted by Rodriguez and colleagues, provides a comprehensive cross-species resource to investigate the evolution and regulation of wood formation. By integrating high-spatial-resolution transcriptomic data from an impressive set of six tree species, the authors follow a rather unique approach in comparative wood biology. I believe that the orthology-aware co-expression network analysis is a particular strength, allowing, as the authors also state, the identification of both conserved and lineage-specific gene expression programs to be investigated in subsequent studies. The addition of chromatin accessibility and transcription factor motif analyses adds analytical depth, allowing, for example, the establishment of regulatory networks underlying xylem differentiation and secondary cell wall formation. Overall, this work therefore represents a valuable and technically impressive contribution to the fields of tree biology and evo-devo research.

Answer: We thank the reviewer for the positive assessment of our work and for the constructive feedback. A point-by-point response can be found below.

On the downside, experimental validation of exemplary predictions made in this study is not provided leaving the relevance of the analyses somewhat unclear. For example, and although this may be tedious work, deduced expression patterns are not confirmed for a few cases in the different species. Also, can predictions on differences in cell wall composition be confirmed by analysing these compositions in the gymno- and angiosperm lineages? Likewise, expression networks (Fig. 6) are established based on transcriptome analyses but not validated by modulating expression activities of some network members. Yes, this is hardly possible when using tree species but maybe the authors find ways to validate these predictions indirectly by using e.g. Arabidopsis? Beyond this major gap, explanations of analytical details and data description could be improved to allow better access to the study by the average reader.

Answer: We appreciate the reviewer's suggestions for experimental validation.

In response to the reviewer's specific suggestion regarding cell wall composition, we have now performed liquid chromatography–mass spectrometry (LCMS) analysis of xyloglucan oligosaccharides from *P. tremula* and *P. abies* primary cell wall material. Our comparative transcriptomic analysis predicted that xyloglucan acetylation is dicot-specific, based on the absence of xyloglucan-targeting TBL orthologs and their expression in conifers. The LCMS data confirm this prediction: acetylated xyloglucan fragments were abundant in *P. tremula* but nearly absent in *P. abies* (Fig. 5C). This represents, to our knowledge, the first demonstration that xyloglucan acetylation

differs between dicots and conifers, and provides direct experimental validation of a key prediction from our comparative analysis.

We appreciate the reviewer's suggestion regarding qPCR confirmation of a subset of differentially expressed genes. However, we have elected to rely on the robust statistical framework of our RNA-Seq data rather than secondary qPCR validation. Modern transcriptomic standards suggest that when an experiment is well-replicated and sequenced to sufficient depth, the dynamic range and sensitivity of RNA-Seq surpass those of qPCR (Coenye, 2021; Ma et al., 2019). Furthermore, high-powered studies have demonstrated that biological replication is the primary driver of data reliability, and a well-replicated RNA-Seq experiment (such as the one presented here, see Fig. S2) provides more statistical confidence than a small subset of technical validations (Schurch et al., 2016). Finally, qPCR validation introduces its own technical biases, specifically regarding the selection and stability of reference genes, which can often be less reliable than the global normalization methods used in RNA-Seq (Eisenberg & Levanon, 2013). Given the high correlation between these platforms in well-powered studies, we believe the current dataset provides a more accurate and comprehensive representation of the transcriptome than a subset of targeted assays.

References
Coenye, T. (2021). Do we really need qPCR to "validate" RNA-Seq data? Bioengineered, 12(1), 1836–1837. DOI: 10.1080/21505594.2021.1927544

Eisenberg, E., & Levanon, E. Y. (2013). Human housekeeping genes, revisited. Trends in Genetics, 29(10), 569–574. DOI: 10.1016/j.tig.2013.05.010

Ma, Z., et al. (2019). The RNA-Seq validation myth. bioRxiv. DOI: 10.1101/562306

Schurch, N. J., et al. (2016). How many biological replicates are needed in an RNA-seq experiment and which differential expression tool should you use? RNA, 22(6), 839–851. DOI: 10.1261/rna.053157.115

Regarding the regulatory networks, the central finding of our study is precisely the comparison to Arabidopsis (which is also an indirect validation of our network using literature): we show that the aspen network mirrors the characterized Arabidopsis architecture (three layers), while the spruce network is notably different. Functional validation in conifers would require years of experimental work and is beyond the scope of this comparative genomics study. Our predictions are supported by chromatin accessibility data, motif enrichment analysis, co-expression patterns, and AlphaFold-predicted protein interactions, providing multiple lines of computational evidence for the inferred regulatory differences.

Finally, we have worked on the text more generally, and hope that the reviewer finds the new revision more accessible.

My specific comments are the following:
1. Page 2, last paragraph: Groover et al. is not a comparative genomics study.

Answer: We agree and have rewritten the start of this paragraph.

2. Results, first paragraph: Please move these two sentences to the introduction.

Answer: We have moved these sentences to the introduction as suggested and removed redundancy.

3. Fig. S1, Tables S1 and 2 (but others as well): I had quite some difficulties to follow the experimental setup due to inconsistencies in the nomenclature and missing explanations. Why are there 5 sections shown in Fig. S1 but six species analysed? Sometimes the common name of species is used in figures, tables and texts, sometime the botanical name. This makes comparison and general access to the study very tedious. Please use the same names and abbreviations throughout the manuscript. Then I do not always understand how do the different Tables (e.g. S1+S2) correspond to each other? Where is for example Aspen in S1? How do the letters in Table S2 correspond to samples indicated in Table S1? The authors should go through their manuscript and look at their data presentation with the eye of a non-familiar reader.

Answer: We apologize for the inconsistencies in nomenclature. We have now standardized the use of Latin names also in the supplementary materials, where common names were used in several places. Regarding aspen (*Populus tremula*), the sample table and cryosectioning image were not included in Table S1/Fig S1 because these data were previously published in the AspWood study (Sundell et al. 2017). However, we agree this creates confusion and have now added the aspen sample information to Table S1 for completeness. We have cleaned up Table S1 and added a summary table in the legend-tab with species names (both Latin and common), number of replicates, and number of samples. We have also added a legend-tab to Table S2 where we explain the sample naming.

4. The central point of the study is cross-species comparison of transcriptomes in different wood-associated tissues. It is, however, not clear to me how the different sample types from different trees can be faithfully aligned (and categorized as in Table S1). Although major tissues can certainly be identified, how to do that for transition stages? This is also because it is stated later that “sample series were not directly comparable among species due to anatomical and growth differences”. As this will probably strongly affect comparisons between species, the classification

procedure should be better explained and, if possible, supported by related images/data.

Answer:

We appreciate the opportunity to clarify this important methodological point.

The tissue annotations shown in Table S1 are based on anatomical observations during cryosectioning. The major developmental transitions within each species (phloem, expansion, SCW formation, maturation) were identified through hierarchical clustering of gene expression data (Fig. S2) and these anatomical observations (Table S1), and adjusted using marker gene expression profiles (Fig. 1C). While hierarchical clustering provides an objective starting point, we found that the exact placement of samples into four clusters can vary based on which genes are included (e.g. cutoffs for what constitutes an expressed gene). The integrated approach that we used combines objective clustering with biological validation. The final transitions used throughout the paper are shown as dotted lines in Fig. 1C.

Importantly, our cross-species comparisons do not rely on direct sample-to-sample alignment across species, and thus not on the placement of transitions. In fact, the transitions are only used to interpret the expression profiles. Instead, we use co-expression network analysis within each species and then compare these networks across species using the co-expressolog approach (Fig. 2B). This method specifically accounts for the fact that sample series are not directly comparable due to anatomical and growth differences between species.

We have revised the text to better explain these methodological distinctions and clarify that cross-species comparisons are based on conserved co-expression relationships rather than direct sample alignment.

5. Page 4, line 3: six transcriptomes -> six species-specific transcriptomes

Answer: We have revised the text.

6. Fig. S2: What do the coloured bars on the left indicate? The numbers at the bottom of the graphs are far too small.

Answer: We have removed the gene cluster color bars from Fig. S2 as these clusters are not used in downstream analyses. We have also increased the font size of the labels and improved the overall figure quality for better readability.

7. Page 4, line 12: There are no anatomical analyses shown in Table S1. Please add those (see also my comment toward sample alignment above).

Answer: The columns labeled 'Tissue' in Table S1 contain the anatomical observations made during cryosectioning. We have clarified this in the table legend.

8. Page 5, line 7: Please add a sentence describing the criteria for defining orthologs here.
9. Page 5, line 8: Please justify the selection of those 27 species and indicate which species those were.

Answer: We have added a reference to the Methods section where we describe how we identified orthologs (OrthoFinder). We have also expanded the methodological details there, including a justification for the selected species and a complete list of all 27 species used in the analysis.

10. Page 5: "… in contrast to the original definition of an expressolog" Could you indicate what the original definition of an expressolog really is?

Answer: We have revised the text to clarify that the original definition of expressologs relied on direct expression correlation across matching samples between two species, whereas our co-expressologs are based on comparing co-expression networks and thus do not require exact sample matching.

11. Page 5: Is the higher numbers of within- than between-lineage co-expressologs not only or partly because there are more orthologs within- than between-lineages in the first place? I guess this depends on which orthogroups you use for the analysis (see Fig. 2A). Thus, I think it is important to mention which orthogroups were included. I suggest adding a comment to this point.

Answer: Fig. 2A shows both all orthogroups (upper right corner) and the subset of orthogroups with expressed genes in wood. We have revised the figure text to make this clearer. Co-expressologs require expressed genes, so the numbers in Fig. S3A represent a subset of the expressed orthogroups.

The reviewer is correct that the number of orthogroups with co-expressologs (Fig. S3A) can be affected by the total number of orthogroups, which is larger within lineages than between lineages. We have now also added the total number of orthologs compared, and colored by the fraction of orthogroups with co-expressologs in Fig. S3A. The text is revised accordingly.

12. Fig. 2C: I think I cannot follow the statement "i.e. yellow squares of steady state expression in Fig. 2C, of which the SCW stage was the most apparent, separated by transitions/reprogramming events". Please, explain better and also explain what the axes in each square of Fig. 2C indicate.

Answer: We agree that this explanation was unclear. We have revised the text, figure legend, and figure to better explain the patterns observed in Fig. 2C, including clarification of what the axes represent in each heatmap and color bars marking the developmental stages from phloem to cell death.

13. Fig. 3 C, D: What do the different lines in Fig. 3C and D indicate?

Answer: The dotted lines indicate the developmental transitions between wood formation stages discussed above. This was only described in the Fig. 1C legend ('Dotted vertical lines mark, from left to right, the middle of the cambium, the end of the expansion zone and the end of the SCW zone'), which correspond to the color bars shown in the heatmap in Fig 3B. We have made these lines more visible and added consistent descriptions of what they represent in all figure legends throughout the manuscript.

Reviewer #2:

This manuscript provides a broadly useful multi-species resource on wood formation, but it overstates its novelty and draws evolutionary and functional conclusions that are not fully supported by the sampling design, analytical methods, or lack of experimental validation.

Answer: We thank the reviewer for recognizing the value of our resource and for the detailed feedback. We provide point-by-point responses below addressing the concerns raised.

Major:
1. Overstatement of novelty and insufficient distinction from existing resources
The manuscript repeatedly claims to deliver the “first high-spatial-resolution evo-devo resource for wood” (e.g., Abstract). However, previously published datasets such as AspWood and NorWood already provide high-resolution developmental transcriptomes for Populus and Picea (AspWood: High-Spatial-Resolution Transcriptome Profiles Reveal Uncharacterized Modularity of Wood Formation in Populus tremula , doi.org/10.1105/tpc.17.00153；NorWood: a gene expression resource for evo-devo studies of conifer wood development, doi.org/10.1111/nph.14458. The present study extends species breadth but does not constitute a conceptual breakthrough.

Answer: We apologize if our wording suggested we were downplaying previous work. The AspWood dataset (Sundell et al. 2017) for *Populus tremula* is indeed included in our study - we now state this explicitly at the beginning of the Results section, in addition to the existing citation. For *Picea abies*, we generated new data because the NorWood dataset (Jokipii-Lukkari et al. 2017) had limitations including lower data quality and lack of phloem samples.

Regardless, we would argue that two species do not constitute an evo-devo resource. With only one dicot and one conifer, it is not possible to distinguish lineage-level divergence from species-specific noise. Only with multiple species within each lineage can we reliably identify genes that are, for example, conserved within dicots and within conifers but diverged between the two lineages (e.g., Fig. 4). The analytical framework for comparing six species using co-expressologs and identifying conserved, lineage-specific, and diverged cliques has also not been presented before.

We have carefully revised our wording throughout the manuscript to more accurately describe the novelty and avoid overstating our contributions relative to prior work.

2. Sampling design does not support evolutionary inference
Sampling occurred in different years, locations, and environments, and each species uses a single genotype with limited biological replication (Page 17, Paragraph 1). Despite this, the Results frequently interpret expression patterns as evidence of "250 Myr of evolutionary conservation or divergence" (e.g., Page 7, Paragraph 2).

Answer: We appreciate the opportunity to clarify our sampling design and its implications for evolutionary inference.

Finding suitable material for all six species at a single location was not possible, as our study requires mature trees (wood formation is not comparable in seedlings), which cannot be planted and sampled within a practical timeframe. Moreover, different tree species have distinct environmental preferences, making a common-garden experiment impractical and potentially undesirable.

Our use of clonal replicates (3-4 per species) was a deliberate choice to minimize noise from genotypic variation while maintaining biological replication. Fig. S2 demonstrates high reproducibility across replicates within each species, indicating that environmental variation had limited impact on the core developmental expression patterns captured in our study.

Regarding potential impacts of environmental variation on our conclusions: (1) False positives from environmental effects creating spurious co-expression conservation across six (or even three) species are extremely unlikely. (2) False negatives—where environmental variation masks true conservation—are possible, but our conclusions are based on positive findings of conservation observed consistently across multiple species (either all six, or three species within a lineage).

We have revised the text to better explain our sampling rationale and clarify that our evolutionary inferences are conservative, based on consistently observed patterns across species rather than absence of patterns.

3. Methodological descriptions lack clarity; key thresholds lack justification

1) Co-expressolog definition is insufficiently justified and explained

The manuscript introduces “co-expressologs” as conserved co-expression relationships across species (Page 5, Paragraph 3). However, it is unclear why this metric is biologically superior to comparing direct expression similarity or orthogroup-level clustering.

Methods provide minimal details (Page 19, Last paragraph), and no benchmarking is shown.

Answer: Due to anatomical differences between species and growth differences between individuals, it is not possible to obtain samples that are directly comparable on a sample-by-sample basis across species—a point we make in the manuscript. This is not a method-limitation - it is biology. While the reviewer is correct that methods exist for comparing expression at the orthogroup level, our goal was to identify the most likely 'functional orthologs', as discussed specifically in the histone example (Fig. 3CD). This is particularly important in our dataset, where orthogroups often contain multiple gene copies per species (especially in conifers due to extensive local duplications). The combination of our analytical framework with our multi-species wood expression dataset represents the key contribution of our study, and we are not aware of similar studies and methods.

Regarding thresholds and method choices, co-expression networks were inferred using Pearson correlation with mutual rank normalization, a combination previously benchmarked in the PlaNet method (Mutwil et al. 2011, cited in Methods). We agree with the reviewer that the choice of the top 3% most strongly co-expressed links was not sufficiently justified. We have now added a supplementary figure (Fig. S11) showing the number of orthogroups with co-expressologs as a function of the network density threshold. While the number of orthogroups with co-expressologs increases with threshold, there is a saturation effect: most new co-expressologs are gained between 1% and 3%, with diminishing returns above 3%. Moreover, higher thresholds imply that co-expressologs are inferred from increasingly larger neighbourhoods with weaker correlations and higher statistical power, leading to co-expressologs with progressively more divergent expression profiles (visual inspection). The 3% threshold was chosen based on both empirical evidence (saturation of conserved orthogroups) and visual inspection of expression profiles for detected co-expressologs involving well-characterized SCW gene families.

We have expanded the method description in the Methods sections to better explain the rationale and implementation of the co-expressolog approach.

2) Network and TF–motif–module thresholds lack justification

The gene network and module inference rely on arbitrary cutoffs without statistical or biological justification. The manuscript also lacks sensitivity analysis showing whether results are robust to different thresholds.

Answer: The regulatory network construction uses two stringent, statistically justified criteria: (1) the TF's motif must be significantly enriched in the module (FDR-corrected p-value < 0.05), and (2) the TF must show significant expression correlation with the module's average expression profile (FDR-corrected p-value < 0.05 and Pearson correlation > 0.7). Applying a correlation threshold of 0.7 represents an additional effect size filter beyond statistical significance. This is a common practice to ensure biological relevance, and in our case was determined through visual inspection of the data. Importantly, all TFs meeting these criteria are listed in Table S6, and it is evident that known SCW regulators consistently appear at the top of the rankings regardless of the specific threshold chosen, demonstrating the robustness of our findings.

We have revised the Methods section to more clearly describe how correlations between TFs and modules were computed and to better justify our threshold selection.

3) AlphaFold3 predictions used without controls
TF–TF and TF–DNA binding predictions (introduced Page 15, Paragraph 1) are used to interpret cross-species interaction gains and losses. There is no negative/positive control, no scoring criteria for confidence, and no validation.

Answer: We agree that the Methods section lacked sufficient detail on the AlphaFold predictions and have substantially expanded it.

Regarding controls and scoring criteria: our study does in fact include both positive and negative controls, and applies established confidence thresholds, as described in the Fig. S10 legend — though we acknowledge these details should also have been in the Methods section.

Specifically: (1) Individual TF structures were first modeled using AlphaFold3 to confirm that structure prediction quality (assessed by ranking score) was comparable between the two species, ensuring that any differences in predicted interactions are not due to differences in e.g. multiple alignment quality (Fig. S10A). (2) For TF–TF complexes, Arabidopsis was included as a positive control species with well-characterized interactions, and a ranking score threshold of 0.65 was applied — a value both supported by the literature and consistent with a natural threshold in our data (Fig. S10B). (3) For TF–DNA predictions, we included MYB83 binding its predicted sites in spruce as a positive control alongside the NST predictions,

showing that the method can detect TF–DNA interactions in both species when they are expected to occur (Fig. S10C).

To further assess prediction robustness, all AlphaFold3 predictions were run with three independent random seeds, each generating three diffusion samples, for a total of nine predictions per complex. Ranking scores were highly consistent across seeds and samples, confirming that the predictions are not sensitive to stochastic variation in the diffusion process.

We have moved these methodological details, including the control design, confidence thresholds, and reproducibility assessment, into the Methods section of the revised manuscript.

4. Multiple biological conclusions rely solely on correlation and are not experimentally validated
1）The interpretation of H3.1 and H3.3 spatial separation (Page 7, 2nd paragraph) as reflecting replication-dependent vs. reprogramming function exceeds what expression data alone can support.
2）Functional implications of the POK2 “second peak” in conifers (Page 9, 2nd paragraph) are speculative.

Answer: We agree that these interpretations should be clearly framed relative to what expression data can and cannot support.

For H3.1, we have softened the wording to read: "Hence, H3.1 marks the proliferative phase of wood formation, where cambial cells are undergoing mitotic divisions before differentiating into xylem or phloem, in agreement with the expectation that DNA replication is largely restricted to the cambium during wood formation." This makes clear that our expression data are consistent with the known replication-dependent function of H3.1, rather than independently demonstrating it.

For H3.3, we note that the observation itself — constitutive expression across the wood series, in contrast to the cambium-specific expression of H3.1 — is a straightforward empirical finding. The functional interpretation that H3.3 is associated with transcription-independent chromatin remodeling is well established in the literature (Foroozani et al. 2022), and we are simply noting consistency with this known biology.

For POK2, we have revised the text to present the second expression peak as an empirical observation without speculating on its functional significance. The revised text now reads:

"While POK2 showed peak expression in the cell division zone in both lineages, consistent with its known role in cell plate formation and alignment, it also displayed

a second expression peak during secondary cell wall formation in conifers only (Fig. 4B). The functional significance of this conifer-specific expression pattern remains to be determined."

More broadly, we wish to emphasize that a central purpose of our resource is precisely to generate testable hypotheses from expression patterns. We have revised the manuscript to more consistently distinguish between empirical observations and functional interpretations where relevant.

Finally, we note that one key prediction from our comparative analysis — that xyloglucan acetylation is absent or greatly reduced in conifers, based on the lack of xyloglucan-targeting TBL orthologs — has now been experimentally validated using LCMS analysis of primary cell wall material from *P. tremula* and *P. abies* (Fig. 5C). This demonstrates that the resource can generate predictions that are confirmed by independent experimental data.

5. ATAC-seq performed in only two species but interpreted for all six
ATAC-seq is conducted only for Populus tremula and Picea abies (Page 14, Paragraph 3). Nevertheless, regulatory conclusions are generalized to all six species.

Answer:

The reviewer is correct that ATAC-seq data were generated for only two species — *P. tremula* and *P. abies* — representing one dicot and one conifer. We did not intend to generalize the regulatory network to the other four species. The regulatory networks presented in Fig. 6C and 6D are explicitly labelled as the "*P. tremula* network" and "*P. abies* network," and the text discusses regulatory differences specifically between these two species.

The connection to the other four species is limited to expression data: we show that VND master regulators have conserved expression across all six species (Fig. 6A), while NST expression has diverged between dicots and conifers (Fig. 6B). These are empirical observations from the transcriptome data and do not depend on the ATAC-seq analysis. The regulatory network inference — which integrates chromatin accessibility, motif enrichment, and expression correlation — is presented only for the two species where ATAC-seq data are available.

We have gone through the text to make this distinction even clearer, ensuring that statements about regulatory architecture are explicitly attributed to *P. tremula* and *P. abies*.

Minor Comments
1. Language overly absolute or subjective

Statements such as “poorly understood” and “first high-resolution evo-devo resource” appear in the introduction, overstating novelty.

Answer: We have revised the language throughout the manuscript to be more measured.

2. Figures and textual descriptions mismatched; key results insufficiently visualized
1）Fig. 2C lacks clear developmental-region labels.
2）Fig. 3B’s “unique cliques” appear highly curated, but selection criteria are not described.

Answer:

(1) We have added lines marking wood formation stages to Fig. 2C for clarity. (2) The selection criteria for unique cliques are described in the Methods section ("Identifying cliques"): unique cliques are non-overlapping cliques with no genes in common, ensuring each gene appears in only one clique. We have clarified the figure legend to read: "Heatmap of unique cliques with conserved co-expression across all six species (i.e. non-overlapping cliques, selected by iteratively retaining only cliques with no genes already assigned to a previously selected clique). Each row is a clique of six genes, one from each species."

3）TBL gene expression (e.g., TBL29, TBL37; Page 12, Paragraphs 2–4) does not fully align with claims regarding conserved acetylation functions.

Answer:
The expression pattern of TBL29 (green traces in Fig. 5B) matches the expected pattern for xylan acetylation enzymes, with a clear peak during secondary cell wall biosynthesis in all three dicot species, fully consistent with the xylan biosynthetic gene *IRX9* (orange traces in Fig. 4C). Regarding TBL37, the reported enzymatic activity did not differentiate among hemicelluloses (Sun et al., 2020), and we do not make specific claims about its substrate. We have clarified the wording from "non-specific hemicellulose acetylation" to "unspecified hemicellulose acetylation" to make this distinction explicit.

3. Key QC and preprocessing steps not fully documented
Criteria for removing outliers (mentioned broadly Page 19, 2nd paragraph) lack explicit thresholds.
ATAC-seq QC metrics, replicate numbers, and peak counts are missing (Methods around Page 14).

Answer: Outlier removal was based on visual inspection of PCA and hierarchical clustering plots, applied iteratively with renormalization after each removal. All

removed samples are listed in Table S1 for full transparency. This visual approach is standard for spatial transcriptomics datasets where expression is expected to follow a smooth developmental gradient, and outliers are readily identifiable as samples that break this continuity. We have added a sentence to the Methods to clarify this rationale.

For ATAC-seq, we agree that QC metrics were missing. We have added a new supplementary figure (Fig. S7) with QC statistics including fragment size distributions, TSS enrichment, and peak counts for both species.

4. The consistency between sample replicates is not demonstrated.
Across the different data types (RNA-seq, ATAC-seq, etc.), the number of biological replicates per species and per condition is not clearly summarized, and the reproducibility between replicates is not shown. The manuscript would benefit from an explicit description of replicate numbers in the main text or a summary table, and from quantitative assessments of replicate consistency (e.g. correlation matrices, PCA clustering by replicate, basic QC metrics), so that the robustness of the downstream network and comparative analyses can be properly evaluated.

Answer: The number of replicate trees per species (3–4) is described in the first paragraph of the Results section, and all individual samples and replicates are listed in Table S1. We have updated the Legend-tab in Table S1 to give a clear overview of the samples. Replicate consistency for the RNA-seq data is shown in Fig. S2, where hierarchical clustering heatmaps demonstrate that gene expression patterns are highly reproducible across replicate trees within each species, with samples from the same developmental position clustering together across replicates. As noted in our response to major point 2, because the developmental series are not directly comparable across species on a sample-by-sample basis, we have not computed cross-replicate correlation matrices — but the heatmaps provide clear visual evidence of reproducibility.

For ATAC-seq, we wish to clarify that these data were generated from a single xylem scraping per species (not from the cryosectioned series) and therefore represent a bulk wood sample rather than spatially resolved data. The ATAC-seq is used solely for identifying open chromatin regions to anchor the motif analysis, not for spatial comparisons.

We have revised relevant text to clarify, especially regarding ATAC-seq where we agree with the reviewer that the text could have been clearer.

5. Data and code availability insufficiently documented
PlantGenIE usage is not described.

Answer: All code generating the results, figures, and tables in this manuscript is publicly available in the GitHub repository linked under "Code availability." PlantGenIE is an established web resource for plant genomics with its own publications and documentation; we deposited our data there to make it accessible to the community.

Reviewer #3:

This manuscript presents an ambitious and valuable comparative evo-devo study of wood formation, combining spatially resolved transcriptomics across six tree species (three dicots and three conifers) with chromatin accessibility and transcription factor motif analyses. The dataset spans over 250 million years of evolutionary divergence and represents an important community resource. The work has clear potential impact for plant biology, but several methodological and interpretational aspects require clarification and improvement before publication.

Answer: We thank the reviewer for recognising the scope and potential impact of our study, and we appreciate the constructive suggestions for improvement, which are addressed point by point below.

Major Comments

1. Figure labeling and interpretability: Several figures (Fig. 1C, Fig. 4B-D, Fig. 5A-B, Fig. 6A-B) would benefit from adding labeling of x-axes to indicate tissue types to improving readability for a general audience, for example, by including color bars similar to Fig. 3B.

Answer: Developmental transitions between tissue types/stages were marked with dotted lines in all the plots, but these were hardly visible. We have now made the dotted lines more prominent throughout all figures, and added a consistent description of what they represent ("vertical dotted lines mark, from left to right, the middle of the cambium, the end of the expansion zone and the end of the SCW zone") to all relevant figure legends. We have also added color bars indicating tissue types to Fig. 2C for improved readability.

2. Phylogenetic signal of expression conservation: The statement on Page 5 “corresponding samples within dicots and conifers showing much higher correlation (~0.75) than samples compared between the two lineages (~0.55; Fig. S3B)” is not supported by the current figure Fig. S3B. The bimodal distribution is apparent, but the figure lacks annotation to distinguish “corresponding samples”. Please provide evidence or additional visualization (e.g., sample grouping, labeling, or correlation heatmap) to substantiate this claim.

Answer: The bimodal distributions in Fig. S3B show the correlation values for all sample pairs between two species, computed using co-expressologs: i.e. the correlations underlying the heatmaps in Fig. 2C. Corresponding developmental samples (i.e. samples from similar developmental stages) appear along the diagonal in Fig. 2C (high correlation) while samples from different stages are off-diagonal (low correlation), producing the two modes. The higher peak is consistently at ~0.75 for within-lineage comparisons and ~0.55 for between-lineage comparisons. We have expanded the Fig. S3B legend to explain how the bimodal distribution relates to corresponding versus non-corresponding samples and how this connects to Fig. 2C. We have also added color bars indicating tissue types to Fig. 2C, a better figure text to Fig. 2C and revised the text in general - as we agree that our description was not clear.

3. Clarification of Fig. 2C and related conclusions: It is unclear what Fig. 2C is plotting: it is not explained whether rows/columns represent genes or samples, nor how “transition” or “steady-state” phases were defined. And it is not clear how Fig. 2C leads to this conclusion “our analyses also revealed the same major stages of wood formation, and the corresponding reprogramming events, seen in cluster analysis of genes (Fig. S2) (i.e. yellow squares of steady state expression in Fig. 2C, of which the SCW stage was the most apparent, separated by transitions/reprogramming events).”

Answer: We agree that the Fig. 2C legend was insufficient, and the corresponding text unclear. Each heatmap shows sample-by-sample correlations between two species, computed using co-expressologs. Rows and columns represent samples ordered by developmental position, so that comparisons near the diagonals correspond to samples from corresponding developmental stages. The yellow squares along the diagonal reflect groups of consecutive samples with high mutual correlation — indicating stable expression phases — while transitions between these squares indicate rapid transcriptome reprogramming. We have expanded the figure legend to explain this and added color bars marking the developmental stages.

4. Integration of ATAC-seq and transcriptome data: I am not an expert of wood biology, so it will help if the authors can specify which tissue(s) the ATAC-seq data were derived from relative to the spatially resolved transcriptomes. Because biological meaning of the network in Fig. 6C depends critically on this correspondence, the rationale for integrating motif enrichment in accessible chromatin from these ATAC-seq samples with transcriptome-based co-expression modules needs to be explicitly justified.

Answer: The ATAC-seq data were generated from xylem scrapings collected after bark removal, representing mostly developing xylem depositing secondary cell walls. This tissue therefore corresponds broadly to the SCW-forming and maturing xylem stages in our spatially resolved transcriptomes.

We acknowledge that the ATAC-seq and RNA-seq data differ in spatial resolution. Importantly, because the ATAC-seq represents a bulk xylem sample, it identifies regions that are accessible at some point during wood formation, but cannot resolve when during development a given region is open. The spatially resolved transcriptome data provide this temporal dimension: by requiring that a TF both has its motif enriched in open chromatin near a module's genes and is co-expressed with that module across the developmental series, we can predict not only which regulatory interactions occur but also when during wood formation they are active.

We have added further justification for integrating these two datasets of different resolution in the revised text.

5. Claim of purifying selection (Page 14, last paragraph): The statement that “strong purifying selection” is indicated by association of TFs (VNDs, MYB46/83) with SCW modules is not supported. Motif conservation and inferred regulatory association do not constitute evidence of purifying selection. Moreover, motif presence in accessible regions does not necessarily indicate TF binding, and binding sites may have shifted (i.e., a TF can bind to promoters of orthologous genes in two species but the actual binding site might have moved). A more accurate statement would be that these genes “share conserved network targets”.

Answer: We agree — motif conservation and regulatory association do not constitute evidence of purifying selection. We have replaced "indicating strong purifying selection on their binding sites and a common origin of the regulatory network of wood formation" with "suggesting conserved gene targets and a common origin of the regulatory architecture underlying wood formation." We thank the reviewer for this important distinction.

6. AlphaFold analysis and interpretation: It is unclear whether AlphaFold predictions were run multiple times with different random seeds because the predicted structures could be different from different random seeds. The y-axis label (“model quality”) in Fig. S10 should be defined, and the connection between predicted interaction quality and the conclusion that “master regulators are predicted to frequently interact” needs clarification. Furthermore, were these predicted complexes linked to co-expression modules? Currently, this analysis does not convincingly support the conclusion about regulatory complexity.

Answer: As described in our response to Reviewer 2 (point 3.3), all AlphaFold3 predictions were run with three independent random seeds, each generating three diffusion samples, with highly consistent ranking scores across runs. We have expanded the Methods section (and the Fig. S10 legend) to define the ranking score (a composite of ipTM, pTM, disorder fraction, and clash penalties) and to describe the experimental design.

To clarify: the AlphaFold3 TF–TF interaction predictions are not linked to co-expression modules. They address a separate question — whether the transcription factors in the regulatory networks (Fig. 6C,D) are predicted to physically interact with each other. The finding that predicted TF–TF interactions are more frequent among aspen and Arabidopsis master regulators than among spruce regulators is consistent with, but independent of, the flatter network structure inferred for spruce from the motif and expression analyses, but both lines of evidence point to a more complex regulatory architecture in dicots than in conifers. We see that this was not clear in the concluding paragraph of the GRN-section and we have therefore revised the text.

7. Regulatory network architecture (Page 15): The claim that the P. tremula network “adopts a layered architecture with feed-forward loops” should be directly supported with a figure or network motif analysis. Similarly, the statement that the P. abies network is “flatter” requires supporting evidence, e.g., is the lack of VND to MYB edges due to lack of accessible regions in the MYB promoters or the lack of VND motifs? Displaying the ATAC-seq profiles and motif presence for these two species side-by-side could help support these claims.

Answer: The layered architecture is directly visible in Fig. 6C, where VNDs/NSTs regulate MYBs (layer 1 to 2), and MYBs in turn regulate biosynthetic genes (layer 2 to 3), with feed-forward links from layer 1 directly to layer 3. These regulatory links are based on motif enrichment in open chromatin combined with expression correlation, with all scores in Table S6. In the *P. abies* network (Fig. 6D), VNDs and MYBs instead regulate biosynthetic genes more in parallel, with fewer inter-layer connections — which is what we mean by "flatter."

The reviewer raises an excellent point about what explains the absence of VND-to-MYB edges in spruce. To address this, we have added a new supplementary figure (Fig. S9) showing ATAC-seq peak profiles at the MYB46/83 loci in both species. These show that the MYB promoter regions are accessible in both species, but that VND-associated motifs are absent in spruce, indicating that the flatter network reflects motif-level differences rather than a lack of chromatin accessibility. We have revised the text accordingly.

8. Module detection and network inference methods: For transcriptional module, why was a custom clustering method used instead of many established approaches such as WGCNA? It is fine to use a custom method, but he manuscript should show that the genes in the resulting modules are indeed co-expressed, and the average profiles for the module accurately represent the expression patterns of genes in the module. This is essential because module-level expression patterns were used for

GRN inference and TF assignment, directly influencing downstream conclusions about the interspecies network differences.

Answer: We used a coverage-based algorithm rather than WGCNA because our goal was to identify compact, non-overlapping modules centred on the most highly connected genes in the network, ensuring that each module has a well-defined representative expression profile. This approach was previously validated in our AspWood study (Sundell et al. 2017), where it successfully identified biologically meaningful modules corresponding to distinct stages of wood formation. We have made this clear in the method section.

The reviewer is right that co-expression within modules and the representativeness of average profiles should be demonstrated. We have added a new supplementary figure (Fig. S8) showing the expression profiles of all genes within each module used in the regulatory network analysis, confirming that the modules shown in the GRN are coherent.

9. ATAC-seq data quality and reporting: Quality control metrics are missing for the ATAC-seq datasets. Please include information such as read counts, unique mapping rate, fragment length distribution, and TSS enrichment scores see Yan et al Genome Biol 2020 https://doi.org/10.1186/s13059-020-1929-3, Ou et al BMC Genomics 2018 https://doi.org/10.1186/s12864-018-4559-3). The number of called peaks and representative genome browser tracks for key loci would greatly strengthen confidence in the data integration and conclusions.

Answer: We agree that ATAC-seq QC metrics were missing from the original submission. As noted in our response to Reviewer 2, we have added a new supplementary figure with QC statistics including fragment size distributions, TSS enrichment scores, and peak counts for both species. We have also added genome browser tracks of the MYB46/83 loci (middle layer regulators in the GRN), which support the motif-based regulatory inferences presented in the main text (Fig. S9).

10. It seems the public website that the author cited (plantgenie.org) only provides visualization of the data. It is unclear from the manuscript whether the raw or processed transcriptome and ATAC-seq data have been deposited in a public repository.

Answer: Raw RNA-seq reads are deposited in the European Nucleotide Archive with accession numbers listed in the Methods section for all six species. We have now also added the ENA accession numbers for the ATAC-seq data, which were inadvertently omitted from the original submission. Normalized expression tables are provided in Table S2. PlantGenIE provides interactive visualization and query access to the processed data as a community resource.

REVIEWER COMMENTS

Reviewer #1 (Remarks to the Author):

The authors have convincingly addressed all my concerns. As I mentioned before, this work certainly represents a valuable and technically impressive contribution to the fields of tree biology and evo-devo research and I appreciate the author's achievements.

We thank the reviewer for the positive assessment and for constructive feedback that improved the manuscript.

Reviewer #2 (Remarks to the Author):

I appreciate the authors' revisions and the added LCMS validation, but I remain concerned that the main conclusions are still stronger than the evidence supports.

1. The manuscript makes broad claims about dicot–conifer regulatory divergence over ~250 Myr, but the actual design is much narrower: six species total, only three per lineage, one genotype per species, and only 3–4 clonal replicate trees per genotype. These replicates address within-genotype technical/biological reproducibility, but they do not test genotype-level, population-level, species-level, or environmental variation. Moreover, the species were sampled across different sites and years. Thus, the study effectively compares six sampled genotypes under non-equivalent conditions, yet interprets the results as lineage-level evolutionary conservation or rewiring.

We have now added a Limitations paragraph acknowledging design features and their implications, and have moderated language referring to evolutionary timescales throughout the manuscript.

That said, we respectfully maintain that our conclusions are appropriate given the study design, and would push back on the premise that the limitations described by the reviewer would affect the validity of our positive findings.

Our evolutionary inferences rest exclusively on positive findings: conserved or lineage-consistent co-expression patterns observed concordantly across three or six species. The reviewer's concern — that non-equivalent sampling conditions (different sites, years, and genotypes) could compromise conclusions — applies straightforwardly to false negatives: environmental or genotypic differences between species may cause us to miss conservation that is genuinely present. It does not, however, apply to false positives. For sampling noise to generate spurious

lineage-level concordance, it would need to be structured in exactly the same lineage-specific way across all three dicot species sampled independently at different sites and years, and across all three conifer species sampled independently under different conditions. This is biologically implausible. Our design is therefore conservative by construction: the conditions the reviewer describes as limitations make it harder, not easier, to find the concordant patterns we report.

Sampling one genotype per species without population-level replication is standard practice in all comparative transcriptomics studies drawing evolutionary conclusions, across mammals, birds, and plants alike. No published evo-devo transcriptomics study operates at the scale of population-level sampling, as this would require data from hundreds or thousands of individuals per species.

2. The regulatory-network conclusions are even more underpowered. ATAC-seq was performed in only 2 of the 6 species — P. tremula and P. abies, i.e. one species per lineage — and from bulk xylem scrapings rather than spatially matched samples. Therefore, the key GRN conclusion is based on a 1-vs-1 comparison, not a replicated dicot-versus-conifer design. A two-species, bulk-accessibility dataset cannot establish a general lineage-level difference in regulatory architecture.

We agree that the regulatory network analysis is based on two species and explicitly acknowledge this as a limitation of the ATAC-seq component of the study. A replicated dicot-versus-conifer ATAC-seq design at the spatial resolution of our transcriptomics data would require resources and sample sizes that are not currently feasible for mature tree wood tissue, and to our knowledge no such dataset exists for any tree system.

We would note, however, that the two-species limitation affects the generalisability of the GRN conclusions, not their internal validity. The candidate regulatory differences we report between *P. tremula* and *P. abies* are supported by four independent and methodologically distinct lines of evidence: (1) co-expression patterns from the full six-species transcriptome dataset; (2) open chromatin accessibility from ATAC-seq; (3) transcription factor motif enrichment analysis; and (4) AlphaFold3-predicted protein–DNA interactions. The convergence of these independent lines on the same regulatory differences — specifically the presence of VND/NST binding motifs at MYB46/83 loci in *P. tremula* but not *P. abies* — substantially strengthens confidence in the species-specific findings, even in the absence of broader phylogenetic replication. We have been careful throughout the manuscript to frame these as candidate networks for the two studied species, and have added an explicit acknowledgement of the two-species scope to the Limitations paragraph.

3. The analytical conclusions also depend on several user-defined thresholds: the top 3% of co-expression links, FDR < 0.05 motif enrichment, Pearson correlation >

0.7 for TF–module assignment, and custom module definitions. These choices are central to the reported cliques and GRN differences, yet the manuscript does not show that the main biological conclusions remain stable across alternative reasonable thresholds or standard methods.

We addressed this concern in detail in our previous revision. Briefly: the 3% co-expression threshold was justified with a saturation analysis (Fig. S11) showing diminishing conservation above this value. The regulatory network inference is based on stringent statistics with FDR-corrected p-values for both motif enrichment (FDR < 0.05) and expression correlation (FDR < 0.05 with an additional Pearson correlation > 0.7 effect size filter). The module detection approach was previously validated in the AspWood study. Importantly, known SCW regulators consistently appear at the top of the regulatory network rankings regardless of the specific thresholds chosen, demonstrating the robustness of the key biological conclusion that the aspen network mirrors that of Arabidopsis while the spruce network has a noticeably simpler architecture (e.g. no links between top-layer VND/NST and middle-layer MYB46/83).

4. Finally, the manuscript contains very limited functional validation. The added LCMS experiment validates one cell-wall prediction in two species, but the main claims about NST/VND/MYB rewiring, H3 variant function, POK2 expression, and GRN architecture remain based on co-expression, motif enrichment, bulk chromatin accessibility, and AlphaFold predictions. There is no direct perturbation, no TF binding validation, and no functional test of the inferred regulatory hierarchy.

We refer the reviewer to our previous response, where we addressed this point in detail. Functional perturbation experiments in mature trees are not practically feasible, and our computational predictions are supported by multiple independent lines of evidence. The LCMS validation demonstrates that the resource generates experimentally confirmed predictions, and we consider this scope appropriate for a comparative genomics study of this kind.

The work may be useful as a descriptive transcriptomic resource, but it is not convincing as evidence for lineage-level regulatory rewiring. The claims should be substantially narrowed.

We hope that the language modifications and added Limitations pragraph, combined with the clarifications provided above, adequately address the reviewer's concerns.

Reviewer #3 (Remarks to the Author):

I thank the authors for their careful revision. Most of my previous comments have been adequately addressed, and I have only two remaining minor comments.

We thank the reviewer for the careful and constructive engagement across both rounds of review. The reviewer's comments have genuinely improved the manuscript.

Round 1 review comment #6: AlphaFold analysis

The authors’ response to my previous comment is satisfactory. I only ask that they explicitly specify the source of the reported “ranking score” in the Methods or Fig. S10 legend. I assume this refers to the ranking score output directly from AlphaFold3, but this should be stated clearly.

We confirm that the ranking score is the score directly provided from AlphaFold3, and have stated this explicitly in the Methods and Fig. S10 legend.

Round 1 review comment #9: ATAC-seq quality metrics and interpretation

I appreciate that the authors have added ATAC-seq QC metrics, including fragment size distributions, TSS enrichment scores, peak counts, and genome browser tracks. These additions are helpful and improve the reporting of the dataset.

However, the newly provided QC metrics suggest some limitations in ATAC-seq data quality. Specifically, the fragment length distributions appear to lack a clear mono-nucleosome-bound fragment peak around ~200 bp, which is typically expected in ATAC-seq data (Fig 1B in Yan et al., Genome Biology 2020, https://doi.org/10.1186/s13059-020-1929-3; Troubleshooting 3 in Marand et al., 2021 https://doi.org/10.1016/j.xpro.2021.100737). In addition, the reported TSS enrichment scores appear relatively low. Although mammalian ENCODE standards (https://www.encodeproject.org/atac-seq/#standards) may not translate directly to plant tissues, TSS enrichment scores above ~5–6 are generally expected for high-quality ATAC-seq data.

To help readers assess the data quality more fully, I recommend that the authors provide full TSS enrichment profiles, rather than only summary scores. A plot similar to Yan et al. 2020, Fig. 1C, would be useful for showing whether the expected enrichment around transcription start sites is present.

We have now added full TSS enrichment profiles to Fig. S7, which confirms the expected enrichment of open chromatin immediately around transcription start sites in both species.

We thank the reviewer for their continued engagement with the ATAC-seq data quality question. This prompted us to investigate the expected ATAC-seq quality metrics for bulk tissue preparations from non-model organisms in more depth than we had previously. We summarize our findings below:

Fragment length distribution. The expectation of discrete mononucleosomal (~200 bp) and dinucleosomal (~400 bp) peaks in the fragment size distribution (Yan et al., 2020; Buenrostro et al., 2013) was established in experiments using purified, homogeneous cell populations — sorted human T cells, mammalian cell lines, and unicellular yeasts. We note that the troubleshooting guidance cited by the reviewer (Marand et al., 2021) refers specifically to single-cell ATAC-seq from protoplasted maize seedling cells: a very different experimental system in which homogeneous, isolated nuclei are the input material and nucleosome periodicity is therefore more readily detectable. These standards do not translate straightforwardly to bulk preparations from mature tree wood tissue.

Developing wood scraped from *Populus tremula* and *Picea abies* trees encompasses a highly heterogeneous mixture of cell types executing distinct developmental programmes simultaneously: cambium initials, xylem mother cells, differentiating fibres and tracheids at multiple stages of secondary cell wall deposition, ray parenchyma, and cells undergoing programmed cell death. When nucleosome arrays in co-occurring cell types are positioned at different genomic loci — as expected in cells with different transcriptional states — their signals cancel out in aggregate bulk data, attenuating or eliminating the periodic banding pattern. This is a well-documented consequence of tissue heterogeneity in bulk ATAC-seq, and precisely the limitation that motivated the development of single-nucleus approaches (Preissl et al., 2018; Fang et al., 2021; Dorrity et al., 2020). It has been noted explicitly in the plant ATAC-seq literature that bulk assays show "little variation in accessible regions across tissues, conditions, and even samples with different genetic backgrounds," with the leading explanation being that "true cell-type-specific accessibility [is] masked by cellular heterogeneity in bulk assays" (Lu et al., 2024; Dorrity et al., 2020).

The tissue-dependence of nucleosome periodicity in ATAC-seq data has been demonstrated directly in published studies of non-model tissues. Peng et al. (2021), working on equine tissues, reported that ATAC-seq from lamina — a relatively acellular connective tissue — produced a fragment size distribution in which only the nucleosome-free peak was observed, with no accompanying mono- or dinucleosomal banding, in explicit contrast to the clear laddering seen in liver from the same animal. The authors note that this does not constitute evidence of data failure, but reflects the cellular composition of the tissue. Halstead et al. (2020), optimising ATAC-seq from cryopreserved livestock tissues, showed that chromatin fragmentation occurring in complex tissues — including those with cells undergoing programmed cell death — produces uniform or shifted fragment size distributions that do not preclude meaningful identification of open chromatin regions. Both of these contexts are directly analogous to the developing wood tissue used in the present study.

To our knowledge, no published quality benchmark for ATAC-seq fragment size distributions exists for bulk tissue preparations from secondary xylem of gymnosperm or angiosperm trees, and the application of standards calibrated on sorted mammalian cell populations or unicellular organisms to this material is not warranted.

ENCODE TSS enrichment thresholds were established for mammalian cell lines sequenced at high depth, using well-curated annotation resources with precise TSS coordinates. Both the sequencing depth conventions and the annotation quality differ substantially from what is available for conifer and angiosperm tree genomes, where TSS annotations are less complete and gene models less refined. TSS enrichment scores are sensitive to annotation quality and lower values are routinely reported in published plant bulk ATAC-seq studies. The full TSS enrichment profiles now provided in Fig. S7 show the functionally appropriate pattern — clear enrichment at the TSS with flanking depletion — which is the relevant quality criterion for a non-standard system.

We are therefore confident that our ATAC-seq data are of suitable quality for the peak-based and motif-enrichment analyses presented, supported by: (i) the expected TSS enrichment profile shape (Fig. S7); and (ii) concordance of identified open chromatin regions with known regulatory loci (Fig. S9).

Buenrostro, J.D., Giresi, P.G., Zaba, L.C., Chang, H.Y. & Greenleaf, W.J. (2013) Transposition of native chromatin for fast and sensitive epigenomic profiling of open chromatin, DNA-binding proteins and nucleosome position. *Nat. Methods* **10**, 1213–1218. https://doi.org/10.1038/nmeth.2688

Dorrity, M.W., Alexandre, C.M., Hamm, M.O., Fields, S., Queitsch, C. & Cuperus, J.T. (2020) The regulatory landscape of Arabidopsis thaliana roots at single-cell resolution. *Nat. Commun.* **11**, 3316. https://doi.org/10.1038/s41467-020-17738-7

Fang, R., Preissl, S., Li, Y., Hou, X., Lucero, J., Wang, X., … Ren, B. (2021) Comprehensive analysis of single cell ATAC-seq data with SnapATAC. *Nat. Commun.* **12**, 1337. https://doi.org/10.1038/s41467-021-22266-1

Halstead, M.M., Kern, C., Saelao, P., Wang, Y., Chanthavixay, G., Medrano, J.F., … Ross, P.J. (2020) Systematic alteration of ATAC-seq for profiling open chromatin in cryopreserved nuclei preparations from livestock tissues. *Sci. Rep.* **10**, 5230. https://doi.org/10.1038/s41598-020-61678-9

Lu, C., Wei, Y. & Abbas, M. (2024) Application of single-cell assay for transposase-accessible chromatin with high throughput sequencing in plant science: advances, technical challenges, and prospects. *Int. J. Mol. Sci.* **25**, 1479. https://doi.org/10.3390/ijms25031479

Marand, A.P., Zhang, X. & Nelson, J.G. (2021) Profiling single-cell chromatin accessibility in plants. *STAR Protoc.* **2**, 100737. https://doi.org/10.1016/j.xpro.2021.100737

Peng, S., Bellone, R.R. & Petersen, J.L. (2021) Successful ATAC-seq from snap-frozen equine tissues. *Front. Genet.* **12**, 641788. https://doi.org/10.3389/fgene.2021.641788

Preissl, S., Fang, R., Huang, H., Zhao, Y., Raviram, R., Gorkin, D.U., … Ren, B. (2018) Single-nucleus analysis of accessible chromatin in developing mouse forebrain reveals cell-type-specific transcriptional regulation. *Nat. Neurosci.* **21**, 432–439. https://doi.org/10.1038/s41593-018-0079-3

Yan, F., Powell, D., Curtis, D.J. & Wong, N.C. (2020) From reads to insight: a hitchhiker's guide to ATAC-seq data analysis. *Genome Biol.* **21**, 22. https://doi.org/10.1186/s13059-020-1929-3

I do not view this issue as a barrier to publication, given the overall strengths of the manuscript and the importance of the spatial transcriptomic data. However, because ATAC-seq in these plant samples is understandably challenging, I recommend that the authors modestly temper the language around the ATAC-seq-based GRN conclusions and present these results as supportive rather than definitive evidence. For example, I suggest replacing definitive language such as “The resulting robust TF-module networks”, “the regulatory network,” “top layer master regulators,” “the structure of the P. tremula regulatory network”, “our analysis paints a picture of a more streamlined network” with more cautious phasing such as “candidate TF-module networks, “candidate regulatory network”, “proposed top layer master regulators, “the structure of the candidate P. tremula regulatory network” , and “our analysis is consistent with a more streamlined network”, etc.

We have implemented all the suggested language changes in the manuscript. We note that the term "robust" in "robust TF-module networks" was intended to reflect a methodological property — namely that our network inference relies on enrichment of TF motifs in co-expression modules rather than individual motif matches.
