# Supplementary material for: Comparative regulomics of wood formation across dicot and conifer trees
Source: Nat Commun. 2026 Jul 22;17:8916. doi: 10.1038/s41467-026-75624-2 (PMC13503837; doi:10.1038/s41467-026-75624-2)
Supplement: Supplementary file 2 [file 41467_2026_75624_MOESM1_ESM.pdf]

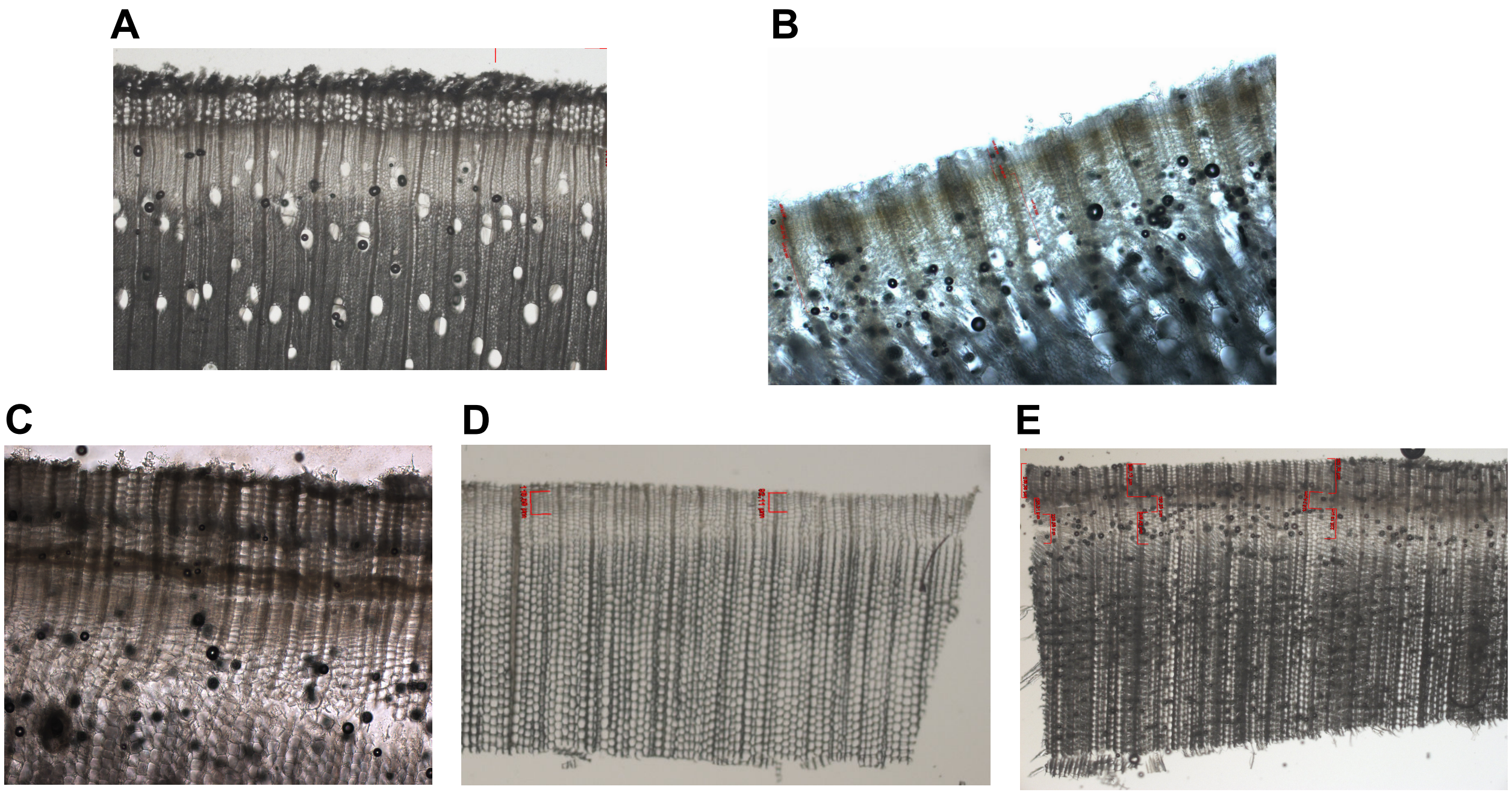

Supplementary Figure 1. Wood transverse cross sections from hardwood and softwood tree samples. The cortex was first removed before using a cold scalpel (-20C) in a cryo-microtome to cut a transverse section. Sections were then visualised under a light microscope. (A) *Betula pendula* (birch). (B) *Prunus avium* (cherry). (C) *Picea abies* (Norway spruce). (D) *Pinus sylvestris* (Scots pine). (E) *Pinus contorta* (lodgepole pine). Cryosection images for *Populus tremula* (aspen) were previously published in Sundell *et al*. (2017)[13].

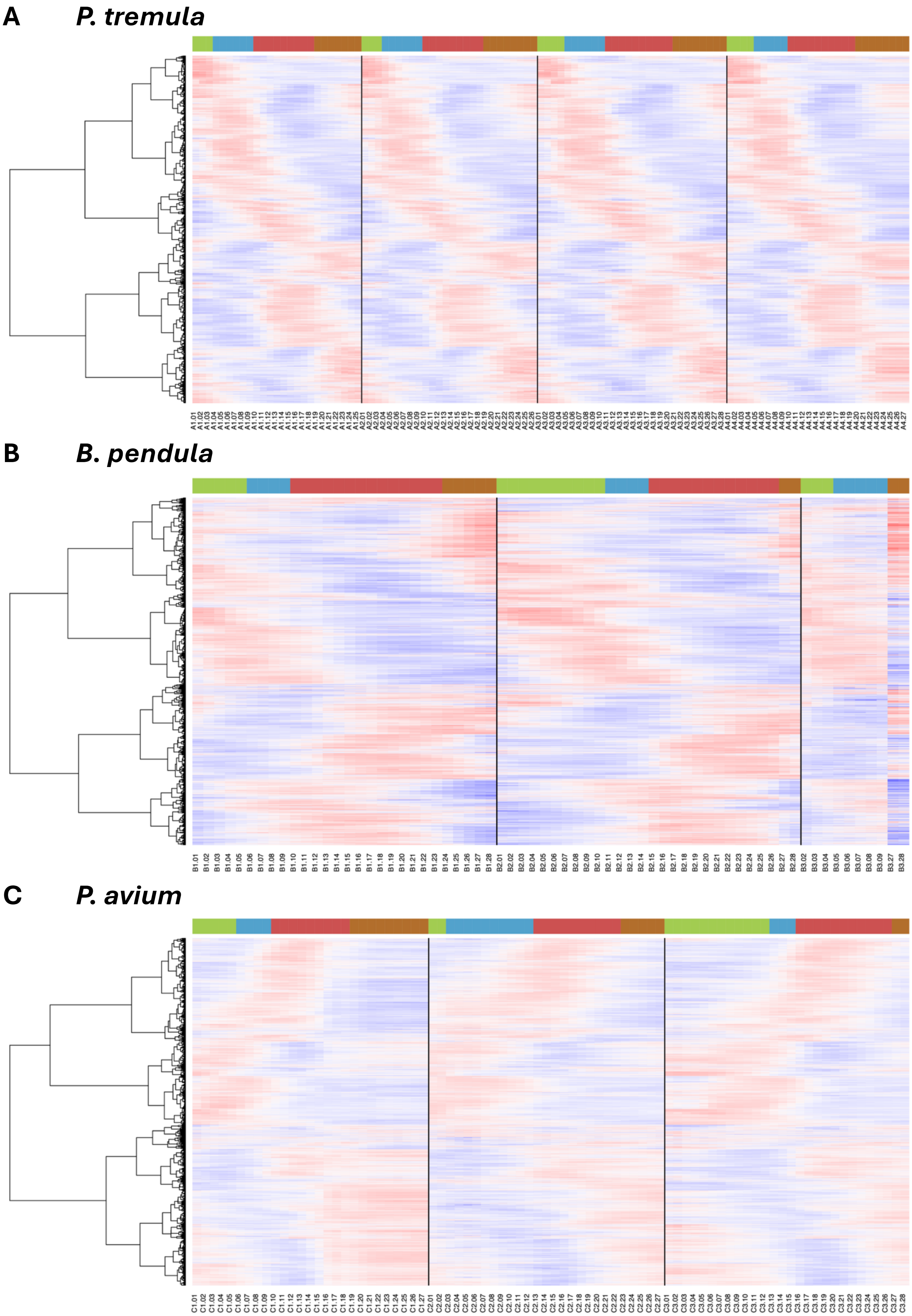
A
P. tremula
B
B. pendula
C
P. avium

D ***P. abies***

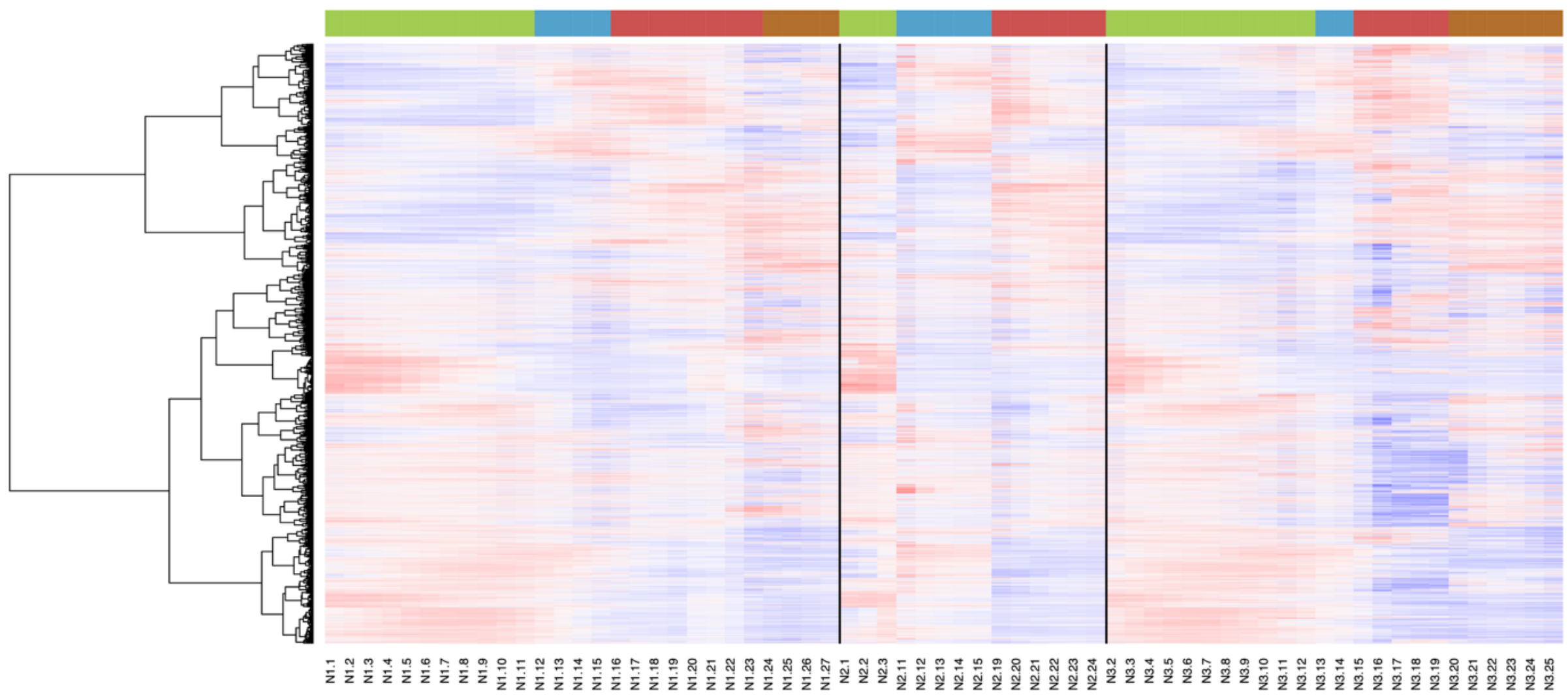

E ***P. sylvestris***

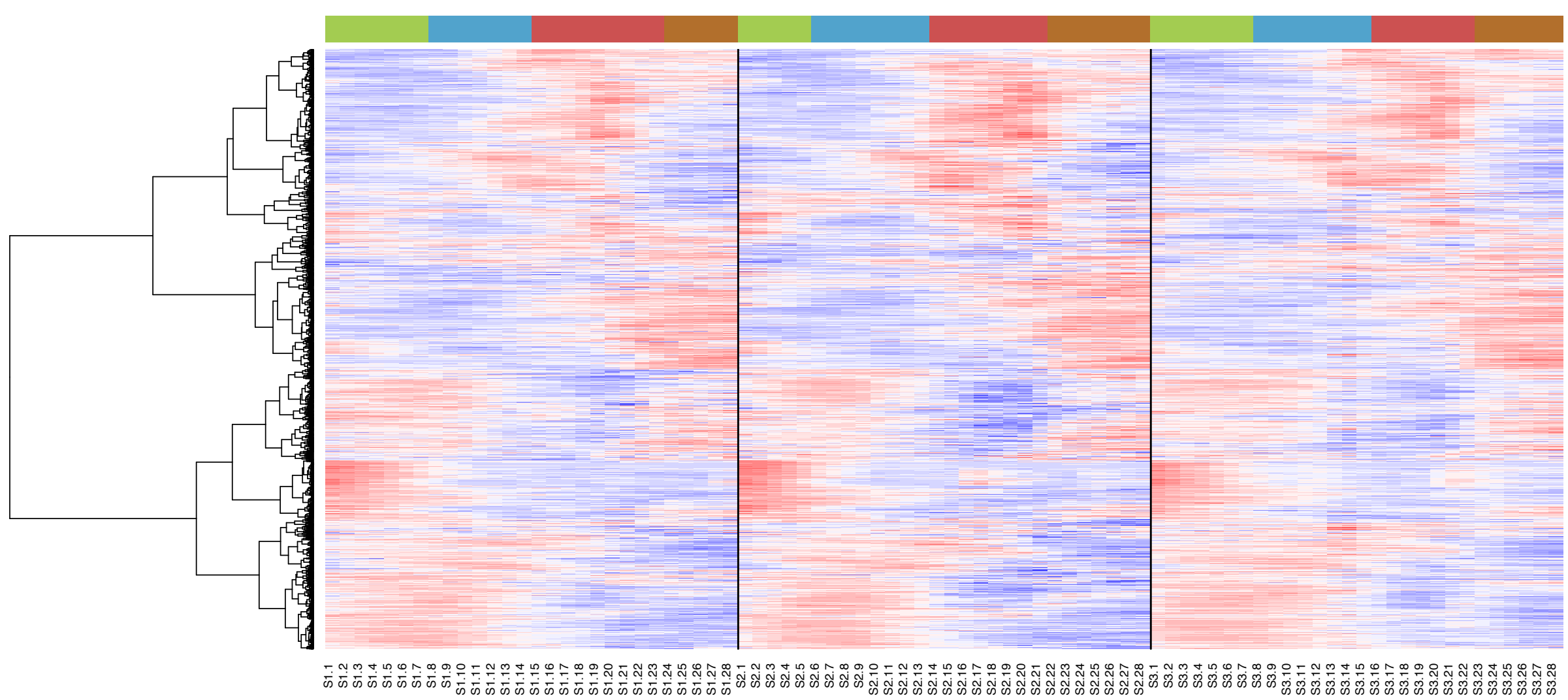

F ***P. contorta***

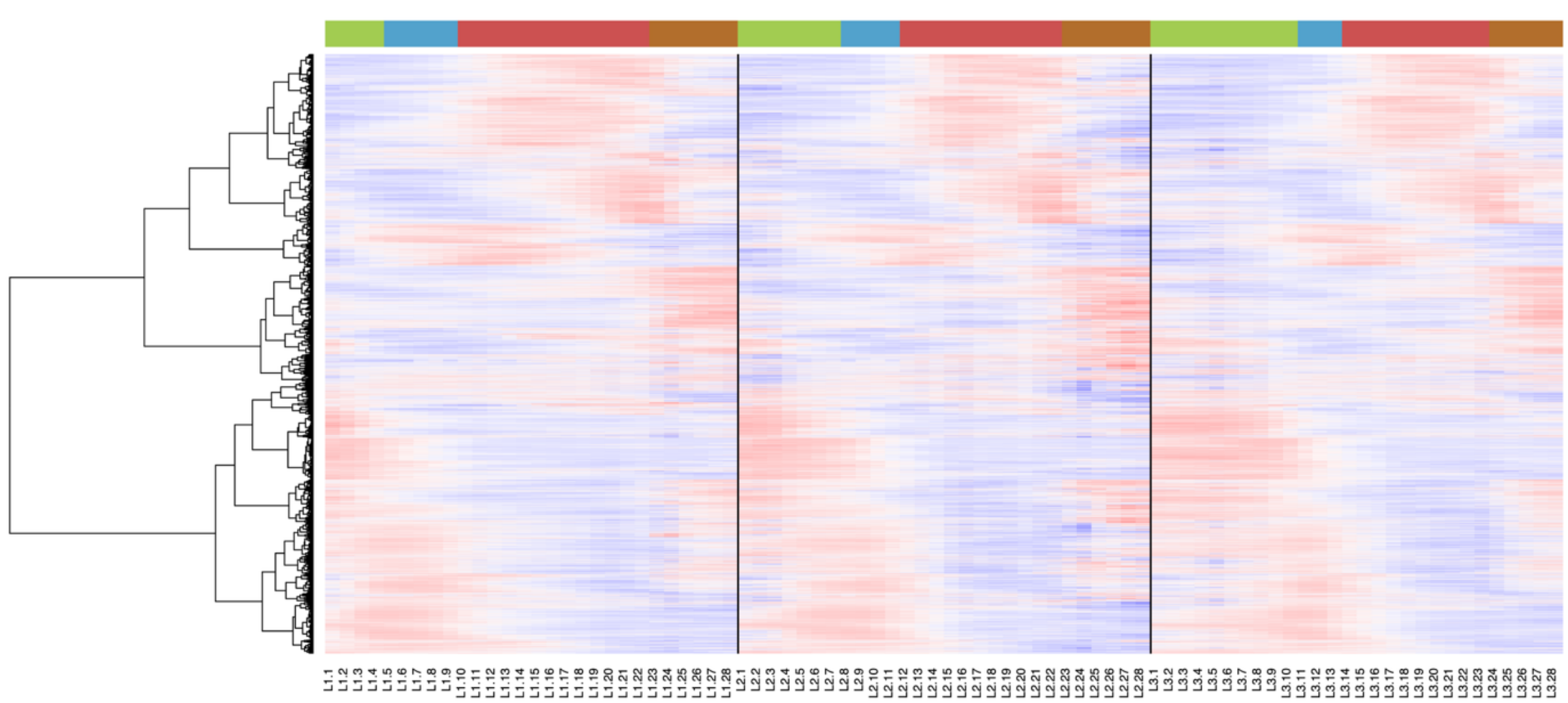

Supplementary Figure 2. Heatmaps for each species. For each species (A-F), a heatmap of all expressed genes (rows) and all retained samples (column) are shown. The expression values of each gene are scaled and colored from blue (lowest expression) to red (highest expression). Each replicate tree is separated by a black line. The horizontal color bar marks four sample clusters largely coinciding with the developing phloem (green), the cell expansion zone (blue), the secondary cell wall zone (red) and the maturing xylem zone (brown).

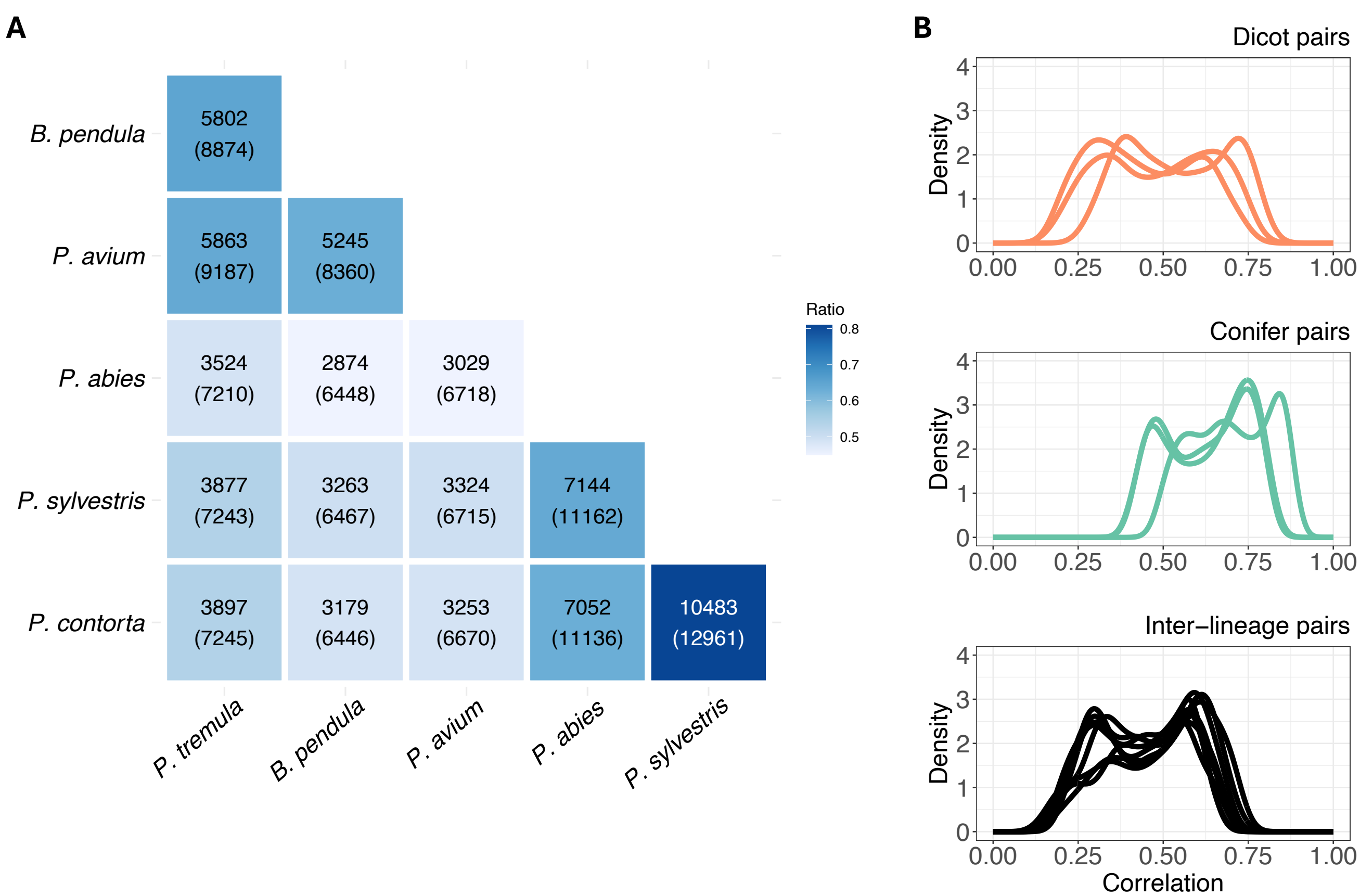

Supplementary Figure 3. Comparisons of six wood transcriptomes. (A) The number of orthogroups with at least one co-expressologs - for each of the 15 species pairs. The total number of orthogroups are in parentheses. Colors indicate the fraction of orthogroups with co-expressologs (Ratio: from dark blue to light blue). (B) The distribution of correlations between samples from different species pairs: three dicot pairs, three conifer pairs and nine inter-lineage pairs (dicot-conifer pairs). The rightmost peaks correspond to correlations near the diagonals in Figure 2C (samples from corresponding developmental stages), while the leftmost peaks correspond to off-diagonal comparisons (samples from different stages).

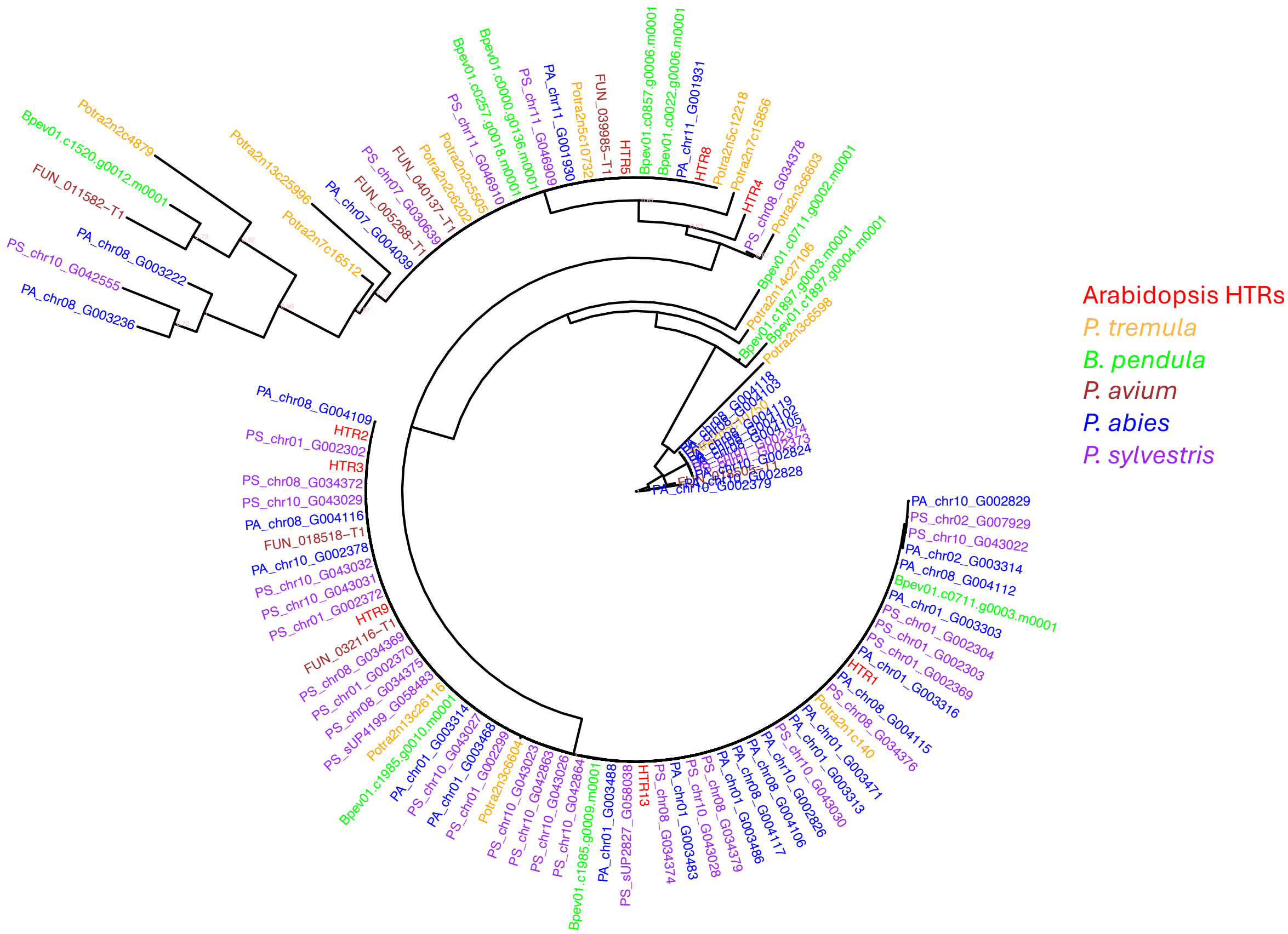

Supplementary Figure 4. H3.1 and H3.3 phylogeny. The phylogeny of the orthogroup (OG0000095) contained all Arabidopsis H3.1 (HTR1, 2, 3, 9 and 13) and H3.3 (HTR4, 5 and 8) genes.

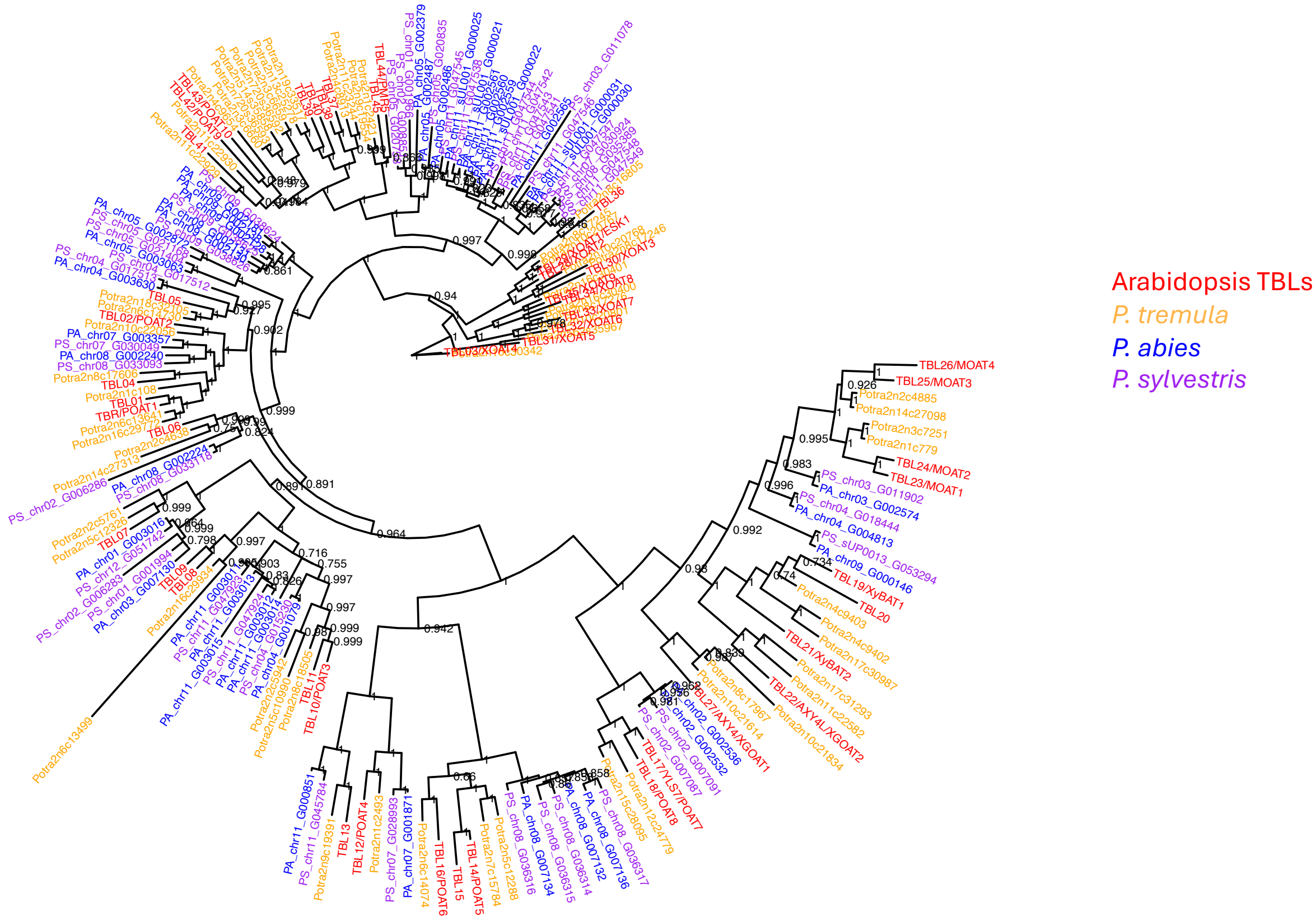

Supplementary Figure 5. Trichome Birefringence-Like (TBL) phylogeny. The common phylogeny of all genes in orthogroups containing Arabidopsis TBL genes.

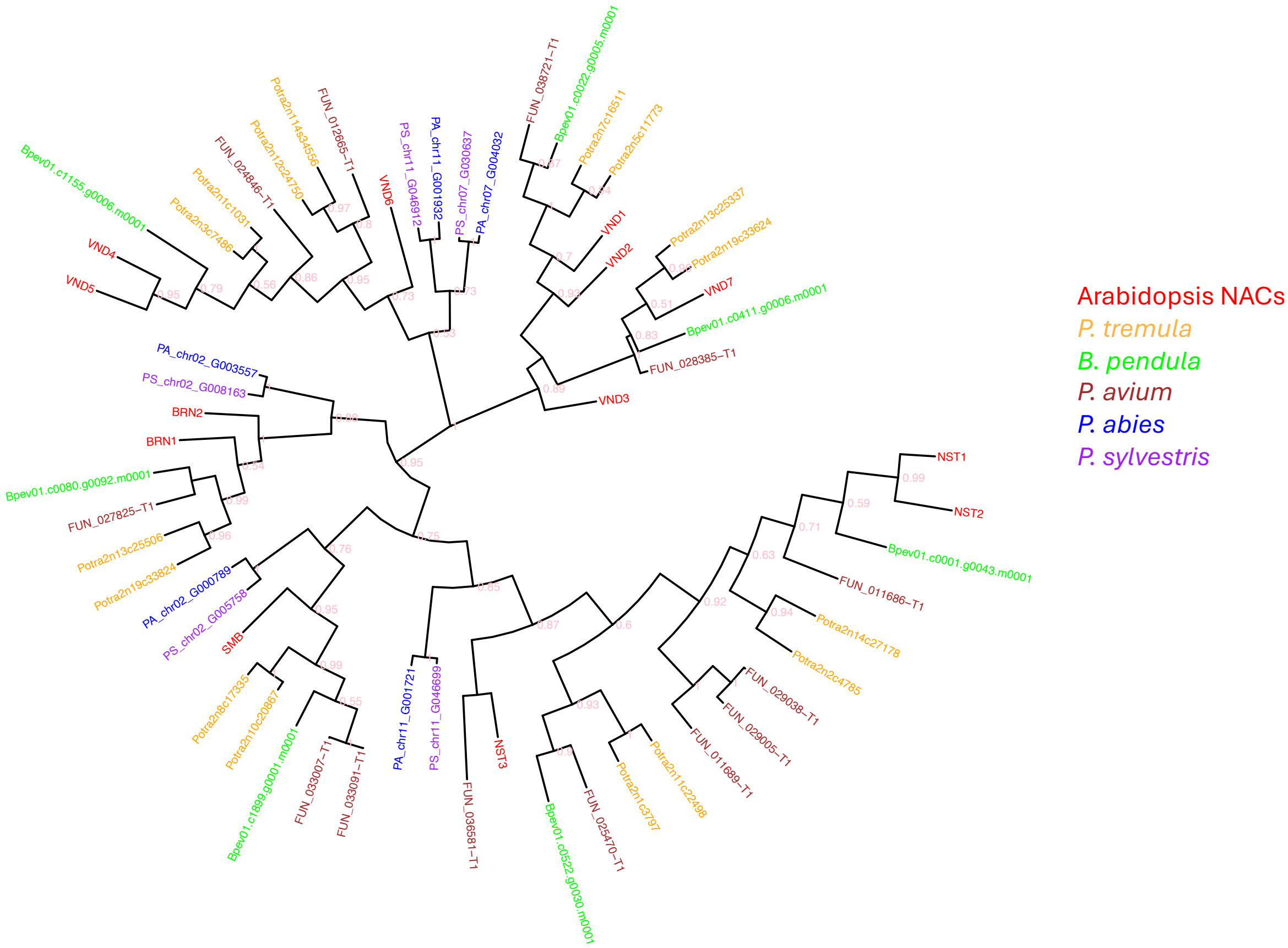

Supplementary Figure 6. NAC phylogeny. The phylogeny of NAC master regulators.

A Quality control

**Species**	**Read pairs**	**Unique reads**	**TSS enrichment score**	**Peak counts**
P. tremula	15,197,672	77%	3.065743	41830
P. abies	1,146,879,344	38%	3.970731	85608

B *P. tremula:* Fragment length

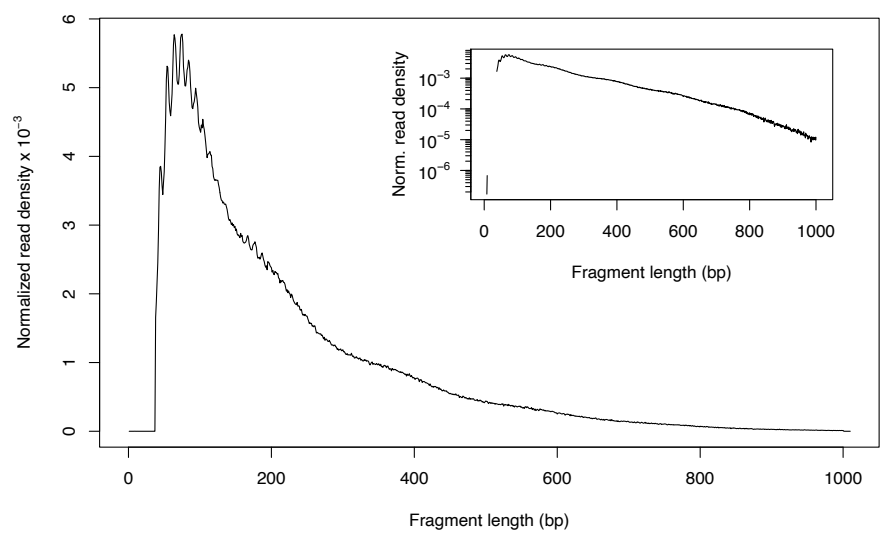

C *P. abies*: Fragment length

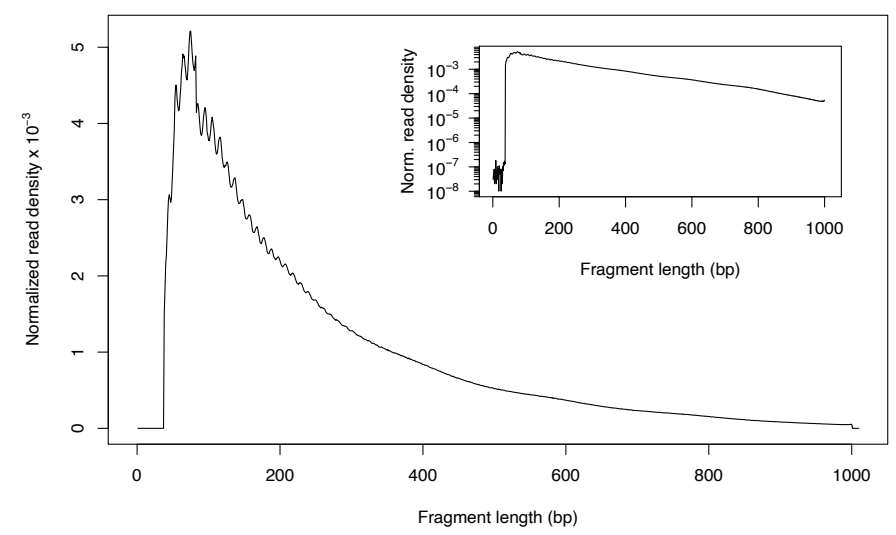

D *P. tremula*: TSS enrichment profile

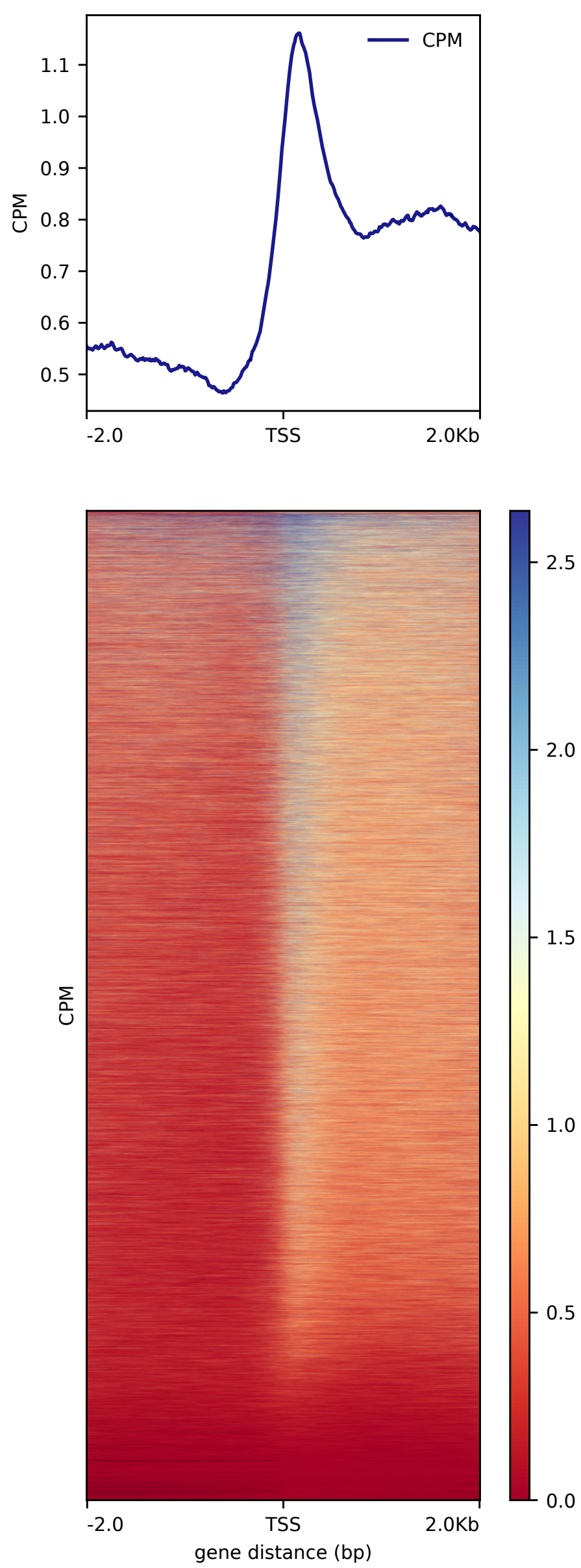

E *P. abies*: TSS enrichment profile

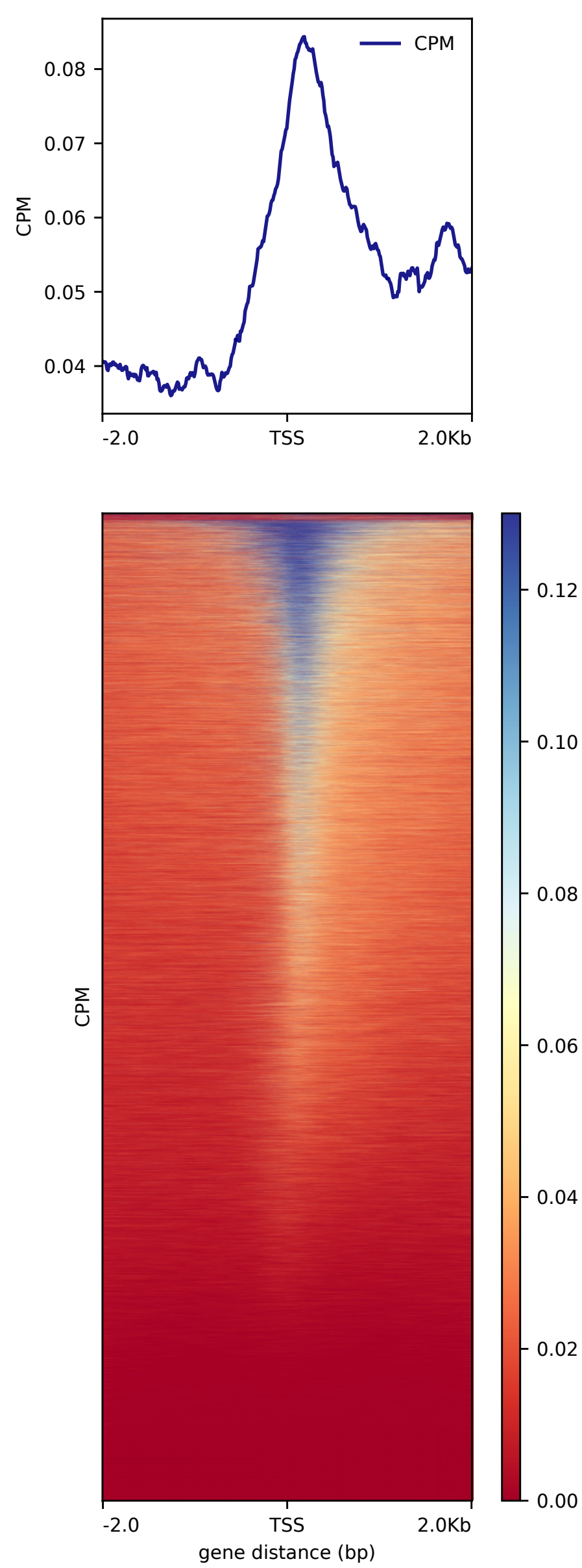

Supplementary Figure 7. ATAC-seq quality control. (A) Summary statistics for the ATAC-seq data of *Populus tremula* and *Picea abies*, including number of read pairs, proportion of uniquely mapped reads, transcription start site (TSS) enrichment score (a measure of signal-to-noise reflecting the expected enrichment of accessible chromatin near promoters), and number of called open chromatin peaks. (B, C) Fragment length distributions for *P. tremula* and *P. abies*. (D, E) TSS enrichment profiles for *P. tremula* and *P. abies*.

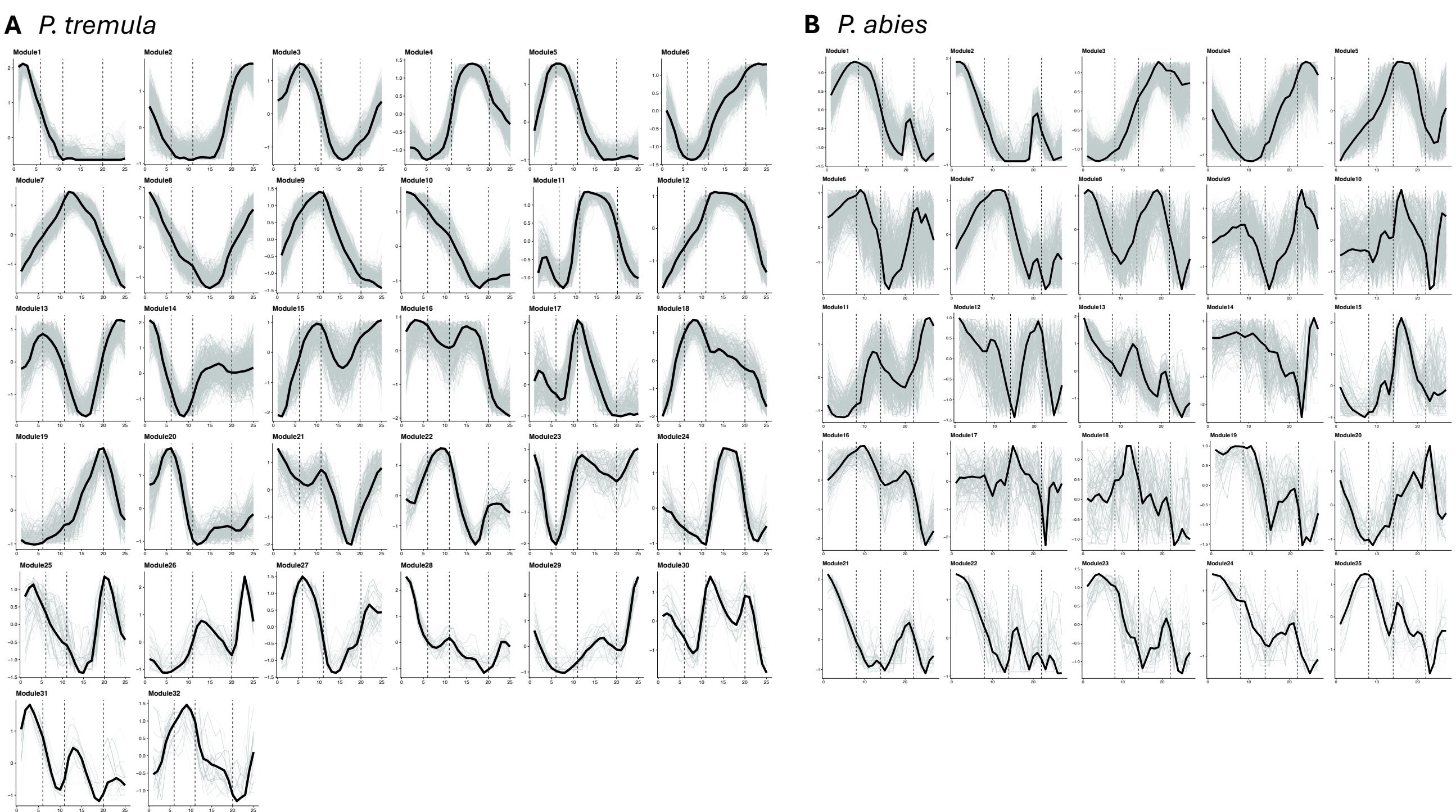

Supplementary Figure 8. Gene expression profiles of the gene regulatory network modules. For each module, the expression profile of the central gene (centroid) is shown as a thick black line, with other module members in thin grey lines. Expression values are scaled by subtracting the mean and dividing by the standard deviation. (A) *Populus tremula*. Marker genes from Figure 1C are located in the following modules: SUS6 in Module 1, CDC2 in Module 5, BFN1 in Module 6, EXPA1 in Module 9, and CesA8 in Module 11. (B) *Picea abies*. Marker genes from Figure 1C: CDC2 in Module 1, SUS6 in Module 2, CesA8 and BFN1 in Module 3, and EXPA1 in Module 7.

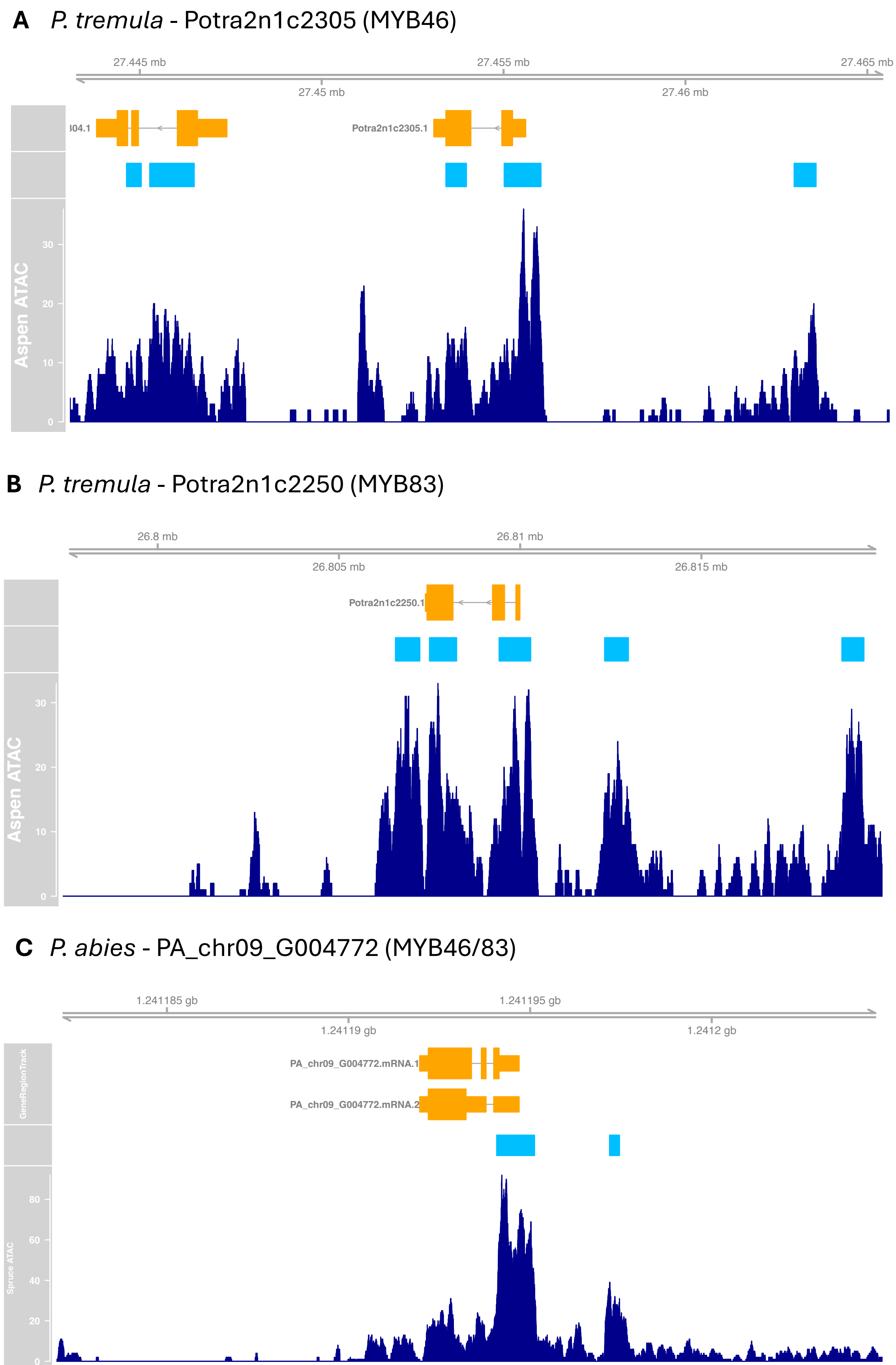
A P. tremula - Potra2n1c2305 (MYB46)
27.445 mb
27.455 mb
27.465 mb
27.45 mb
27.46 mb
Potra2n1c2305.1
Aspen ATAC
30
20
10
0
B P. tremula - Potra2n1c2250 (MYB83)
26.8 mb
26.81 mb
26.805 mb
26.815 mb
Potra2n1c2250.1
Aspen ATAC
30
20
10
0
C P. abies - PA_chr09_G004772 (MYB46/83)
1.241185 gb
1.241195 gb
1.24119 gb
1.2412 gb
GeneRegionTrack
PA_chr09_G004772.mRNA.1
PA_chr09_G004772.mRNA.2
Spruce ATAC
80
60
40
20
0

Supplementary Figure 9. ATAC-seq profiles at MYB46/83 loci. All three genes are on the negative strand. (A, B) ATAC-seq signal at the *Populus tremula* MYB46 and MYB83 loci, where open chromatin peaks upstream of the genes contain VND/NST binding motifs, consistent with the hierarchical regulation inferred in the *P. tremula* network (Figure 6C). (C) ATAC-seq signal at the *Picea abies* MYB46/83 ortholog. Open chromatin peaks are present upstream of the gene, but VND/NST binding motifs are absent, indicating that the lack of VND-to-MYB regulatory links in the *P. abies* network (Figure 6D) reflects missing binding sites rather than inaccessible chromatin. Called peaks are shown in blue.

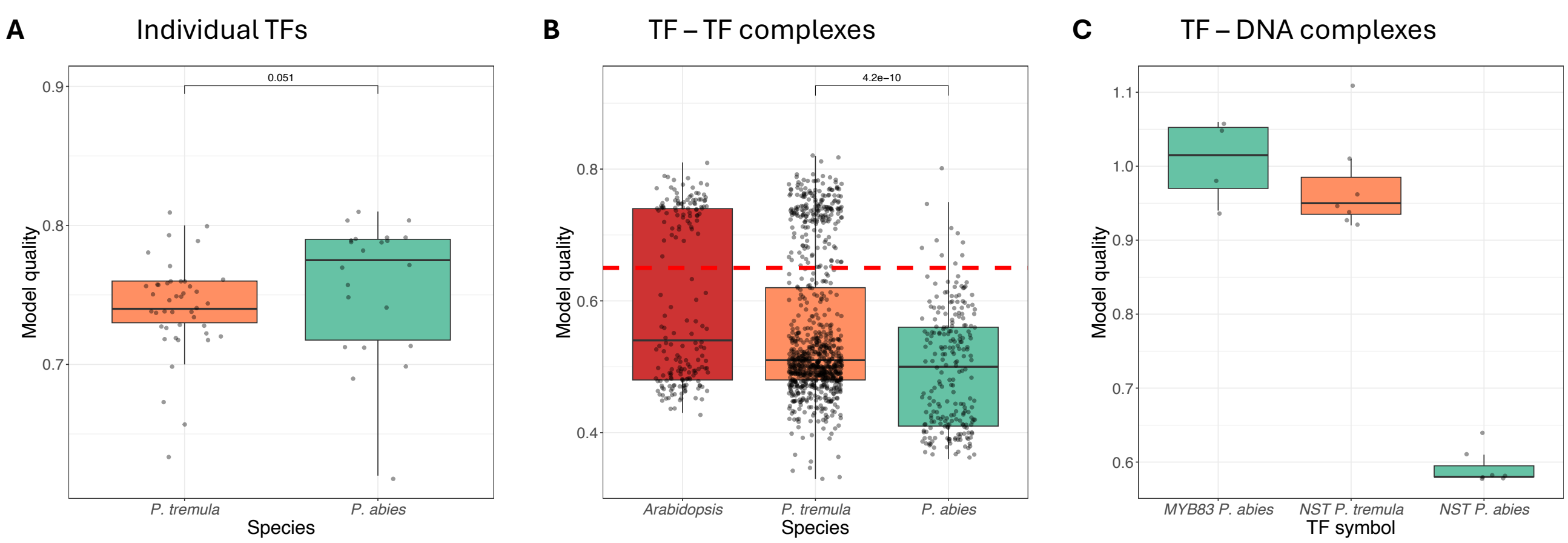

Supplementary Figure 10. AlphaFold predictions. (A) AlphaFold3 was used to predict the structure of all individual *Populus tremula* and *Picea abies* transcription factors (TFs) in the regulatory network from Figure 6CD. This confirmed that AlphaFold was able to build structure models of comparative quality in the two species (assessed by *Ranking score* reported directly by AlphaFold3, combining predicted interface accuracy, overall structural accuracy, disorder fraction, and clash penalties). (B) AlphaFold3 was then used to predict all TF – TF complexes by pairing all TFs in the regulatory networks. Arabidopsis was included as a control. Complexes with ranking scores above 0.65 are generally considered reliable in the literature, and this value also emerged as a natural threshold in our data. Using this threshold to predict putative TF-TF interactions, there were considerably more predicted interactions in Arabidopsis/*Populus tremula* than in *Picea abies*. (C) Finally, the regulatory network predicted NST as one of the top regulators of Secondary Cell Wall (SCW) Cellulose synthase A genes (CesAs) in *Populus tremula*, but not in *Picea abies*. AlphaFold3 was used to predict TF-DNA complexes between NST and its predicted DNA binding sites near SCW CesAs. The results showed that these sites are likely binding sites for NST in *Populus tremula* but not in *Picea abies*. As a control, we also predicted complexes between MYB83, predicted to be the top regulator of SCW CesAs in *Picea abies*, and its binding sites - showing that this is a likely TF-DNA interaction.

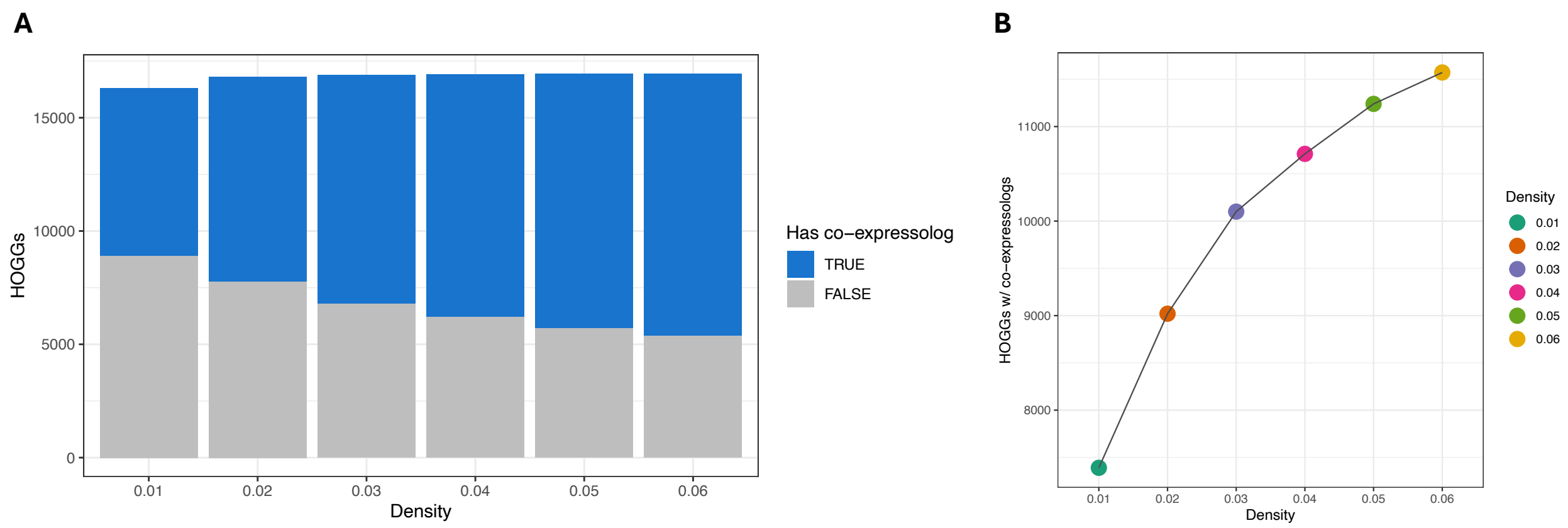

Supplementary Figure 11. ComPlEx threshold. (A) Number of HOGs (Phylogenetic Hierarchical Orthogroups) with and without co-expressologs as a function of the network density threshold. The number of orthogroups was accumulated across all 15 pairwise species comparisons. (B) Number of HOGs with co-expressologs as a function of the network density threshold.
