# Supplementary material for: Comparative regulomics of wood formation across dicot and conifer trees
Source: Nat Commun. 2026 Jul 22;17:8916. doi: 10.1038/s41467-026-75624-2 (PMC13503837; doi:10.1038/s41467-026-75624-2)
Supplement: Supplementary file 3 [file 41467_2026_75624_MOESM2_ESM.pdf]

Description of Additional Supplementary Files

File Name: Supplementary Data 1

Description: Cryosections and pooled samples for each species.

For each species and each replicate tree, a diagram is showing the original cryosection (Section), the sections pooled into samples for RNA-seq (Sample) and the anatomical observations made during cryosectioning (Tissue). Samples excluded from downstream analysis are marked in a separate column when applicable (Outlier)..

File Name: Supplementary Data 2

Description: Gene expression data sets.

For each species, a table is provided with genes as rows, samples as columns and VST-normalized expression as values.

File Name: Supplementary Data 3

Description: Co-expressologs.

For each species pair, ortholog pairs with significantly overlapping co-expression neighborhoods (i.e. co-expressologs) are listed (reciprocal one-sided hypergeometric test with False Discovery Rate-corrected p-values, see Methods for details).

File Name: Supplementary Data 4

Description: Cliques.

Tables of different types of cliques conserved across all species (Conserved), conserved within dicots and within conifers, but not across the two lineages (Differentiated) and conserved specifically in dicots or conifers (Specific). Each table exists in two versions: all cliques and unique cliques. Cliques computed for high resolution Phylogenetic Hierarchical Orthogroups (HOGs) are provided here, while cliques for standard orthogroups (OGs) can be found in the repository.

File Name: Supplementary Data 5

Description: Gene function enrichment.

Enriched Gene Ontology (GO) Biological Processes among genes in the different clique types in Supplementary Data 4 (one-sided Fisher exact test).

File Name: Supplementary Data 6

Description: Gene regulatory network.

Tables containing the inferred regulatory network for *P. tremula* and *P. abies*, as well as the Position Frequency Matrices (PFMs) for the analyzed transcription factor motifs (see Methods for details). Also included are the protein-protein and protein-DNA interactions predicted by AlphaFold.
