# Supplementary material for: Comparative regulomics of wood formation across dicot and conifer trees
Source: Nat Commun. 2026 Jul 22;17:8916. doi: 10.1038/s41467-026-75624-2 (PMC13503837; doi:10.1038/s41467-026-75624-2)
Supplement: Supplementary file 4 [file 41467_2026_75624_MOESM9_ESM.pdf]

nature portfolio

Corresponding author(s): Torgeir R Hvidsten

Last updated by author(s): 2026.04.20

Reporting Summary

Nature Portfolio wishes to improve the reproducibility of the work that we publish. This form provides structure for consistency and transparency in reporting. For further information on Nature Portfolio policies, see our Editorial Policies and the Editorial Policy Checklist.

Statistics

For all statistical analyses, confirm that the following items are present in the figure legend, table legend, main text, or Methods section.

n/a	Confirmed	
☐	☒	The exact sample size (*n*) for each experimental group/condition, given as a discrete number and unit of measurement
☐	☒	A statement on whether measurements were taken from distinct samples or whether the same sample was measured repeatedly
☐	☒	The statistical test(s) used AND whether they are one- or two-sided<br>*Only common tests should be described solely by name; describe more complex techniques in the Methods section.*
☒	☐	A description of all covariates tested
☒	☐	A description of any assumptions or corrections, such as tests of normality and adjustment for multiple comparisons
☐	☒	A full description of the statistical parameters including central tendency (e.g. means) or other basic estimates (e.g. regression coefficient) AND variation (e.g. standard deviation) or associated estimates of uncertainty (e.g. confidence intervals)
☐	☒	For null hypothesis testing, the test statistic (e.g. *F*, *t*, *r*) with confidence intervals, effect sizes, degrees of freedom and *P* value noted<br>*Give P values as exact values whenever suitable.*
☒	☐	For Bayesian analysis, information on the choice of priors and Markov chain Monte Carlo settings
☒	☐	For hierarchical and complex designs, identification of the appropriate level for tests and full reporting of outcomes
☒	☐	Estimates of effect sizes (e.g. Cohen's *d*, Pearson's *r*), indicating how they were calculated

Our web collection on statistics for biologists contains articles on many of the points above.

Software and code

Policy information about availability of computer code

Data collection	No software or or code was used for data collection.
Data analysis	All software used in this study is publicly available and cited in the Methods. Versions used are listed below. RNA-seq preprocessing and quality control: FastQC v0.11.9, MultiQC v1.11, SortMeRNA v4.3.4, Trimmomatic v0.39. Expression quantification and analysis: Salmon v1.6.0, DESeq2 v1.48.1. Orthology inference and comparative analysis: OrthoFinder v2.5.2. Network and clique analysis: igraph v2.1.2. ATAC-seq preprocessing: Trimmomatic v0.39, Bowtie2 v2.4.5, Samtools v1.16, MACS3 v3.0.0b1. Regulatory network analysis: MEME suite v5.5.5 (FIMO), Cytoscape v3.10.3. Structure prediction: AlphaFold3 (DeepMind/Isomorphic Labs, database release 2025-04-15).<br><br>All custom R code are available at https://github.com/ellendim/EVOTREE.

For manuscripts utilizing custom algorithms or software that are central to the research but not yet described in published literature, software must be made available to editors and reviewers. We strongly encourage code deposition in a community repository (e.g. GitHub). See the Nature Portfolio guidelines for submitting code & software for further information.

Data

Policy information about availability of data

All manuscripts must include a data availability statement. This statement should provide the following information, where applicable:

- Accession codes, unique identifiers, or web links for publicly available datasets
- A description of any restrictions on data availability
- For clinical datasets or third party data, please ensure that the statement adheres to our policy

Raw RNA-Seq reads were submitted to the European Nucleotide Archive (http://www.ebi.ac.uk/ena/) under accession numbers ERP016242 (Populus tremula), E-MTAB-14145 (Betula pendula), E-MTAB-14172 (Prunus avium), E-MTAB-14173 (Picea abies), E-MTAB-14175 (Pinus sylvestris) and E-MTAB-14176 (Pinus contorta). ATAC-seq: accession number PRJEB106577

Research involving human participants, their data, or biological material

Policy information about studies with human participants or human data. See also policy information about sex, gender (identity/presentation), and sexual orientation and race, ethnicity and racism.

Reporting on sex and gender	NA
Reporting on race, ethnicity, or other socially relevant groupings	NA
Population characteristics	NA
Recruitment	NA
Ethics oversight	NA

Note that full information on the approval of the study protocol must also be provided in the manuscript.

Field-specific reporting

Please select the one below that is the best fit for your research. If you are not sure, read the appropriate sections before making your selection.

☒ Life sciences ☐ Behavioural & social sciences ☐ Ecological, evolutionary & environmental sciences

For a reference copy of the document with all sections, see nature.com/documents/nr-reporting-summary-flat.pdf

Life sciences study design

All studies must disclose on these points even when the disclosure is negative.

Sample size	To quantify gene expression across wood formation, 15-µm thick longitudinal tangential cryosections were obtained across developing phloem and wood-forming tissues. The cryosections were then pooled into 25-28 samples per replicate tree: single sections were used within the cambial meristem region, pools of three sections representing expanding and secondary cell wall (SCW)-forming xylem, and pools of nine sections capturing maturing xylem (Tab. S1).
Data exclusions	Samples were inspected using hierarchical clustering and principal component analysis (PCA), and outliers were removed iteratively (i.e. normalization was redone, removed samples are marked in Tab. S1)
Replication	For each species, three to four replicate trees were sampled from clonal copies of a single genotype, with all replicates being mature trees growing in the field.
Randomization	NA
Blinding	NA

Reporting for specific materials, systems and methods

We require information from authors about some types of materials, experimental systems and methods used in many studies. Here, indicate whether each material, system or method listed is relevant to your study. If you are not sure if a list item applies to your research, read the appropriate section before selecting a response.

Materials & experimental systems

n/a	Involved in the study
☒	☐ Antibodies
☒	☐ Eukaryotic cell lines
☒	☐ Palaeontology and archaeology
☒	☐ Animals and other organisms
☒	☐ Clinical data
☒	☐ Dual use research of concern
☐	☒ Plants

Methods

n/a	Involved in the study
☒	☐ ChIP-seq
☒	☐ Flow cytometry
☒	☐ MRI-based neuroimaging

Dual use research of concern

Policy information about [dual use research of concern]

Hazards

Could the accidental, deliberate or reckless misuse of agents or technologies generated in the work, or the application of information presented in the manuscript, pose a threat to:

No	Yes
☒	☐ Public health
☒	☐ National security
☒	☐ Crops and/or livestock
☒	☐ Ecosystems
☒	☐ Any other significant area

Experiments of concern

Does the work involve any of these experiments of concern:

No	Yes
☒	☐ Demonstrate how to render a vaccine ineffective
☒	☐ Confer resistance to therapeutically useful antibiotics or antiviral agents
☒	☐ Enhance the virulence of a pathogen or render a nonpathogen virulent
☒	☐ Increase transmissibility of a pathogen
☒	☐ Alter the host range of a pathogen
☒	☐ Enable evasion of diagnostic/detection modalities
☒	☐ Enable the weaponization of a biological agent or toxin
☒	☐ Any other potentially harmful combination of experiments and agents

Plants

Seed stocks	Wood samples from Betula pendula, Pinus sylvestris, and Picea abies were obtained at a clonal field trial in Skogforsk, Sävar (63.895648°N, 20.549005°E). Betula pendula samples from genotype 18 were taken on July 8th, 2020, between 10:30–13:30. Pinus sylvestris samples from genotype 29 were taken on July 8th, 2020, between 13:30–14:30. Picea abies samples from genotype K27-2125 were taken on July 10th, between 10:30–12:00. Wood samples from Pinus contorta from genotype 86 were obtained at the clonal field trial in Fagerlund (plot 7114, 64.083393°N, 19.793286°E), on July 9th, 2020, between 10:30–12:00. Wood samples from Prunus avium from genotype S21-K946 6006 were obtained from Skogforsk, Ekebo (55.950860°N, 13.112653°E), on June 28th, 2021, between 10:00–12:00. No obvious abiotic or biotic stresses were observed at the time of sampling. Rectangular wood blocks were collected from each tree, immediately placed in dry ice boxes and stored at -70 ºC.
Novel plant genotypes	NA
Authentication	NA
